# Design, Synthesis, Evaluation, and Molecular Dynamics Simulation of Tadalafil Derivatives for the Treatment of Erectile Dysfunction

**DOI:** 10.3390/molecules31203785

**Published:** 2026-10-21

**Authors:** Wentao Liu, Bin Zhang, Yuping Jia, Xiangyu Kong, Yuxiao Deng, Xinqiang Cui, Li Yang, Wenjie Ren, Xuan Tang, Daizhou Zhang, Lingwen Xu

**Affiliations:** 1 Institute of Chemical Drugs, Shandong Academy of Pharmaceutical Sciences, Jinan 250101, China; 2 Shandong Engineering Research Center of Pharmaceutical Green Intelligent Technology, Shandong Academy of Pharmaceutical Sciences, Jinan 250101, China

**Keywords:** erectile dysfunction, tadalafil, phosphodiesterase type-5A, molecular dynamics simulations, structure–activity relationship

## Abstract

Erectile dysfunction (ED) remains a global health burden, yet the clinical utility of tadalafil is constrained by its poor aqueous solubility, cross-inhibition of homologous PDE isoforms, and an efficacy ceiling that cannot be overcome by dose adjustment alone. Herein, a series of diketopiperazine-based tadalafil analogues were designed and synthesized by decorating the amide nitrogen with flexible carbon chains and polar fragments (glycine, taurine, cysteine, and succinic/glutaric anhydride spacers), and by replacing the 3,4-methylenedioxybenzyl motif with substituted aromatic aldehydes. All target compounds were characterized by ^1^H/^13^C NMR and HR-MS and evaluated for cytotoxicity against rat aortic smooth muscle cells, calculated Log P and aqueous solubility, NO release, and phosphodiesterase type-5A (PDE5A) inhibitory activity, followed by 100 ns molecular dynamics (MD) simulations and MM/PBSA calculations on the leading analogue. Compound **18**, bearing a *p-methoxyphenyl* group and an *N*-methyl amide, markedly enhanced NO release relative to tadalafil, reduced residual PDE5A activity to 38.69 ± 1.98% at 10 μM, and exhibited negligible cytotoxicity with a predicted logP of 1.53. Docking into PDE5 (PDB: 1XOZ) and MD analysis revealed a stable binding pose anchored by hydrogen bonds to Gln817 and Tyr612, reinforced by π–π stacking with Phe820 and extensive hydrophobic contacts with Leu765, Ile768, Val782, and Met816 (ΔG_bind = −9.13 ± 3.30 kcal/mol), with van der Waals contributions dominating (−41.78 ± 4.60 kcal/mol). Collectively, compound **18** emerges as a promising next-generation tadalafil-derived PDE5 inhibitor lead, and the resulting SAR picture outlines a hydrophobic-anchor-dominated optimization strategy for this scaffold.

## 1. Introduction

Erectile dysfunction (ED) is a highly prevalent chronic urogenital disease worldwide and has been designated by the World Health Organization as a priority public health issue affecting the quality of life of middle-aged and elderly men [1,2]. Currently, the global prevalence of ED among men over 40 years of age exceeds 52%, with moderate-to-severe cases accounting for more than 30% [3,4]. Moreover, the onset of ED is trending toward younger populations, with the prevalence rate among individuals aged 20–39 years having increased by over 40% in the past decade, resulting in a continuously escalating disease burden [3,4].

Tadalafil, a highly selective phosphodiesterase type 5 (PDE5) inhibitor approved by the U.S. Food and Drug Administration (FDA) in 2003, has become the most prescribed first-line therapeutic agent for ED worldwide, owing to its 36-h half-life and pharmacokinetic profile that is unaffected by high-fat meals [1,5]. In recent years, its therapeutic indications have continued to expand: it has been approved for the treatment of pulmonary arterial hypertension and has demonstrated potential value in multiple domains, including the amelioration of lower urinary tract symptoms associated with benign prostatic hyperplasia and the restoration of vascular endothelial function [6,7,8]. Notably, the once-daily 5 mg low-dose regimen of tadalafil is the only PDE5 inhibitor regimen recommended by international guidelines for the long-term chronic management of ED, and its long-term safety has been validated by large-scale real-world studies [9,10]. Nevertheless, the tadalafil scaffold retains significant limitations. First, its target selectivity is insufficient; cross-inhibition of homologous PDE isoforms, including PDE1, PDE6, and PDE11, can induce adverse reactions such as headache, transient visual sensitivity reduction, back pain, and myalgia, thereby compromising patient adherence [11,12]. Second, substantial inter-individual pharmacokinetic variability exists, with nearly 20% of patients exhibiting primary non-response [13]. Third, absolute contraindications for co-administration remain: tadalafil cannot be used in patients with coronary artery disease who are concurrently taking nitrates, and its use in patients with severe hepatic or renal impairment carries considerable risk. Furthermore, the pharmacodynamic modulation window of the existing scaffold has reached a plateau, and adjustments to dosing regimens alone cannot overcome the efficacy ceiling [14].

Over the past decade, extensive structural modification efforts have been undertaken globally on tadalafil derivatives, progressing from early side-chain modifications of the parent scaffold to site-directed multi-region modifications, and more recently to the development of multifunctional hybrid molecules and water-soluble prodrugs [15,16,17]. Although several candidate compounds with superior in vitro activity relative to the parent molecule have been identified, existing studies generally suffer from three major shortcomings: (1) validation limited to in vitro enzymatic assays, (2) lack of rational design guidance, and (3) failure to elucidate dynamic binding regulatory mechanisms [15,16,17]. Consequently, no substantive breakthrough in druggability enhancement has been achieved to date. 

Against this backdrop, the rational design, synthesis, and systematic activity evaluation of novel tadalafil derivatives carry urgent clinical value, academic significance, and industrial translation prospects. Such efforts can address existing gaps in clinical treatment by providing safer therapeutic options for ED patients with comorbid underlying diseases, refine the structure–activity relationship (SAR) landscape of this compound class, and yield proprietary next-generation lead compounds, thereby expanding the research boundaries of the tadalafil scaffold. The present study aims to design and synthesize a series of novel tadalafil derivatives through targeted approaches, systematically evaluate them across multiple dimensions, and identify one to two candidate compounds with significantly superior druggability compared with the parent molecule. The findings are expected to elucidate novel SAR principles and provide both experimental and theoretical support for the development of next-generation PDE5 inhibitors.

## 2. Results and Discussion

### 2.1. Chemical Synthesis

**Figure 1:**
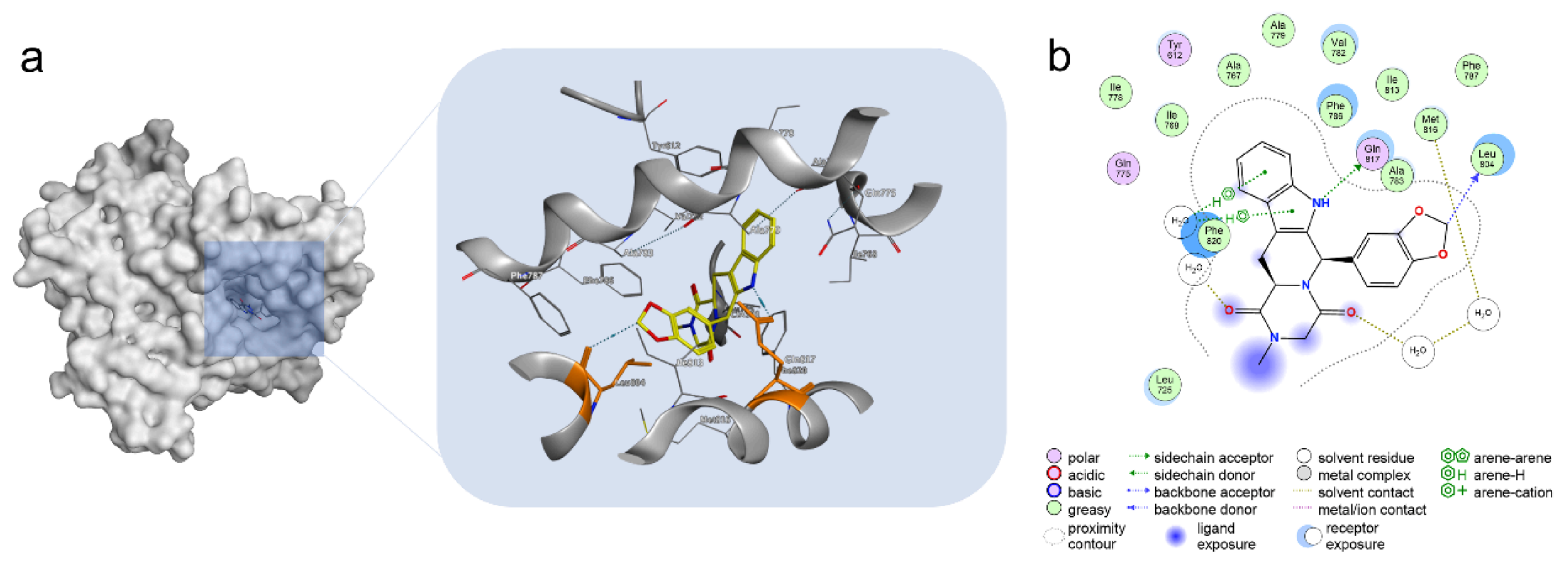
Key binding information of tadalafil with the PDE5 protein (PDB: 1XOZ). (**a**) Three-dimensional structure of the tadalafil–PDE5 protein–ligand complex. (**b**) Two-dimensional ligand–residue interaction diagram.

**Figure 2:**
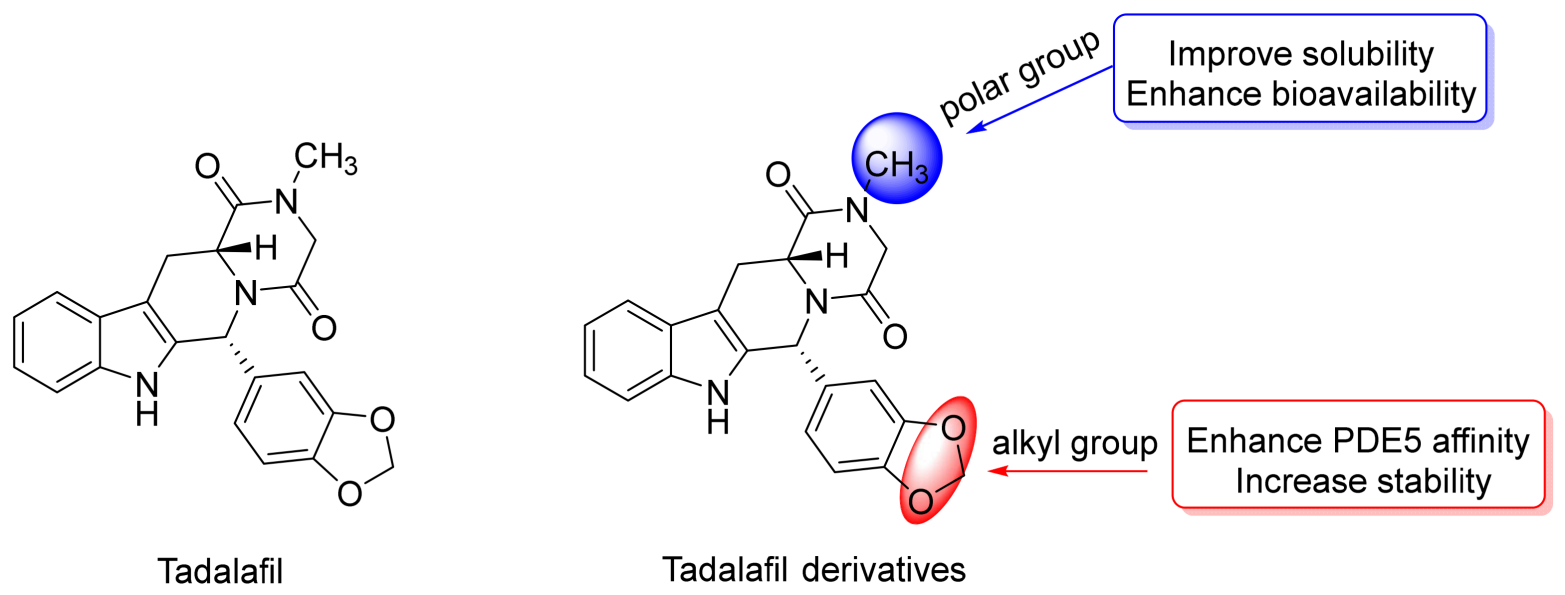
The structures of Tadalafil and Tadalafil derivatives.

**Figure 3:**
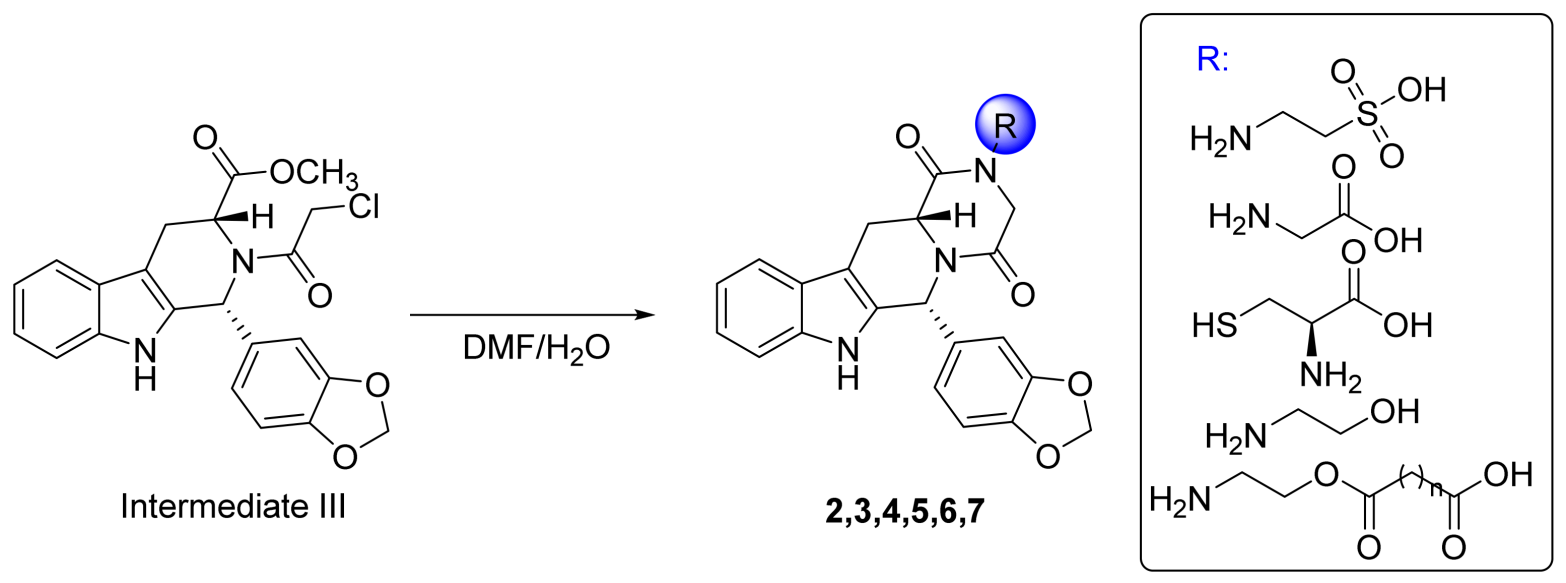
The synthesis route 1 of Tadalafil derivatives.

**Figure 4:**
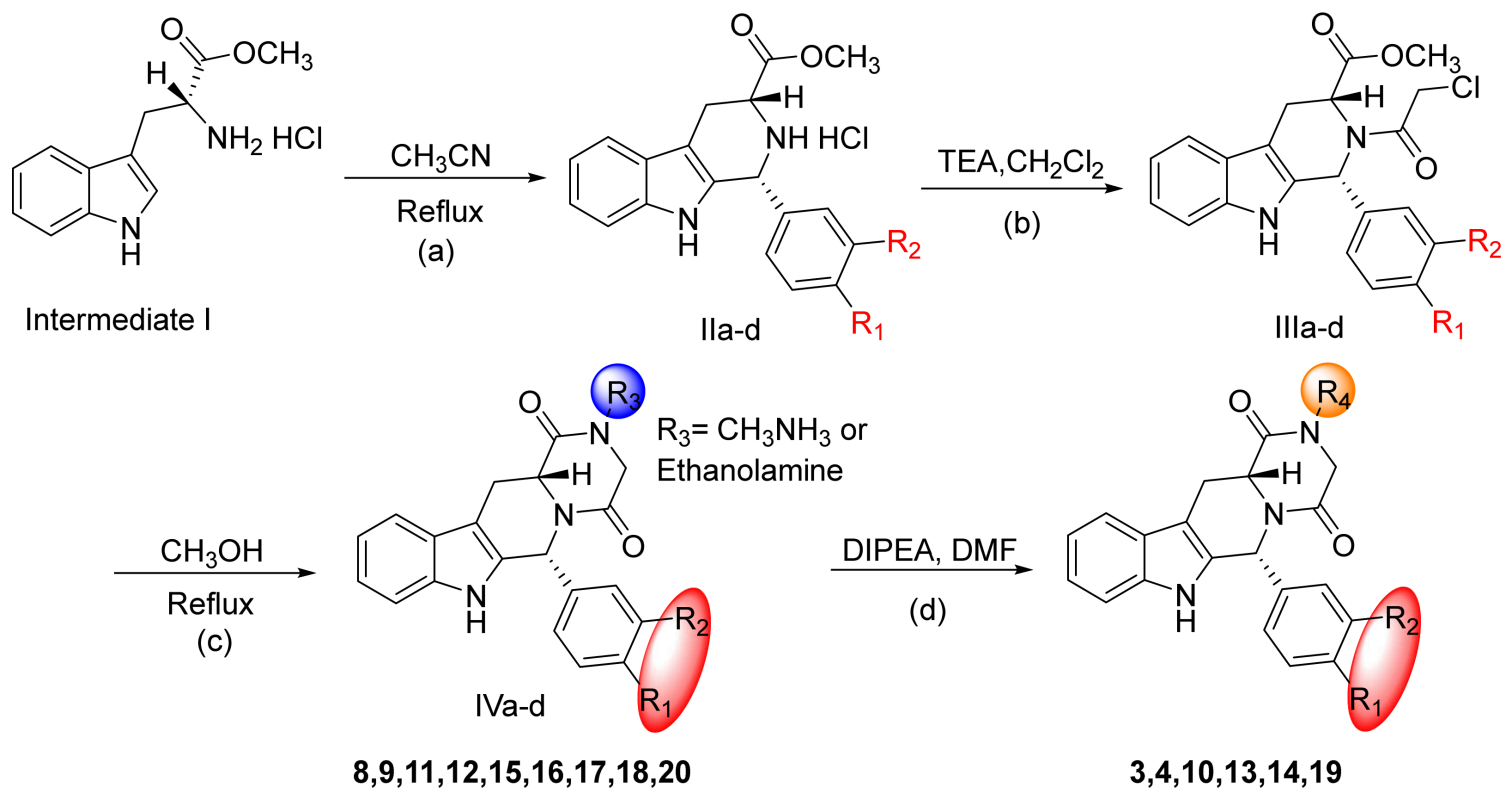
The synthesis route 2 of tadalafil derivatives. (**a**) Aromatic aldehydes (p-Anisaldehyde or 3,4-diethoxybenzaldehyde or 4-Ethoxy-3-methoxybenzaldehyde or 3-Ethoxy-4-hydroxybenzaldehyd), CH_3_CN, reflux, 60–70 °C, 6 h. (**b**) Chloroacetyl chloride, CH_2_Cl_2_, TEA, 5–7 °C, 30 min. (**c**) CH_3_NH_2_ or ethanolamine, CH_3_OH, 50–60 °C; (**d**) 4–6 h: succinic or glutaric anhydride, DIPEA, DMF, 70 °C, 6 h.

**Figure 5:**
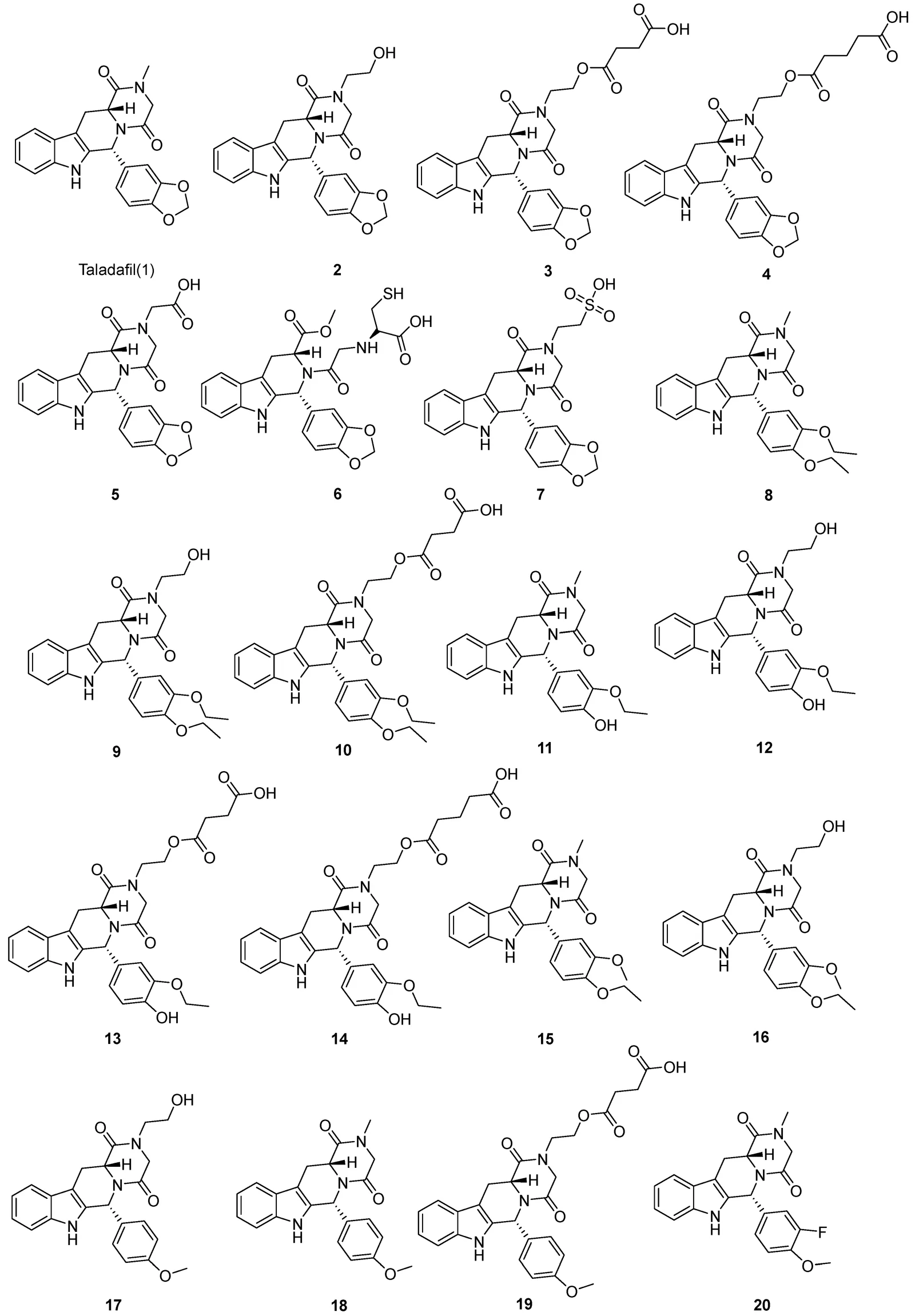
The structures of tadalafil derivatives.

Tadalafil, a widely used PDE5 inhibitor in clinical practice, offers several advantages, including a prolonged duration of action (half-life of 17.5 h) and no interference from food intake (Figure 1). Nevertheless, it is not without limitations. Its oral bioavailability is relatively low (approximately 36%), largely due to the hydrophobic nature of its molecular structure, which results in incomplete absorption [18]. In some patients, higher doses are required to achieve therapeutic plasma concentrations, thereby increasing the risk of adverse effects. This study was based on the parent structure of tadalafil. Through structural optimization of key intermediates I and III, flexible alkyl spacers terminating in polar fragments were introduced at the amide nitrogen to enhance polarity and aqueous developability. In addition, the 3,4-methylenedioxybenzyl motif was replaced with substituted aromatic aldehydes to remodel the hydrophobic and aromatic contacts within the PDE5 catalytic pocket, leading to the design and synthesis of a series of novel tadalafil derivatives. Leveraging the established, environmentally friendly synthetic route previously developed in our laboratory, and aiming to enhance the physicochemical properties and bioavailability of tadalafil, either intermediate I or III was used as the starting material [18]. Structural modifications were introduced at the amino group and the piperonal moiety, incorporating polar groups, structurally diverse aromatic aldehydes, extended carbon chains, and green amino acid fragments (e.g., glycine, taurine, cysteine, etc.) (Figure 2). The objective was to design and synthesize a series of PDE5 inhibitors with improved solubility, high binding affinity and inhibitory activity toward PDE5, and reduced toxicity and side effects (Figure 2, Figure 3, Figure 4 and Figure 5).

### 2.2. Cytotoxicity Assay–CCK-8

After completing the synthesis and characterization of the compounds, we assessed the cytotoxic effects of the tadalafil derivatives on normal cells using rat aortic smooth muscle cells (RASMCs), with tadalafil serving as the positive control. Cells were seeded in 96-well plates at a density of 3000 cells per well and allowed to adhere overnight. The cells were then exposed to serial two-fold dilutions of the compounds for 72 h, followed by the addition of 10 μL of CCK-8 reagent to each well to determine cell viability. The resulting IC_50_ values indicated that, except for derivatives **5**, **6**, **8**, **11**, and **13**—which showed effects on RASMC proliferation relative to tadalafil—all other derivatives exhibited low cytotoxicity under the tested in vitro conditions (Table 1).

### 2.3. Prediction of Lipid–Water Partition Coefficient and Aqueous Solubility

In this section, the lipid–water partition coefficients and aqueous solubility of the synthesized tadalafil derivatives were predicted using MOE and EPI-Suite software (v4.1), respectively. The predicted data showed that, with the exception of compound **7**, all synthesized derivatives had lipid–water partition coefficients within the reasonable range of 0–3 (Table 1) [19]. Many erectile dysfunction therapeutics, such as PDE5 inhibitors like sildenafil and tadalafil, are inherently hydrophobic and exhibit poor aqueous solubility. Enhanced solubility enables drugs to dissolve more rapidly in the gastrointestinal tract, producing higher-concentration solutions that increase contact with the gastrointestinal mucosa, thereby promoting passive diffusion and absorption, and ultimately improving oral bioavailability. To address tadalafil’s poor aqueous solubility, two solubility prediction models—WSKOW v1.42 and Fragment Wat Sol (v1.01 est)—were employed. The results revealed that most derivatives displayed markedly improved aqueous solubility compared with tadalafil (Table 1). Additionally, molecular docking of all derivatives was conducted using the crystal structure of the PDE5–tadalafil complex (PDB ID: 1XOZ) to evaluate docking scores toward PDE5. The docking results indicated that extending the carbon chain at the amide nitrogen position of the derivatives contributed to enhanced docking scores for the PDE5 target.

### 2.4. Determination of NO Release

**Figure 6:**
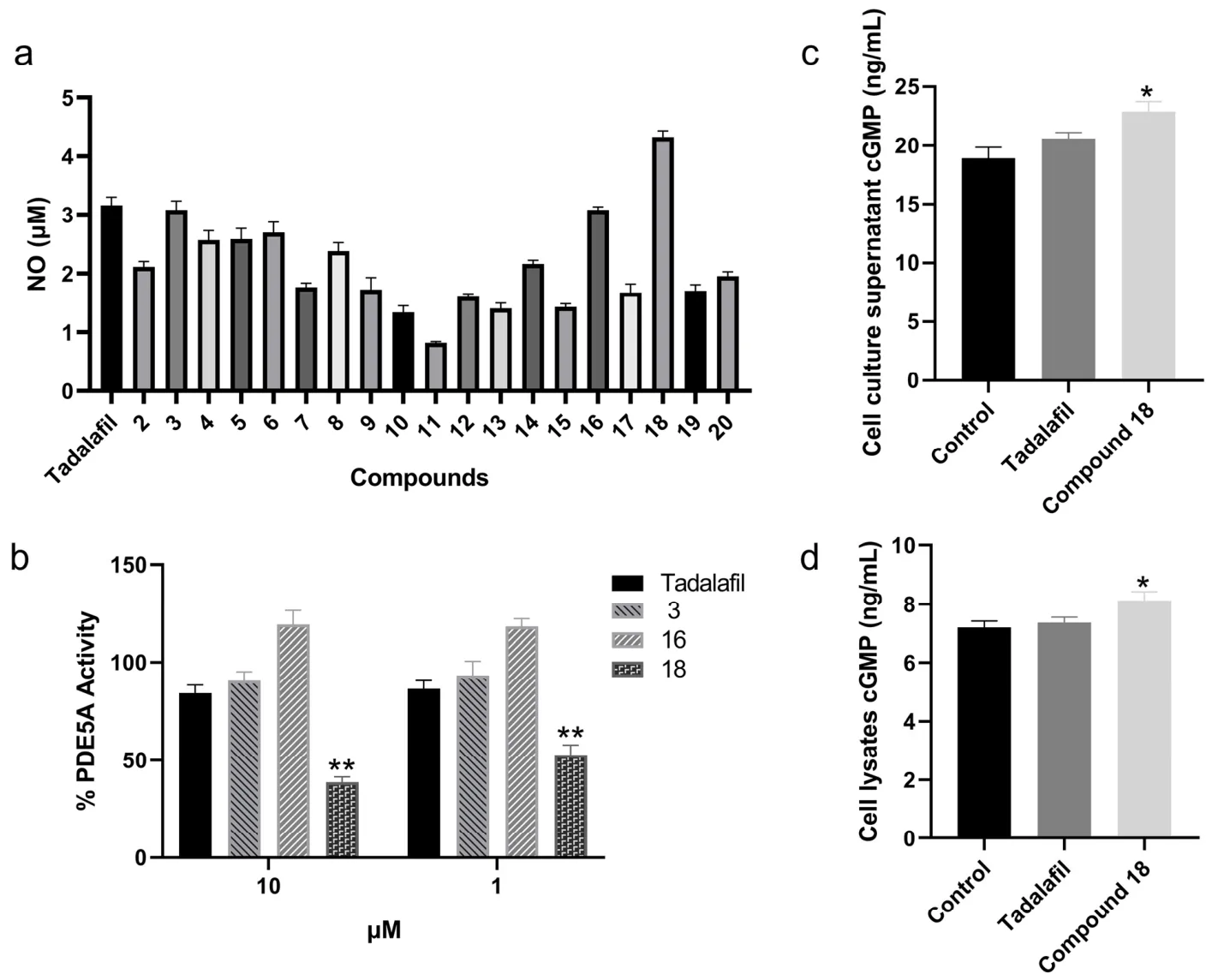
Evaluation of the Biological Activity of Tadalafil Derivatives. (**a**) Effects of tadalafil derivatives on nitric oxide (NO) release from rat aortic smooth muscle. (**b**) Inhibitory activity levels of different potential PDE5 inhibitors against the PDE5 protein. ** *p* < 0.01, compared with the Tadalafil group. Cell culture supernatant (**c**) and cell lysates (**d**) of RASMC cGMP levels. * *p* < 0.05, compared with the control group. Data are presented as mean values ± standard error of the mean (*n* = 3).

PDE5 inhibitors and nitric oxide (NO) are closely intertwined in the physiological regulation of male erection, functioning in a synergistic and interdependent manner. In brief, NO acts as the signaling molecule that initiates erection, while PDE5 inhibitors serve as a protective mechanism that prevents this signal from being rapidly degraded. In our assays, compound **18** markedly enhanced NO release relative to the positive control tadalafil (Figure 6a). This increase in NO served as a crucial upstream event, facilitating the subsequent rise in cGMP levels, which in turn promoted corpus cavernosum smooth muscle relaxation, vasodilation, and enhanced blood inflow. Notably, the experimental findings revealed that extending the carbon chain at the amide nitrogen position or introducing polar groups in the structural modifications of tadalafil did not elevate NO levels in the resulting derivatives (Figure 6a). This suggests that substituents introduced at the amide nitrogen failed to engage key functional residues of PDE5, thereby compromising the intended efficacy of the derivative design.

Additionally, when m-anisaldehyde was substituted for piperonal, with all other positions unchanged, intervention with the derivative significantly enhanced NO release in rat aortic smooth muscle cells.

### 2.5. Analysis of PDE5A Inhibitory Activity

Based on the results of the NO assay, compounds **3**, **16**, and **18** were selected for further evaluation of their inhibitory activity against PDE5A. Enzyme activity was measured using a fluorescence polarization assay kit (BPS Bioscience, San Diego, CA, USA). In brief, PDE5A1 enzyme was incubated with the FAM-labeled cyclic-3′,5′-GMP substrate at room temperature for 1 h in the presence of the test compounds. The reaction was then terminated by adding a PDE binding agent, followed by an additional 30-min incubation. Fluorescence polarization values were subsequently recorded using a microplate reader. Compound stock solutions were prepared. At concentrations of 10 μM and 1 μM, compound 18 reduced PDE5A activity to 38.69% ± 1.98% and 52.79% ± 3.54%, respectively, indicating more potent inhibition than tadalafil at both concentrations (Figure 6b). These results provide preliminary evidence and a solid basis for the continued development of tadalafil derivatives with enhanced activity.

### 2.6. Determination of cGMP Level

Nitric oxide (NO) stimulates cGMP production in smooth muscle cells, a key signaling molecule that promotes vascular smooth muscle relaxation. To determine whether compound **18** promotes cGMP expression by enhancing NO release, thereby facilitating corpus cavernosum erection through smooth muscle relaxation, we measured cGMP levels in both the culture supernatants and cell lysates of rat aortic smooth muscle cells using a rat cyclic guanosine monophosphate (cGMP) ELISA kit. Cells were treated with 10 μM compound **18** or tadalafil for 0.5 h, followed by stimulation of particulate guanylate cyclase with atrial natriuretic factor (0.1 μmol/L) at 37 °C for 10 min. cGMP concentrations in the culture supernatants and cell lysates were then determined using the kit. The ELISA results showed that at a concentration of 10 μM, compound **18** significantly increased cGMP levels in both the culture supernatants and cell lysates compared with the positive control tadalafil, reaching concentrations of 22.90 ± 0.598 ng/mL and 8.13 ± 0.21 ng/mL, respectively (Figure 6c,d). These findings further confirm the pathway and mechanism by which this class of compounds intervenes in erectile dysfunction.

### 2.7. Molecular Dynamics Simulation of Candidate Compound

#### 2.7.1. Thermodynamic Equilibrium and System Stability

**Figure 7:**
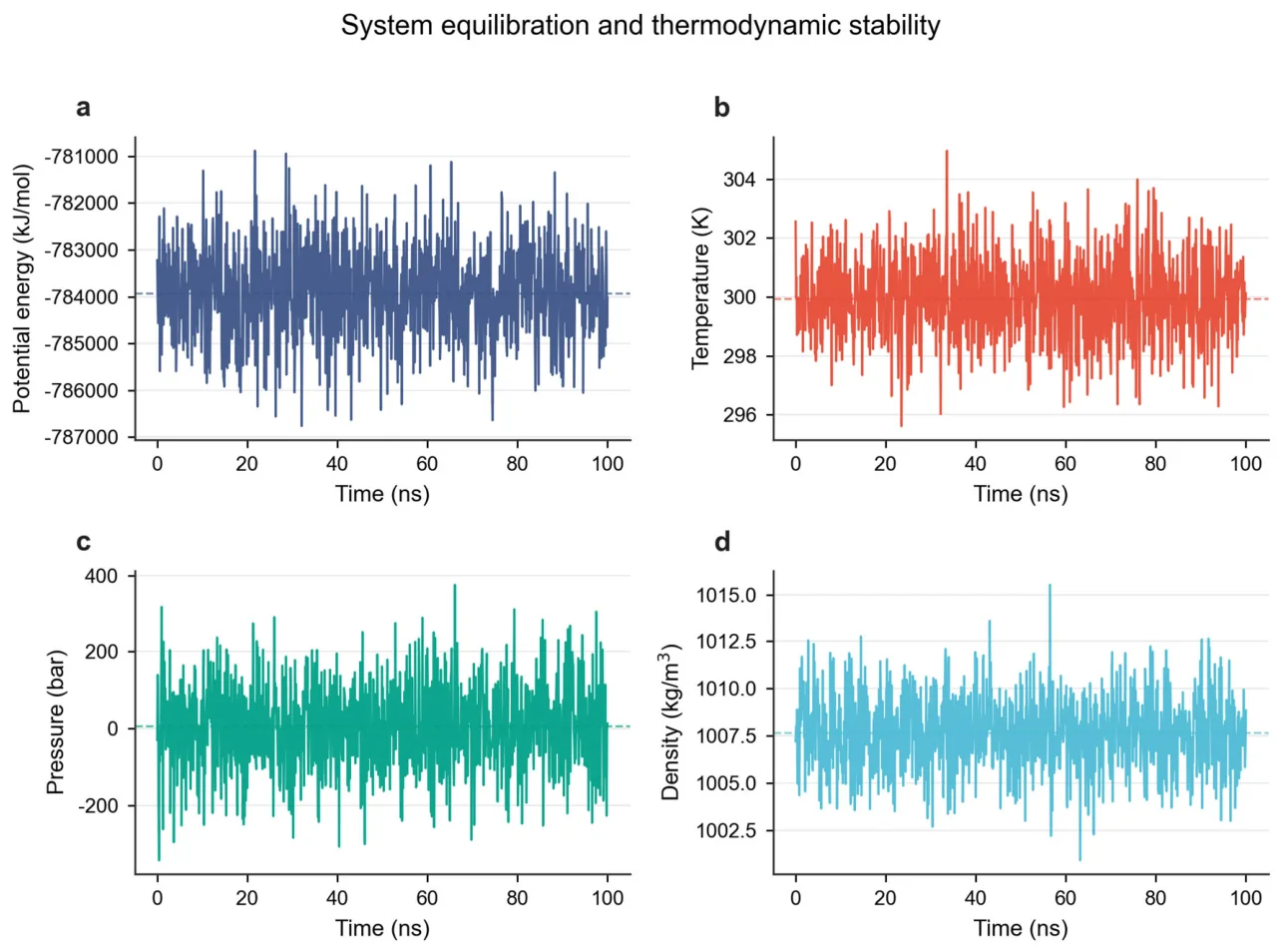
Thermodynamic monitoring during the 100 ns production dynamics simulation. (**a**) Potential energy; (**b**) temperature; (**c**) pressure; (**d**) density. Dashed lines represent the mean values over the last 50 ns.

Based on the above experimental results, molecular dynamics (MD) simulations were performed on compound **18** in complex with the PDE5 target protein to further validate their interaction. According to the MD results, throughout the production dynamics trajectory, the potential energy and total energy fluctuated around stable baselines without persistent drift (Figure 7a). During the final 50 ns, the average temperature was 299.94 ± 1.44 K, which was highly consistent with the set value of 300 K; the average density was 1007.66 ± 1.90 kg/m^3^, with minimal fluctuation (SD~1.90), indicating that the solvated box volume and density had fully converged (Figure 7b). The pressure exhibited typical large-amplitude fluctuations on the instantaneous scale (mean value over the final 50 ns: 6.6 ± 112.5 bar), consistent with the short-term pressure noise characteristics of the NPT ensemble; its time-averaged value approached the target pressure and did not constitute evidence of system instability (Figure 7c,d). Although the current single long-trajectory MD simulation was performed to rationalize the experimentally validated binding pose of the leading analog rather than to provide replica-averaged free-energy ranking across the full compound series, we acknowledge that the absence of multiple independent short MD replicas may limit the statistical robustness of the observed pose stability results. Running multiple parallel short MD replicas to further validate the consistency of binding interactions across independent trajectories will be an essential follow-up in our subsequent research.

#### 2.7.2. RMSD: Overall Conformational Stability

**Figure 8:**
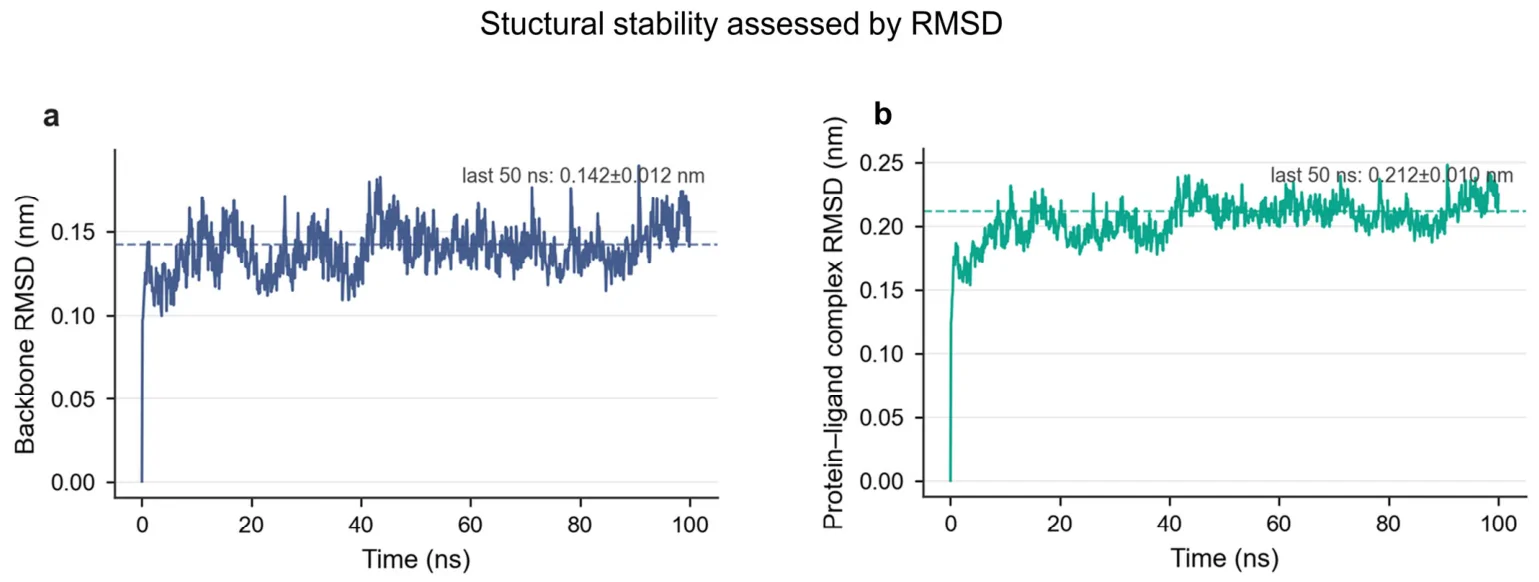
Conformational stability during 100 ns MD. (**a**) Protein backbone RMSD after least-squares fitting to the initial backbone. (**b**) Protein–ligand complex RMSD after fitting (shown only to illustrate that inclusion of the ligand does not inflate global deviation). Dashed lines indicate means over the final 50 ns.

Using the initial structure as the reference and performing least-squares superposition, the backbone RMSD rapidly reached a plateau following a brief initial increase. Over the final 50 ns, the backbone RMSD was 0.142 ± 0.012 nm, the Cα RMSD was 0.144 ± 0.012 nm, the protein RMSD was 0.212 ± 0.010 nm, and the protein–ligand complex RMSD was 0.212 ± 0.010 nm (Figure 8). All of the above metrics remained stable within the range of approximately 0.14–0.21 nm, with SDs of only approximately 0.01 nm, indicating that the secondary/tertiary structure of the receptor remained highly stable over the 100 ns timescale, with no evidence of global unfolding or structural collapse (Figure 8). The complex RMSD was nearly identical to the protein RMSD, suggesting that the ligand contributed minimally to the overall superposition deviation and that conformational changes in the vicinity of the binding pocket remained within a controlled range (Figure 8).

#### 2.7.3. RMSF and Radius of Gyration: Local Flexibility and Global Compactness

**Figure 9:**
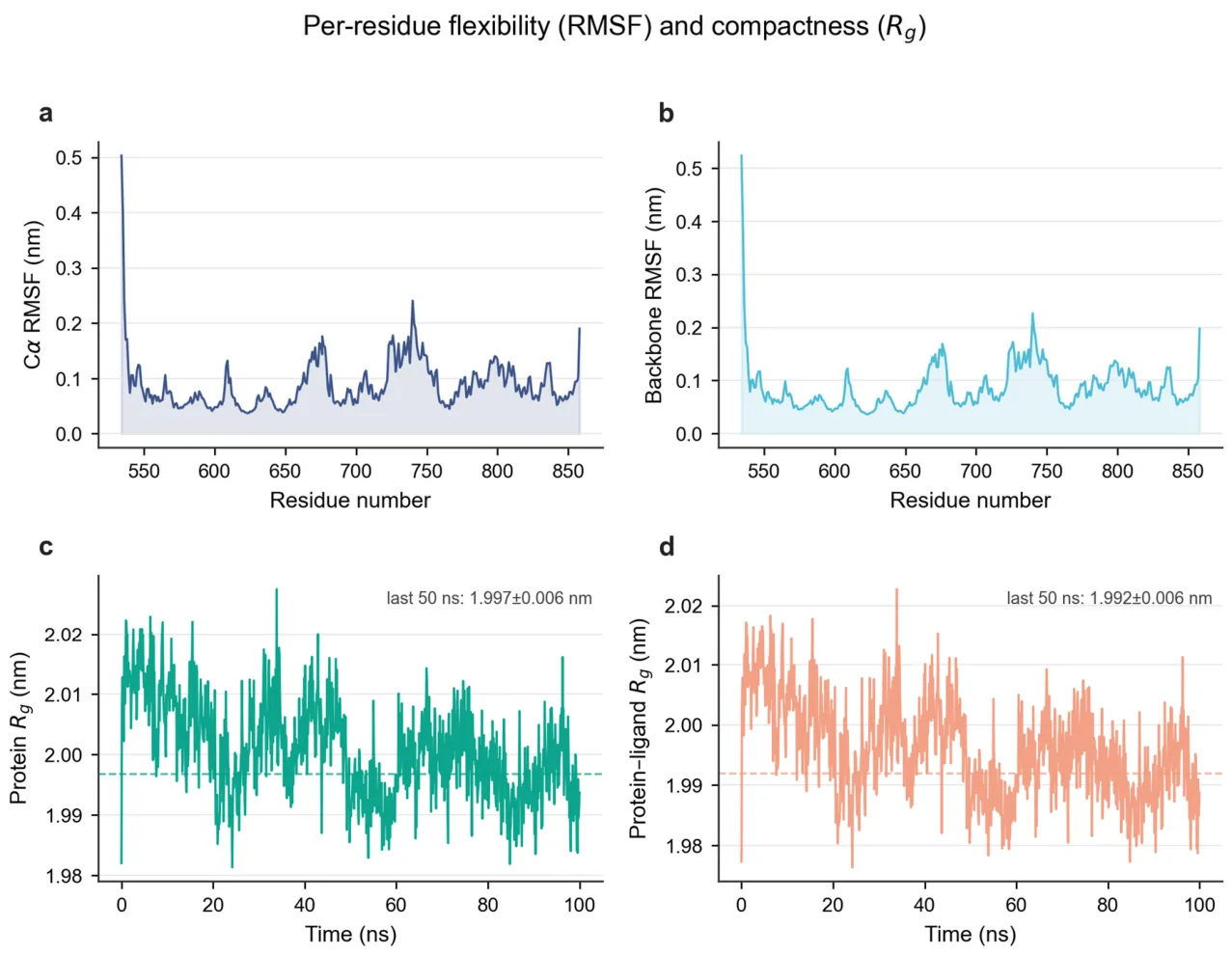
Local flexibility and overall compactness. (**a**) Cα RMSF; (**b**) backbone RMSF; (**c**) radius of gyration of the protein; (**d**) radius of gyration of the protein–ligand complex.

The mean Cα RMSF across all residues was 0.089 ± 0.047 nm, with the vast majority of residues showing fluctuations below 0.15 nm, consistent with the rigid-core behavior typical of a folded protein (Figure 9). The highest RMSF value, approximately 0.503 nm, was observed near residue 534—a region generally associated with loop segments, termini, or solvent-exposed areas—reflecting localized functional flexibility rather than global instability (Figure 9). Over the final 50 ns of simulation, the radius of gyration (Rg) was 1.997 ± 0.006 nm for the protein and 1.992 ± 0.006 nm for the complex (Figure 9). The minimal variation in both cases (SD~0.006 nm) indicates that secondary structure packing and the overall ellipsoidal shape remained highly stable throughout the sampling period, with no evidence of significant swelling or collapse. Comparative RMSF analysis against apo PDE5 and against a tadalafil-bound reference complex was not performed in this work. Therefore, the present RMSF profiles should be interpreted as descriptors of local flexibility within the compound **18** complex rather than as evidence of ligand-specific conformational remodeling.

#### 2.7.4. Hydrogen Bonds and SASA: Hydrogen Bond Network and Solvation Characteristics

**Figure 10:**
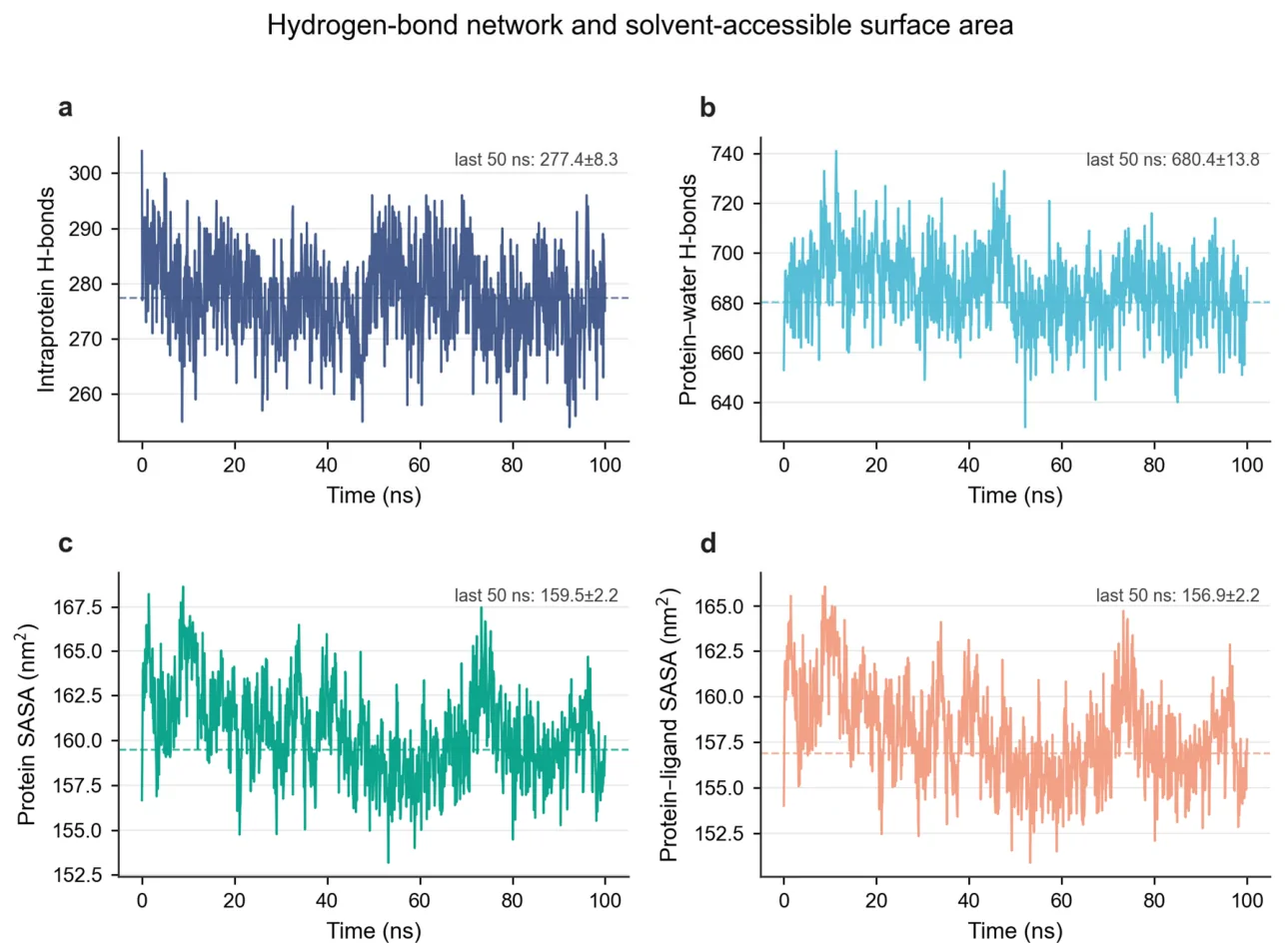
Hydrogen bonds and solvent-accessible surface area. (**a**) Number of intramolecular protein hydrogen bonds; (**b**) number of protein–water hydrogen bonds; (**c**) protein SASA; (**d**) protein–ligand complex SASA.

The number of intramolecular hydrogen bonds remained at 277.4 ± 8.3 over the final 50 ns, with relatively limited fluctuations, indicating that the hydrogen-bonding backbone of the secondary structure remained intact throughout the sampling process. The number of protein–water hydrogen bonds was 680.4 ± 13.8, exhibiting mild and stable fluctuations, reflecting dynamic exchange at the solvation interface while the overall degree of solvation remained stable (Figure 10).

The protein SASA over the final 50 ns was 159.48 ± 2.21 nm^2^, and the SASA of the protein–ligand complex was 156.88 ± 2.22 nm^2^ (Figure 10). The difference between the two values was approximately 2.60 nm^2^, suggesting that ligand occupancy converted part of the protein surface from solvent-accessible to buried, which is consistent with sustained protein–ligand contacts. The SASA time series showed no unidirectional drift, supporting that the solvation interface had reached a steady state.

#### 2.7.5. Protein–Ligand Distance/Contacts and PCA Collective Motion

**Figure 11:**
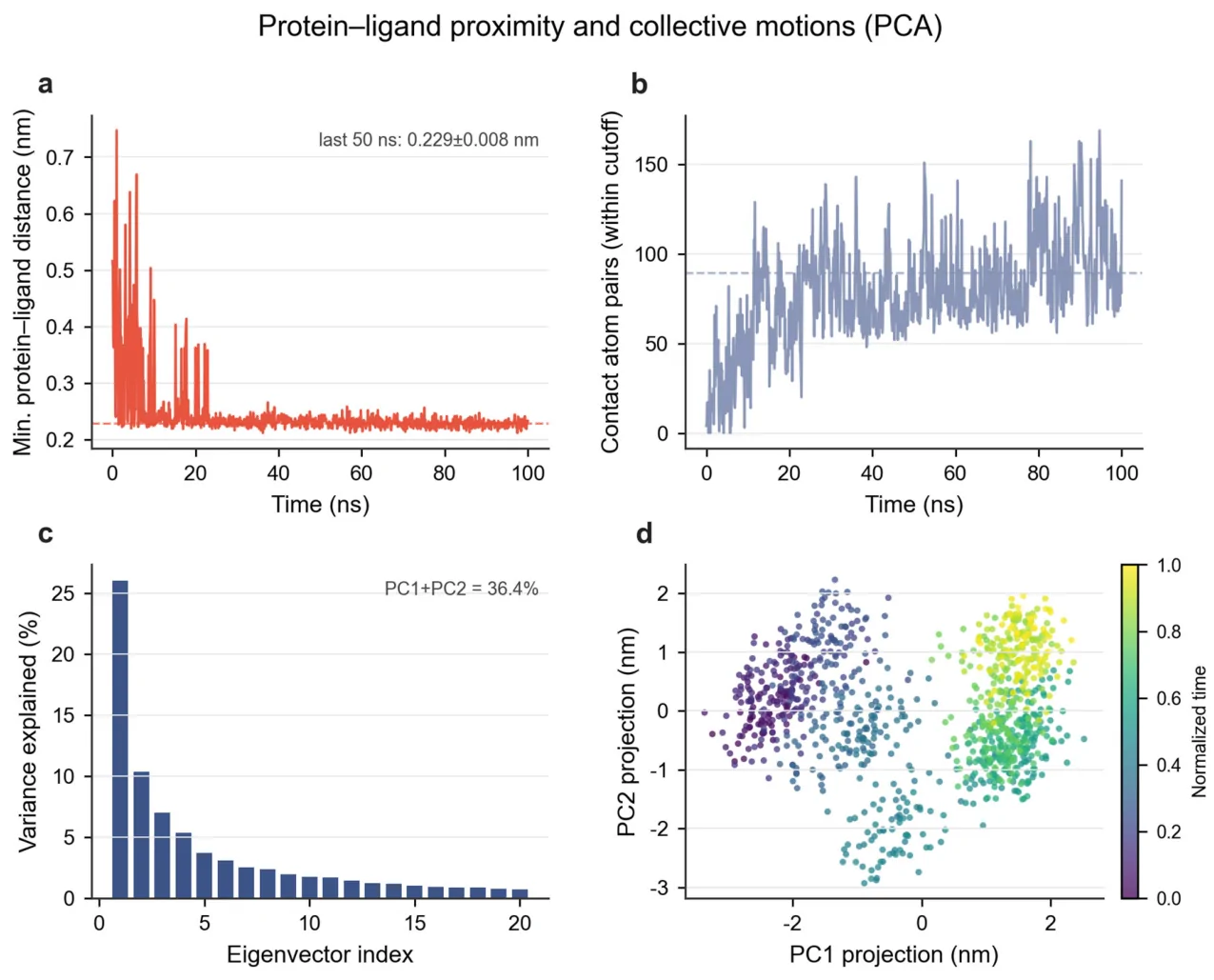
Protein–ligand proximity and principal component analysis. (**a**) Minimum distance; (**b**) number of contacting atom pairs; (**c**) variance contribution of the top 20 eigenmodes; (**d**) PC1–PC2 projection (color indicates normalized time).

The minimum protein–ligand distance stabilized rapidly after minor adjustments during the early stage of the simulation, with a value of 0.229 ± 0.008 nm over the final 50 ns, remaining consistently within the range of direct contact (far below the typical dissociation threshold), and no ligand escape events were observed (Figure 11). The number of contacting atom pairs within the cutoff averaged 89.6 ± 23.2 over the final 50 ns, further elevated relative to the overall trajectory mean (78.2), suggesting that the binding interface contacts became more extensive and stable during the latter half of the simulation (Figure 11). Taken together with the complex RMSD, Rg, SASA, and minimum distance results, it can be concluded that compound **18** remained stably accommodated within the binding site of PDE5.

Eigendecomposition of the backbone atomic covariance matrix revealed that PC1 and PC2 accounted for approximately 26.1% and 10.4% of the variance, respectively, with a cumulative contribution of 36.4% (Figure 11). The PC1–PC2 projection showed that the trajectory sampled continuously within a confined conformational space, with no evidence of transitions between multiple fully separated rigid states. The gradual color changes over time indicated that the system underwent moderate drift/oscillation along the dominant collective coordinates, consistent with the plateau phase observed in the RMSD profile. These results support the use of the final 50 ns (or a later segment) of the trajectory for representative conformation extraction and binding interface analysis.

#### 2.7.6. Binding Mode Diagram

The final averaged structure over the last 50–100 ns was extracted, and residues forming stable contacts with compound **18** as well as the types of interactions involved were annotated. Van der Waals interactions refer to non-directional, non-saturating, and relatively weak intermolecular forces in chemical interaction patterns. Van der Waals forces are electrostatic in nature but are considerably weaker than chemical bonds or hydrogen bonds, typically with energies below 5 kJ/mol. The magnitude of van der Waals forces is proportional to molecular size. Hydrogen bonds (HB) occur when a hydrogen atom covalently bonded to a highly electronegative atom X approaches another electronegative atom Y with a small atomic radius (e.g., O, F, N), forming a special intermolecular or intramolecular interaction of the form X–H···Y mediated by the hydrogen atom. When a positively charged atom associates with a negatively charged atom, the affinity between them becomes stronger, which is also referred to as a salt bridge (SB).

**Figure 12:**
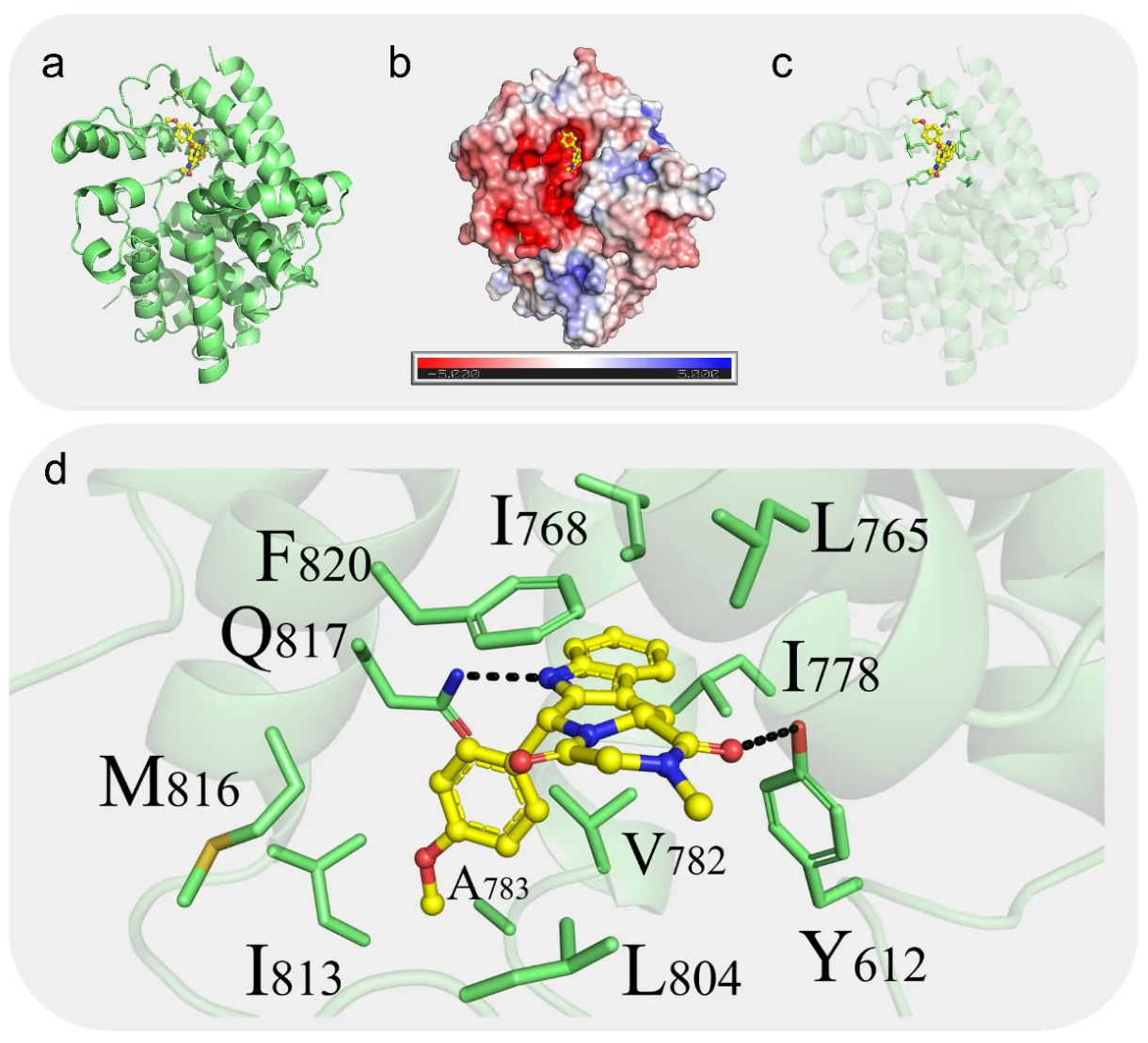
Molecular docking data of candidate compound **18** with the PDE5 protein. (**a**) Overall binding mode analysis in cartoon representation; (**b**) overall binding mode analysis in surface representation; (**c**) interaction mode shown in ball-and-stick representation; (**d**) amino acid residue interactions at the binding interface.

Binding site localization and core interaction network: Analysis based on the final molecular dynamics structure of the PDE5-**18** complex system (complex_final_100ns) revealed that compound **18** is stably anchored within the protein catalytic pocket. The figure shows that it forms close contacts with approximately 11 surrounding amino acid residues, constituting a well-defined polar–hydrophobic composite binding interface. The core polar anchoring is primarily contributed by two hydrogen-bonding networks: first, the indole nitrogen (N) of the ligand forms a geometrically favorable hydrogen bond with the side-chain carbonyl nitrogen (N) of Gln817, constituting the dominant directional polar lock; second, the phenolic hydroxyl oxygen (OH) of Tyr612 maintains a secondary polar contact of approximately 3.41 Å with the ligand carbonyl oxygen (O), providing auxiliary constraint on ligand orientation (Figure 12). The synergy between the aforementioned hydrogen bonds and surrounding hydrophobic residues suggests that the stability of this complex depends simultaneously on directional polar anchoring and hydrophobic pocket complementarity, rather than being dominated by a single driving force.

Aromatic stacking and hydrophobic pocket synergistic anchoring mechanism: In addition to polar anchoring, the fused-ring scaffold of compound **18** is further stabilized synergistically by aromatic stacking and dense hydrophobic encapsulation. The phenyl ring of Phe820 adopts a face-to-face π–π stacking orientation with the diketopiperazine/fused core of the ligand, with a ring centroid distance of approximately 3.78 Å and a shortest heavy-atom distance of approximately 3.23 Å, providing critical vertical clamping of the planar ligand scaffold. Concurrently, hydrophobic side chains including Leu765, Ile768, Ile778, Val782, Ala783, Leu804, Ile813, and Met816 are arranged circumferentially along the ligand carbon scaffold, forming a continuous hydrophobic lining (Figure 12). Carbon–carbon contact statistics (C···C < 4.5 Å) of the final-state conformation identified approximately 57 pairs of hydrophobic contacts, among which Ile768 and Val782 contributed particularly prominently (Figure 12). Thus, the aromatic stacking of Phe820 together with the aforementioned hydrophobic pocket collectively “embeds” the rigid fused ring of **18** within the binding cavity, constituting the primary spatial constraint and stabilization mechanism in addition to hydrogen bonding.

Summary of the binding mechanism and electrostatic assistance: Overall, the binding mode of compound **18** with the PDE5-derived protein exhibits a composite mechanism of “hydrogen bond-directed anchoring + aromatic/hydrophobic synergistic encapsulation”: Gln817 (and, to a lesser extent, Tyr612) provides polar positioning, Phe820 provides π–π vertical stabilization, and residues including L765/I768/I778/V782/A783/L804/I813/M816 accomplish lateral sealing through dense hydrophobic contacts (Figure 12). Polar side chains such as Gln817 also contribute local electrostatic complementarity beyond hydrogen bonding, serving an auxiliary corrective role in ligand orientation within the pocket. The multi-type interaction network formed by approximately 11 contacting residues collectively underpins the stable binding of the complex.

#### 2.7.7. MM/PBSA Binding Free Energy Component Analysis

MM/PBSA energy decomposition revealed that the total binding free energy (ΔG_bind) of the PDE5–**18** complex was −9.13 ± 3.30 kcal/mol, indicating that the ligand can form a relatively stable interaction with the receptor. The gas-phase interaction energy (ΔG_gas) was −60.14 kcal/mol, of which the van der Waals energy (ΔE_vdw) was −41.78 ± 4.60 kcal/mol, representing the most favorable energetic contribution; the electrostatic interaction energy (ΔE_elec) was −18.36 ± 4.10 kcal/mol, which also favored complex formation. These results are consistent with the π–π stacking mediated by Phe820, approximately 57 hydrophobic carbon–carbon contacts, and the continuous hydrophobic enclosure formed by residues including Leu765, Ile768, Ile778, Val782, Ala783, Leu804, Ile813, and Met816 observed in the final-state structure, suggesting that the binding of **18** is primarily driven by van der Waals interactions, aromatic stacking, and hydrophobic complementarity. The hydrogen bonds formed by Gln817 and Tyr612, along with local electrostatic complementarity, mainly contribute to orientational anchoring and conformational constraint.

The solvation energy (ΔG_solv) was +51.01 kcal/mol, exerting a pronounced counteracting effect on ligand binding. Among its components, the polar solvation energy (ΔG_PB) was +55.92 ± 5.80 kcal/mol, representing the largest unfavorable term, indicating that the ligand and the polar groups within the pocket incur a substantial polar desolvation penalty upon transitioning from the aqueous phase to the binding interface. The nonpolar solvation energy (ΔG_SASA) was −4.91 ± 0.45 kcal/mol, reflecting the favorable contribution arising from ligand entry into the hydrophobic pocket and the associated reduction in solvent-accessible surface area. When reclassified according to the underlying mechanism, the net hydrophobic/aromatic contribution (ΔE_vdw + ΔG_SASA) was −46.69 kcal/mol, whereas the net polar contribution (ΔE_elec + ΔG_PB) was +37.56 kcal/mol. Collectively, this system exhibits an energetic profile characterized by hydrophobic enclosure as the dominant driving force for binding, hydrogen bonding and electrostatic interactions responsible for orientational specificity, and a substantial counteracting effect from polar desolvation, which is in good agreement with the proposed polar–hydrophobic composite binding mode. The binding energy between the two was −9.13 kcal/mol.

## 3. Experimental Section

### 3.1. General Information

All reagents and chemicals were obtained from commercial sources and used without further purification unless otherwise noted. ^1^H and ^13^C NMR spectra were recorded on a Bruker 400 MHz spectrometer (Germany) using d_6_-DMSO as the solvent and tetramethylsilane (TMS) as the internal standard. Chemical shifts are reported in parts per million (ppm, δ), and coupling constants (J) are expressed in hertz (Hz). NMR data were processed using MestReNova software (v14). High-resolution electrospray ionization mass spectrometry (HR-ESI-MS) was performed on a Thermo Q Exactive Focus mass spectrometer (Thermo Fisher Scientific, Waltham, MA, USA). HPLC analyses were conducted on an Agilent Technologies 1260 Infinity II system equipped with an Agilent ZORBAX SB-C18 column (5 µm, 4.6 × 250 mm). All compound characterization data are provided in the Supplementary Materials (Figures S1–S76). Molecular docking studies were carried out using MOE 2024 (Molecular Operating Environment) software. Log P values and solubility data for the compounds were obtained using EPI Suite software (v4.1). The primary rat aortic smooth muscle cells used in the experimental section of the manuscript were purchased from Shanghai Fuheng Biotechnology Co., Ltd. (catalog number: FH-R019, China), and the culture medium used was rat aortic smooth muscle cell complete medium, also purchased from the same company (catalog number: PY-R019, China).

### 3.2. General Procedure for Synthesis of Compounds 2-20

*Synthesis of (6R,12aR)-6-(benzo[d][1,3]dioxol-5-yl)-2-(2-hydroxyethyl)-2,3,6,7,12, 12a-hexahydropyrazino [1′,2′:1,6]pyrido [3,4-b]indole-1,4-dione*, **2**

The synthetic procedure for compound **2** was the same as that for tadalafil [20], except that methylamine was replaced with ethanolamine. Compound **2** (478 mg) was obtained in 97.3% yield. ^1^H NMR (400 MHz, DMSO-*d*_6_) δ 11.04 (s, 1H), 7.54 (d, *J* = 8.0 Hz, 1H), 7.31 (d, *J* = 8.0 Hz, 1H), 7.03 (m, 2H), 6.85 (s, 1H), 6.78 (s, 2H), 6.17 (s, 1H), 5.92 (s, 2H), 4.78 (t, *J* = 5.2 Hz, 1H), 4.42 (dd, *J* = 6.8, 4.8 Hz, 1H), 4.27 (d, *J* = 17.2 Hz, 1H), 4.02 (d, *J* = 17.2 Hz, 1H), 3.64–3.44 (m, 4H), 3.37 (s, 1H), 2.97 (dd, *J* = 12, 4 Hz, 1H). ^13^C NMR (101 MHz, DMSO) δ 167.74, 167.18, 147.55, 146.57, 137.28, 136.64, 134.40, 126.24, 121.70, 119.74, 119.34, 118.56, 111.79, 108.46, 107.38, 105.18, 101.37, 58.99, 55.91, 55.48, 51.59, 48.61, 23.22. HR-MS (*m*/*z*): calcd for C_23_H_21_N_3_O_5_ (M + H)^+^, 420.1559; found: 420.1556. Purity (HPLC): 98.85%.

*4-(2-((6R,12aR)-6-(benzo[d][1,3]dioxol-5-yl)-1,4-dioxo-3,4,6,7,12,12a-hexahydro pyrazino [1′,2′:1,6]pyrido [3,4-b]indol-2(1H)-yl)ethoxy)-4-oxobutanoic acid*, **3**

Intermediate I (1 eq, 500 mg) was dissolved in methanol, and ethanolamine (3 eq, 191.1 μL) was added. The reaction mixture was stirred at 70 °C for 5.5 h. After completion of the reaction, the solvent was removed by rotary evaporation, and the residue was precipitated from CH_2_Cl_2_. The precipitate was collected and dried to afford 425 mg of intermediate Ia (yield: 95.4%). Intermediate Ia (1 eq, 300 mg) was dissolved in DMF, and DIPEA (2 eq, 249.5 μL) was added. The mixture was stirred at 55 °C for 1 h. Subsequently, succinic anhydride (1.1 eq, 78.7 mg) was added, and the reaction temperature was raised to 80 °C. The reaction was continued for an additional 5 h. After completion, DMF was removed by rotary evaporation, and the residue was precipitated from CH_2_Cl_2_. The precipitate was collected and dried to yield 322 mg of compound **3** (yield: 86.7%). ^1^H NMR (400 MHz, DMSO-*d*_6_) δ 12.15 (s, 1H), 11.06 (s, 1H), 7.54 (d, *J* = 8.0 Hz, 1H), 7.31 (d, *J* = 8.0 Hz, 1H), 7.03 (m, 2H), 6.85 (s, 1H), 6.77 (s, 2H), 6.18 (s, 1H), 5.92 (s, 2H), 4.43 (dd, *J* = 6.8, 4.8 Hz, 1H), 4.24 (m, 3H), 4.02 (d, *J* = 16.8 Hz, 1H), 3.78 (m, 1H), 3.53 (m, 2H), 2.96 (dd, *J* = 11.6, 4.4 Hz, 1H), 2.46 (m, 4H). ^13^C NMR (101 MHz, DMSO) δ 173.81, 172.46, 167.58 (2C), 147.57, 146.57, 137.22, 136.61, 134.33, 126.21, 121.72, 119.53, 119.36, 118.57, 111.79, 108.45, 107.26, 105.06, 101.38, 61.43, 55.80, 55.35, 51.15, 45.02, 29.18, 29.06, 23.05. HR-MS (*m/z*): calcd for C_27_H_25_N_3_O_8_ (M − H)^−^, 518.1563; found: 518.1559. Purity (HPLC): 99.81%

*5-(2-((6R,12aR)-6-(benzo[d][1,3]dioxol-5-yl)-1,4-dioxo-3,4,6,7,12,12a-hexahydro pyrazino [1′,2′:1,6]pyrido [3,4-b]indol-2(1H)-yl)ethoxy)-5-oxopentanoic acid*, **4**

The synthesis of compound **4** followed the same procedure as that for compound **3**, except that succinic anhydride was replaced with glutaric anhydride, affording 325 mg of compound **4** in 85.2% yield. ^1^H NMR (400 MHz, DMSO-*d*_6_) δ 12.07 (s, 1H), 11.07 (s, 1H), 7.54 (d, *J* = 8.0 Hz, 1H), 7.31 (d, *J* = 8.0 Hz, 1H), 7.03 (m, 2H), 6.85 (s, 1H), 6.77 (s, 2H), 6.17 (s, 1H), 5.92 (s, 2H), 4.43 (dd, *J* = 12.0, 4.8 Hz, 1H), 4.22 (m, 3H), 4.01 (d, *J* = 17.2 Hz, 1H), 3.67 (m, 2H), 3.49 (dd, *J* = 16.0, 4.8 Hz, 1H), 2.96 (dd, *J* = 16.0, 12.0 Hz, 1H), 2.25 (m, 4H), 1.72 (m, 2H). ^13^C NMR (101 MHz, DMSO) δ 174.45, 172.88, 167.61 (2C), 147.58, 146.58, 137.26, 136.62, 134.35, 126.22, 121.71, 119.58, 119.35, 118.60, 111.79, 108.44, 107.25, 105.06, 101.38, 61.30, 55.82, 55.37, 51.03, 44.91, 33.12, 33.09, 23.04, 20.23. HR-MS (*m/z*): calcd for C_28_H_27_N_3_O_8_ (M − H)^−^, 532.1720; found: 532.1722. Purity (HPLC): 98.47%.

*2-((6R,12aR)-6-(benzo[d][1,3]dioxol-5-yl)-1,4-dioxo-3,4,6,7,12,12a-hexahydro pyrazino [1′,2′:1,6]pyrido [3,4-b]indol-2(1H)-yl)acetic acid*, **5**

Intermediate I (1 eq, 500 mg) was dissolved in a DMF/water system (V_DMF_:V_water_ = 2:1), and glycine (3 eq, 263.8 mg) was added. The reaction mixture was stirred at 65 °C for 4 h. After completion of the reaction, the solvent was removed by rotary evaporation, and the residue was precipitated from CH_2_Cl_2_. After drying, 484 mg of compound **5** was obtained as a white solid in 93.3% yield. ^1^H NMR (400 MHz, DMSO-*d*_6_) δ 12.92 (s, 1H), 11.10 (s, 1H), 7.54 (d, *J* = 8.0 Hz, 1H), 7.31 (d, *J* = 8.0 Hz, 1H), 7.03 (m, 2H), 6.87 (s, 1H), 6.78 (m, 2H), 6.20 (s, 1H), 5.92 (s, 2H), 4.50 (dd, *J* = 11.6, 4.8 Hz, 1H), 4.26 (dd, *J* = 30.0, 16.8 Hz, 2H), 4.05 (dd, *J* = 46.0, 17.2 Hz, 2H), 3.48 (dd, *J* = 16.0, 5.2 Hz, 1H), 2.94 (dd, *J* = 15.6, 11.6 Hz, 1H). ^13^C NMR (101 MHz, DMSO) δ 170.45, 168.04, 167.60, 147.60, 146.57, 137.12, 136.59, 134.40, 126.21, 121.72, 119.58, 119.37, 118.58, 111.81, 108.45, 107.25, 104.96, 101.38, 55.61, 55.20, 51.37, 47.50, 22.92. HR-MS (*m/z*): calcd for C_23_H_19_N_3_O_6_ (M + H)^+^, 434.1352; found: 434.1348. Purity (HPLC): 99.67%.

*(2-((1R,3R)-1-(benzo[d][1,3]dioxol-5-yl)-3-(methoxycarbonyl)-1,3,4,9-tetrahydro-2H-pyrido [3,4-b]indol-2-yl)-2-oxoethyl)-D-cysteine*, **6**

Compound **6** was synthesized following the same procedure as compound **5**, with glycine replaced by cysteine, affording 537 mg of compound **6** in 89.6% yield. ^1^H NMR (400 MHz, DMSO-*d*_6_) δ 10.88 (s, 1H), 7.55 (d, *J* = 8.0 Hz, 1H), 7.28 (d, *J* = 8.0 Hz, 1H), 7.07 (m, 2H), 6.81 (m, 2H), 6.64 (m, 1H), 6.47 (dd, *J* = 7.6, 2.8 Hz, 1H), 5.98 (dd, *J* = 7.5, 0.9 Hz, 2H), 5.33 (d, *J* = 6.9 Hz, 1H), 3.98 (d, *J* = 14.8 Hz, 1H), 3.67 (d, *J* = 14.8 Hz, 1H), 3.47 (m, 2H), 3.36 (m, 2H), 3.13 (m, 1H), 3.03 (m, 3H), 2.81 (dd, *J* = 14.0, 8.8 Hz, 1H), 1.26 (d, *J* = 6.8 Hz, 1H). ^13^C NMR (101 MHz, DMSO) δ 171.16, 170.20 (2C), 147.34, 147.01, 136.87, 134.33, 130.63, 126.40, 122.84, 121.97, 119.10, 118.55, 111.69, 109.59, 108.00, 106.94, 101.44, 54.47, 53.07, 52.13, 51.23, 35.51, 35.17, 21.69. HR-MS (*m*/*z*): calcd for C_25_H_25_N_3_O_7_S (M + H)^+^, 512.1492; found: 512.1497. Purity (HPLC): 99.75%

*2-((6R,12aR)-6-(benzo[d][1,3]dioxol-5-yl)-1,4-dioxo-3,4,6,7,12,12a-hexahydro pyrazino [1′,2′:1,6]pyrido [3,4-b]indol-2(1H)-yl)ethane-1-sulfonic acid*, **7**

The synthesis of compound **7** followed the same procedure as that of compound **5**, with glycine replaced by 2-aminoethanesulfonic acid, affording 524 mg of compound **7** in 92.6% yield. ^1^H NMR (400 MHz, DMSO-*d*_6_) δ 11.02 (s, 1H), 7.54 (s, 1H), 7.29 (s, 1H), 7.02 (m, 2H), 6.86 (s, 1H), 6.78 (s, 2H), 6.13 (s, 1H), 5.92 (s, 2H), 4.32 (m, 2H), 3.97 (d, *J* = 17.2 Hz, 1H), 3.78 (m, 1H), 3.50 (m, 2H), 2.95 (t, *J* = 13.2 Hz, 1H). 2.74 (s, 2H). ^13^C NMR (101 MHz, DMSO) δ 167.58, 166.69, 147.51, 146.51, 137.40, 136.66, 134.44, 126.24, 121.66, 119.71, 119.31, 118.54, 111.79, 108.44, 107.38, 105.21, 101.34, 56.03, 55.62, 50.96, 48.79, 43.42, 23.40. HR-MS (*m/z*): calcd for C_23_H_21_N_3_O_7_S (M − H)^−^, 482.1022; found: 482.1018. Purity (HPLC): 99.87%.

*(6R,12aR)-6-(3,4-diethoxyphenyl)-2-methyl-2,3,6,7,12,12a-hexahydropyrazino [1′,2′:1,6]pyrido [3,4-b]indole-1,4-dione*, **8**

Intermediate I (1 eq, 1 g) and 3,4-diethoxybenzaldehyde (1.1 eq, 778 mg) were placed in a reaction flask, acetonitrile was added, and the mixture was refluxed at 80 °C with stirring for 6 h. After completion of the reaction, the mixture was cooled to room temperature, filtered, and the precipitate was washed and dried to afford 1578 mg of intermediate IIb as a white solid. Yield: 92.9%. Intermediate IIb (1 eq, 500 mg) was dissolved in CH_2_Cl_2_ and stirred at 7 °C for 5 min. Triethylamine (3 eq, 483.8 μL) was then added, and the mixture was stirred at 7 °C for an additional 10 min. Chloroacetyl chloride (1.3 eq, 120 μL) was added dropwise to the reaction mixture at 7 °C, and the reaction was completed in approximately 30 min. After completion of the reaction, the solvent was removed by rotary evaporation, and the residue was washed with methanol/water (V_methanol_:V_water_ = 2:1), stirred for 1 h, filtered, and the precipitate was dried to afford intermediate IIIb (518 mg). Yield: 94.8%. Intermediate IIIb (1 eq, 300 mg) was dissolved in a methanol/water system (V_methanol_:V_water_ = 1:1), and 40 vol.% aqueous methylamine solution (3 eq, 246 μL) was added. The mixture was stirred at 70 °C for 6 h. After completion of the reaction, the solvent was removed by rotary evaporation, and the residue was precipitated from CH_2_Cl_2_. The precipitate was dried to afford compound **8** (252 mg). Yield: 92.6%. ^1^H NMR (400 MHz, DMSO-*d*_6_) δ 11.06 (s, 1H), 7.51 (d, *J* = 8.0 Hz, 1H), 7.31 (d, *J* = 8.0 Hz, 1H), 7.06 (m, 2H), 6.87 (m, 3H), 6.59 (d, *J* = 8.3 Hz, 1H), 4.25 (d, *J* = 17.6 Hz, 1H), 3.99 (m, 6H), 3.25 (m, 1H), 2.98 (d, *J* = 13.6 Hz, 1H), 2.85 (s, 3H), 1.30 (m, 6H). ^13^C NMR (101 MHz, DMSO) δ 165.18, 162.67, 148.92, 148.82, 136.75, 131.99, 131.01, 126.39, 122.05, 121.16, 119.29, 118.52, 114.33, 113.80, 111.75, 107.89, 64.56, 64.39, 52.53, 51.37, 51.16, 33.07, 27.22, 15.16 (2C). HR-MS (*m/z*): calcd for C_25_H_27_N_3_O_4_ (M − H)^−^, 432.1923; found: 432.1919. Purity (HPLC): 97.41%.

*(6R,12aR)-6-(3,4-diethoxyphenyl)-2-(2-hydroxyethyl)-2,3,6,7,12,12a-hexahydro pyrazino [1′,2′:1,6]pyrido [3,4-b]indole-1,4-dione*, **9**

Compound **9** was synthesized following the same procedure as compound **8**, except that aqueous methylamine solution was replaced with ethanolamine, affording compound **9** (264 mg, 89.4% yield). ^1^H NMR (400 MHz, DMSO-*d*_6_) δ 11.06 (s, 1H), 7.52 (d, *J* = 8.0 Hz, 1H), 7.31 (d, *J* = 8.0 Hz, 1H), 7.06 (m, 2H), 6.91 (d, *J* = 8.4 Hz, 1H), 6.85 (s, 2H), 6.61 (d, *J* = 8.4 Hz, 1H), 4.80 (t, *J* = 6.0 Hz, 1H), 4.32 (d, *J* = 17.6 Hz, 1H), 4.11 (m, 2H), 3.97 (m, 4H), 3.56 (m, 2H), 3.39 (m, 2H), 3.26 (d, *J* = 4.4 Hz, 1H), 2.99 (dd, *J* = 12, 3.6 Hz, 1H), 1.29 (q, *J* = 6.8 Hz, 6H). ^13^C NMR (101 MHz, DMSO) δ 165.27, 162.86, 148.94, 148.79, 136.74, 131.92, 131.05, 126.39, 122.05, 121.21, 119.31, 118.52, 114.34, 113.78, 111.75, 107.85, 64.55, 64.38, 58.56, 52.64, 51.40, 50.69, 48.44, 27.20, 15.17(2C). HR-MS (*m/z*): calcd for C_26_H_29_N_3_O_5_ (M + H)^+^, 464.2186; found: 464.2184. Purity (HPLC): 98.81%

*4-(2-((6R,12aR)-6-(3,4-diethoxyphenyl)-1,4-dioxo-3,4,6,7,12,12a-hexahydropyrazino [1′,2′:1,6]pyrido [3,4-b]indol-2(1H)-yl)ethoxy)-4-oxobutanoic acid*, **10**

Intermediate I (1 eq, 1 g) and 3,4-diethoxybenzaldehyde (1.1 eq, 778 mg) were placed in a reaction flask, acetonitrile was added, and the mixture was refluxed at 80 °C with stirring for 6 h. After completion of the reaction, the mixture was cooled to room temperature, filtered, and the precipitate was washed and dried to afford 1578 mg of intermediate IIb as a white solid. Yield: 92.9%. Intermediate IIc (1 eq, 500 mg) was dissolved in CH_2_Cl_2_ and stirred at 7 °C for 5 min. Triethylamine (3 eq, 483.8 μL) was then added, and the mixture was stirred at the same temperature for 10 min. Chloroacetyl chloride (1.3 eq, 120 μL) was added dropwise at 7 °C, and the reaction was completed in approximately 30 min. After completion, the solvent was removed by rotary evaporation, and the residue was washed with methanol/water (V_methanol_:V_water_ = 2:1), stirred for 1 h, filtered, and the precipitate was dried to afford intermediate IIIc (518 mg, 94.8%). Intermediate IIIc (1 eq, 300 mg) was dissolved in a methanol/water system (Vmethanol/Vwater = 2:1). Ethanolamine (3 eq, 115 μL) was added, and the mixture was stirred at 70 °C for 6 h. After completion of the reaction, the solvent was removed by rotary evaporation, and the residue was precipitated from CH_2_Cl_2_. The precipitate was dried to afford intermediate IVc (264 mg, 89.4% yield). Intermediate IVc (1 eq, 200 mg) was dissolved in DMF, and DIPEA (2 eq) was added. The mixture was stirred at 55 °C for 1 h, after which succinic anhydride (1 eq, 43.2 mg) was added, and the reaction was stirred at 70 °C for 6 h. After completion, the solvent was removed by rotary evaporation, and the residue was precipitated from CH_2_Cl_2_. The precipitate was dried to afford 218 mg of compound **10**. Yield: 89.7%. ^1^H NMR (400 MHz, DMSO-*d*_6_) δ 12.16 (s, 1H), 11.06 (s, 1H), 7.51 (d, *J* = 8.0 Hz, 1H), 7.32 (d, *J* = 8.0 Hz, 1H), 7.06 (m, 2H), 6.91 (d, *J* = 8.4 Hz, 1H), 6.84 (d, *J* = 4.8 Hz, 2H), 6.60 (d, *J* = 8.4 Hz, 1H), 4.33 (d, *J* = 17.6 Hz, 1H), 4.20 (s, 2H), 4.10 (m, 2H), 3.96 (m, 4H), 3.40 (m, 2H), 3.24 (m, 1H), 2.97 (m, 1H), 2.48 (m, 4H), 1.29 (q, *J* = 6.8 Hz, 6H). ^13^C NMR (101 MHz, DMSO) δ 173.78, 172.54, 165.69, 162.78, 148.92, 148.81, 136.72, 131.84, 131.05, 126.39, 122.08, 121.14, 119.33, 118.54, 114.24, 113.76, 111.76, 107.82, 64.54, 64.37, 61.16, 52.69, 51.41, 50.15, 44.91, 29.22, 29.10, 26.98, 15.16 (2C). HR-MS (*m/z*): calcd for C_30_H_33_N_3_O_8_ (M − H)^−^, 562.2819; found: 562.2819. Purity (HPLC): 96.23%.

*(6R,12aR)-6-(3-ethoxy-4-hydroxyphenyl)-2-methyl-2,3,6,7,12,12a-hexahydro pyrazino [1′,2′:1,6]pyrido [3,4-b]indole-1,4-dione*, **11**

Compound **11** was synthesized following the same procedure as compound **8**, except that 3,4-diethoxybenzaldehyde was replaced with 3-hydroxy-4-ethoxybenzaldehyde, affording compound **11** (248 mg, 90.3% yield). ^1^H NMR (400 MHz, DMSO-*d*_6_) δ 11.06 (s, 1H), 9.05 (s, 1H), 7.50 (d, *J* = 8.0 Hz, 1H), 7.31 (d, *J* = 8.0 Hz, 1H), 7.06 (m, 2H), 6.81 (d, *J* = 6.8 Hz 2H), 6.74 (d, *J* = 8.0 Hz, 1H), 6.50 (m, 1H), 4.24 (d, *J* = 18.0 Hz, 1H), 4.07 (m, 2H), 3.92 (m, 2H), 3.26 (dd, *J* = 15.2, 4.0 Hz, 1H), 2.95 (m, 1H), 2.85 (s, 3H), 1.28 (t, *J* = 6.8 Hz, 3H). ^13^C NMR (101 MHz, DMSO) δ 165.22, 162.59, 147.67, 147.28, 136.71, 131.21, 130.35, 126.39, 121.99, 121.39, 119.25, 118.48, 115.82, 114.51, 111.74, 107.77, 64.62, 52.43, 51.41, 51.15, 33.06, 27.23, 15.17. HR-MS (*m/z*): calcd for C_23_H_23_N_3_O_4_ (M + H)^+^, 406.1767; found: 406.1763. Purity (HPLC): 97.55%.

*(6R,12aR)-6-(3-ethoxy-4-hydroxyphenyl)-2-(2-hydroxyethyl)-2,3,6,7,12,12a-hexahydropyrazino [1′,2′:1,6]pyrido [3,4-b]indole-1,4-dione*, **12**

Compound **12** was synthesized following the same procedure as compound **11**, except that aqueous methylamine solution was replaced with ethanolamine, affording compound **12** (275 mg, 93.2% yield). ^1^H NMR (400 MHz, DMSO-*d*_6_) δ 11.04 (s, 1H), 9.03 (s, 1H), 7.51 (d, *J* = 8.0 Hz, 1H), 7.31 (d, *J* = 8.0 Hz, 1H), 7.06 (m, 2H), 6.78 (m, 3H), 6.52 (d, *J* = 8.0 Hz, 1H), 4.79 (s, 1H), 4.31 (d, *J* = 17.6 Hz, 1H), 4.11 (m, 2H), 3.93 (m, 2H), 3.56 (s, 2H), 3.38 (m, 2H), 3.26 (m, 1H), 2.98 (m, 1H), 1.28 (s, 3H). ^13^C NMR (101 MHz, DMSO) δ 165.32, 162.78, 147.70, 147.28, 136.73, 131.26, 130.31, 126.41, 122.00, 121.47, 119.28, 118.49, 115.86, 114.57, 111.74, 107.75, 64.64, 58.57, 52.56, 51.46, 50.70, 48.44, 27.22, 15.17. HR-MS (*m/z*): calcd for C_24_H_25_N_3_O_5_ (M − H)^−^, 434.1716; found: 434.1715. Purity (HPLC): 96.22%.

*4-(2-((6R,12aR)-6-(3-ethoxy-4-hydroxyphenyl)-1,4-dioxo-3,4,6,7,12,12a-hexahydro pyrazino [1′,2′:1,6]pyrido [3,4-b]indol-2(1H)-yl)ethoxy)-4-oxobutanoic acid*, **13**

Compound **13** was synthesized following the same procedure as compound **10**, except that 3,4-diethoxybenzaldehyde was replaced with 3-hydroxy-4-ethoxybenzaldehyde, affording compound **13** (226 mg, 91.9% yield). ^1^H NMR (400 MHz, DMSO-*d*_6_) δ 12.19 (s, 1H), 11.05 (s, 1H), 9.04 (s, 1H), 7.51 (d, *J* = 8.0 Hz, 1H), 7.31 (d, *J* = 8.0 Hz, 1H), 7.06 (m, 2H), 6.77 (m, 3H), 6.51 (d, *J* = 8.4 Hz,1H), 4.33 (d, *J* = 17.6 Hz, 1H), 4.21 (t, *J* = 5.6 Hz, 2H), 4.11 (m, 2H), 3.92 (m, 2H), 3.39 (m, 2H), 3.22 (m, 1H), 2.96 (t, *J* = 12 Hz, 1H), 2.46 (m, 4H) 1.28 (t, *J* = 6.8 Hz, 3H). ^13^C NMR (101 MHz, DMSO) δ 173.79, 172.53, 165.74, 162.70, 147.67, 147.30, 136.69, 131.26, 130.22, 126.40, 122.01, 121.39, 119.28, 118.50, 115.83, 114.45, 111.74, 107.71, 64.61, 61.18, 52.60, 51.46, 50.18, 44.91, 29.22, 29.10, 27.00, 15.16. HR-MS (*m/z*): calcd for C_28_H_29_N_3_O_8_ (M − H)^−^, 534.1876; found: 534.1872. Purity (HPLC): 97.08%.

*5-(2-((6R,12aR)-6-(3-ethoxy-4-hydroxyphenyl)-1,4-dioxo-3,4,6,7,12,12a-hexahydro pyrazino [1′,2′:1,6]pyrido [3,4-b]indol-2(1H)-yl)ethoxy)-5-oxopentanoic acid*, **14**

Compound **14** was synthesized following the same procedure as compound **13**, except that succinic anhydride was replaced with glutaric anhydride, affording compound **14** (236 mg, 93.5% yield). ^1^H NMR (400 MHz, DMSO-*d*_6_) δ 12.05 (m, 2H), 11.12 (s, 1H), 7.53 (d, *J* = 8.0 Hz, 1H), 7.33 (d, *J* = 8.0 Hz, 1H), 7.04 (m, 4H), 6.89 (s, 1H), 6.69 (d, *J* = 8.0, 1H), 4.35 (d, *J* = 17.6 Hz, 0H), 4.21 (s, 2H), 4.12 (m, 2H), 3.94 (m, 2H), 3.26 (m, 1H), 2.98 (t, *J* = 12.0 Hz, 1H), 2.59 (t, *J* = 7.2 Hz, 2H). 2.24 (t, *J* = 7.2 Hz, 1H), 1.85 (t, *J* = 7.2 Hz, 1H), 1.71 (t, *J* = 7.2 Hz, 1H), 1.25 (t, *J* = 6.8 Hz, 1H). ^13^C NMR (101 MHz, DMSO) δ 174.37, 172.97, 171.18, 165.54, 162.96, 150.71, 140.04, 138.19, 136.78, 130.56, 126.37, 123.22, 120.74, 119.37, 118.62, 114.01, 111.80, 108.02, 64.56, 60.87, 52.90, 51.49, 49.98, 44.86, 33.12, 32.95, 20.56, 20.26, 14.87. HR-MS (*m/z*): calcd for C_29_H_31_N_3_O_8_ (M − H)^−^, 548.2033; found: 548.2033. Purity (HPLC): 97.00%.

*(6R,12aR)-6-(4-ethoxy-3-methoxyphenyl)-2-methyl-2,3,6,7,12,12a-hexahydropyrazi no [1′,2′:1,6]pyrido [3,4-b]indole-1,4-dione*, **15**

Compound **15** was synthesized following the same procedure as compound **7**, except that 3,4-diethoxybenzaldehyde was replaced with 3-methoxy-4-ethoxybenzaldehyde, affording compound **15** (255 mg, 92.6% yield). ^1^H NMR (400 MHz, DMSO-*d*_6_) δ 11.05 (s, 1H), 7.51 (d, *J* = 8.0 Hz, 1H), 7.32 (d, *J* = 8.0 Hz, 1H), 7.06 (m, 2H), 6.88 (m, 3H), 6.58 (d, *J* = 7.6 Hz, 1H), 4.25 (d, *J* = 18.0 Hz, 1H), 4.07 (m, 1H), 3.97 (m, 2H), 3.69 (s, 3H), 3.28 (m, 1H), 2.97 (m, 1H), 2.86 (s, 3H), 1.30 (t, *J* = 6.8 Hz, 3H). ^13^C NMR (101 MHz, DMSO) δ 165.19, 162.72, 149.58, 148.64, 136.74, 131.90, 131.02, 126.39, 122.06, 120.99, 119.30, 118.52, 113.23, 112.66, 111.76, 107.89, 64.29, 56.05, 52.53, 51.41, 51.15, 33.07, 27.22, 15.16. HR-MS (*m/z*): calcd for C_24_H_25_N_3_O_4_ (M − H)^−^, 418.1767; found: 418.1769. Purity (HPLC): 96.92%.

*(6R,12aR)-6-(4-ethoxy-3-methoxyphenyl)-2-(2-hydroxyethyl)-2,3,6,7,12,12a-hexahydropyrazino [1′,2′:1,6]pyrido [3,4-b]indole-1,4-dione*, **16**

Compound **16** was synthesized following the same procedure as compound **15**, except that aqueous methylamine solution was replaced with ethanolamine, affording compound **16** (243 mg, 89.4% yield). ^1^H NMR (400 MHz, DMSO-*d*_6_) δ 11.06 (s, 1H), 7.52 (d, *J* = 8.0 Hz, 1H), 7.31 (d, *J* = 8.0 Hz, 1H), 7.05 (m, 2H), 6.87 (m, 3H), 6.59 (m, 1H), 4.81 (s, 1H), 4.32 (d, *J* = 17.6 Hz, 1H), 4.14 (m, 2H), 3.97 (m, 2H), 3.69 (s, 3H), 3.56 (m, 2H), 3.38 (m, 2H), 3.38 (m, 1H), 2.99 (t, *J* = 14.0 Hz, 1H), 1.30 (t, *J* = 6.8 Hz, 3H). ^13^C NMR (101 MHz, DMSO) δ 165.28, 162.90, 149.57, 148.66, 136.75, 131.84, 131.07, 126.40, 122.06, 121.06, 119.31, 118.53, 113.25, 112.70, 111.76, 107.86, 64.29, 58.55, 56.06, 52.65, 51.45, 50.69, 48.44, 27.20, 15.17. HR-MS (*m/z*): calcd for C_25_H_27_N_3_O_5_ (M − H)^−^, 448.1873; found: 448.1872. Purity (HPLC): 97.84%.

*(6R,12aR)-2-(2-hydroxyethyl)-6-(4-methoxyphenyl)-2,3,6,7,12,12a-hexahydro pyrazino [1′,2′:1,6]pyrido [3,4-b]indole-1,4-dione*, **17**

Compound **17** was synthesized following the same procedure as compound **9**, except that aqueous methylamine was replaced with ethanolamine, affording compound **17** (274 mg, 93.0% yield). ^1^H NMR (400 MHz, DMSO-*d*_6_) δ 11.06 (s, 1H), 7.52 (d, *J* = 8.0 Hz, 1H), 7.31 (d, *J* = 8.0 Hz, 1H), 7.12 (m, 3H), 7.02 (t, *J* = 7.6 Hz, 1H), 6.91 (m, 3H), 4.79 (s, 1H), 4.33 (d, *J* = 17.0 Hz, 2H), 4.08 (m, 2H), 3.73 (s, 3H), 3.54 (m, 2H), 3.38 (s, 2H), 3.28 (m, 1H), 3.00 (m, 1H). ^13^C NMR (101 MHz, DMSO) δ 165.24, 162.84, 159.70, 136.74, 131.55, 131.14, 129.91 (2C), 126.44, 122.05, 119.33, 118.51, 114.56 (2C), 111.76, 107.81, 58.57, 55.67, 52.54, 51.09, 50.69, 48.43, 27.17. HR-MS (*m/z*): calcd for C_23_H_23_N_3_O_4_ (M − H)^−^, 404.1610; found: 404.1608. Purity (HPLC): 98.14%.

*(6R,12aR)-6-(4-methoxyphenyl)-2-methyl-2,3,6,7,12,12a-hexahydropyrazino [1′,2′:1,6]pyrido [3,4-b]indole-1,4-dione*, **18**

Compound **18** was synthesized following the same procedure as compound **8**, except that 3,4-diethoxybenzaldehyde was replaced with p-methoxybenzaldehyde, affording compound **18** (256 mg, 93.8% yield). ^1^H NMR (400 MHz, DMSO-*d*_6_) δ 11.06 (s, 1H), 7.51 (d, *J* = 8.0 Hz, 1H), 7.32 (d, *J* = 8.0 Hz, 1H), 7.12 (m, 3H), 7.03 (d, *J* = 7.6 Hz, 1H), 6.91 (m, 3H), 4.26 (d, *J* = 17.0 Hz, 1H), 4.04 (m, 2H), 3.73 (s, 3H), 3.25 (m, 1H), 2.99 (d, *J* = 13.6 Hz, 1H), 2.85 (s, 3H). ^13^C NMR (101 MHz, DMSO) δ 165.15, 162.66, 159.68, 136.74, 131.59, 131.10, 129.83 (2C), 126.43, 122.05, 119.32, 118.50, 114.53 (2C), 111.76, 107.83, 55.67, 52.44, 51.16, 51.05, 33.07, 27.18. HR-MS (*m/z*): calcd for C_22_H_21_N_3_O_3_ (M − H)^−^, 374.1505; found: 374.1504. Purity (HPLC): 98.20%.

*4-(2-((6R,12aR)-6-(4-methoxyphenyl)-1,4-dioxo-3,4,6,7,12,12a-hexahydropyrazino [1′,2′:1,6]pyrido [3,4-b]indol-2(1H)-yl)ethoxy)-4-oxobutanoic acid*, **19**

Compound **19** was synthesized following the same procedure as compound **10**, except that 3,4-diethoxybenzaldehyde was replaced with p-methoxybenzaldehyde, affording compound **19** (228 mg, 91.4% yield). ^1^H NMR (400 MHz, DMSO-*d*_6_) δ 12.21 (s, 1H), 11.07 (s, 1H), 7.52 (d, *J* = 8.0 Hz, 1H), 7.32 (d, *J* = 8.0 Hz, 1H), 7.12 (m, 3H), 7.02 (t, *J* = 7.6 Hz, 1H), 6.91 (m, 3H), 4.34 (d, *J* = 17.6 Hz, 1H), 4.11 (m, 4H), 3.73 (m, 4H), 3.39 (m, 2H), 3.23 (s, 1H), 2.98 (t, *J* = 13.6 Hz, 1H), 2.47 (m, 4H), 1.24 (m, 1H). ^13^C NMR (101 MHz, DMSO) δ 173.79, 172.54, 165.66, 162.78, 159.68, 136.71, 131.45, 131.13, 129.83 (2C), 126.43, 122.08, 119.35, 118.53, 114.55 (2C), 111.76, 107.77, 61.14, 55.66, 52.60, 51.09, 50.15, 44.90, 29.21, 29.09, 26.94. HR-MS (*m/z*): calcd for C_27_H_27_N_3_O_7_ (M − H)^−^, 504.1771; found: 504.1767. Purity (HPLC): 97.16%.

*(6R,12aR)-6-(3-fluoro-4-methoxyphenyl)-2-methyl-2,3,6,7,12,12a-hexahydropyrazi no [1′,2′:1,6]pyrido [3,4-b]indole-1,4-dione*, **20**

Intermediate I (1 eq, 1 g) and 3-fluoro-4-methoxybenzaldehyde (1.1 eq, 778 mg) were placed in a reaction flask, acetonitrile was added, and the mixture was stirred under reflux at 80 °C for 6 h. After completion of the reaction, the mixture was cooled to room temperature, filtered, and the precipitate was washed and dried to afford intermediate IId as a white solid (1578 mg, yield: 92.9%). Intermediate IId (1 eq, 500 mg) was dissolved in CH_2_Cl_2_ and stirred at 7 °C for 5 min. Triethylamine (3 eq, 515 μL) was then added, and the mixture was stirred at 7 °C for an additional 10 min. Chloroacetyl chloride (1.3 eq, 128 μL) was added dropwise at 7 °C, and the reaction was complete in approximately 30 min. After completion of the reaction, the solvent was removed by rotary evaporation. The residue was washed with methanol/water (V_methanol_:V_water_ = 2:1), stirred for 1 h, filtered, and the precipitate was dried to afford intermediate IIId (518 mg, yield: 94.8%). Intermediate IIId (1 eq, 300 mg) was dissolved in a methanol/water system (Vmethanol:Vwater = 2:1). A 40 vol.% aqueous methylamine solution (3 eq, 246 μL) was added, and the mixture was stirred at 70 °C for 6 h. After completion of the reaction, the solvent was removed by rotary evaporation, and the residue was precipitated from CH_2_Cl_2_. The precipitate was dried to afford compound **20** (252 mg, yield: 92.0%). ^1^H NMR (400 MHz, DMSO-*d*_6_) δ 11.01 (s, 1H), 7.55 (d, *J* = 8.0 Hz, 1H), 7.30 (d, *J* = 8.0 Hz, 1H), 7.16 (dd, *J* = 12.5, 1.8 Hz, 1H), 7.06 (m, 4H), 6.15 (s, 1H), 4.40 (dd, *J* = 7.6, 4.4 Hz, 1H), 4.17 (dd, *J* = 17.2 Hz, 1H), 3.95 (d, *J* = 17.2 Hz, 1H), 3.75 (s, 3H), 3.54 (dd, *J* = 11.2, 4.4 Hz, 1H), 3.00 (m, 1H), 2.93 (s, 3H). ^13^C NMR (101 MHz, DMSO) δ 167.38, 166.92, 152.71, 150.29, 146.48, 146.38, 136.75, 136.48, 136.43, 134.07, 126.24, 122.70, 122.67, 121.79, 119.38, 118.62, 114.74, 114.55, 114.31, 111.81, 105.44, 56.51, 56.02, 55.31, 51.91, 33.35, 23.72. HR-MS (*m/z*): calcd for C_22_H_20_FN_3_O_3_ (M − H)^−^, 392.1410; found: 392.1409. Purity (HPLC): 98.96%.

### 3.3. Cell Cytotoxicity Assay (CCK-8)

Primary rat aortic smooth muscle cells in the exponential growth phase were harvested. The culture medium was removed, and the cells were rinsed with PBS, detached using trypsin, and counted. The cell suspension was adjusted to a density of 2.5 × 10^4^ cells/mL, and 100 μL was seeded into each well of a 96-well plate. The plate was then incubated overnight in a humidified incubator (5% CO_2_, 37 °C). After cell attachment, the tadalafil derivative stock solution was diluted with culture medium to the desired concentrations (initial concentration: 100 μM) and added to the plate using a serial 1.5-fold dilution scheme. Three replicate wells were prepared for each concentration, along with a control group. Cells were then incubated for 72 h. Following drug exposure, 10 μL of CCK-8 solution was added to each well, and the plate was incubated for an additional 2–4 h. Optical density (OD) was recorded at 450 nm using a microplate reader, and the data were analyzed with GraphPad Prism 9 to determine IC_50_ values. The growth inhibition rate was calculated as follows:

Growth inhibition rate (%) = (OD_negative control − OD_test  compound)/(OD_negative control − OD_background) × 100%.

### 3.4. Nitric Oxide (NO) Assay Kit Detection

Samples with high protein content, such as serum or culture medium containing high serum levels, may form precipitates upon addition of Griess Reagent I. A preliminary test can be performed by mixing 50 μL of sample with 50 μL of Griess Reagent I to check for precipitation. If precipitation occurs, the sample may be heated in a boiling water bath for 5 min to denature proteins, followed by centrifugation at 12,000× *g* for 5 min. The supernatant is then collected for analysis. For cell or tissue samples, lysis can be performed using the Cell and Tissue Lysis Buffer for Nitric Oxide Assay (S3090, Beyotime, Shanghai, China). Urine samples generally require dilution with water (10–50-fold) prior to detection. Heparin-anticoagulated plasma should not be used, as it tends to form precipitates upon reaction with Griess Reagent. The 1 M NaNO_2_ stock solution should be diluted using the same buffer or medium used for sample preparation to final concentrations of 2, 5, 10, 20, 40, 60, and 80 μmol/L. Approximately 1 mL of double-distilled or Milli-Q-grade water was added to 5 mg of NADPH, mixed by inversion until fully dissolved, and then brought to a final volume of 3 mL with the same water to obtain a 2 mM NADPH solution. FAD was supplied in solution form and may be aliquoted and stored at −20 °C. Nitrate Reductase and LDH should be removed from storage immediately before use and kept on ice during handling. All other kit reagents should be kept on ice after dissolution. Griess Reagent I and II should be equilibrated to room temperature prior to use. Nitric oxide concentrations in samples were calculated based on the standard curve.

### 3.5. PDEs Enzymatic Assays

Complete PDE Assay Buffer was prepared by adding 20 μL of 0.5 M DTT to 10 mL of PDE Assay Buffer. PDE5A1 was thawed on ice, and the reagent tube was centrifuged. PDE5A1 was then diluted to 5 pg/mL with PDE Assay Buffer. Forty microliters of the diluted PDE5A1 was added to the positive control and test inhibitor wells. Forty-five microliters of PDE Assay Buffer was added to the blank wells, and 40 μL to the reference control wells. Preparation of test inhibitors: For titration experiments, a series of 10-fold serial dilutions was prepared. Five microliters of each inhibitor dilution was added to the “test inhibitor” wells, while 5 μL of diluent was added to the “blank,” “reference control,” and “positive control” wells. One tube of FAM-Cyclic-3′,5′-GMP was dissolved in 400 μL of complete PDE Assay Buffer to prepare a 3 μM solution. Five microliters of this substrate was added to the “reference control,” “positive control,” and “test inhibitor” wells to initiate the reaction, while 5 μL of complete PDE Assay Buffer was added to the “blank” wells. The plate was incubated at room temperature for 1 h. The PDE Binding Agent tube was gently mixed and diluted 100-fold with Binding Agent Diluent (cGMP) (100 μL/well). One hundred microliters of the diluted binding agent was then added to each well, and the plate was incubated at room temperature with gentle shaking for 30 min. Fluorescence polarization (FP) was measured using a fluorescence plate reader capable of FP detection (excitation wavelength 470 nm, bandwidth 5 nm; emission wavelength λ_em = 528 nm, bandwidth 10 nm), with the instrument set to FP mode. Blank values were subtracted from all other readings.

### 3.6. cGMP Assays

Rat aortic smooth muscle cells were seeded into 6-well plates at a density of 1 × 10^6^ cells per well and incubated overnight. The cells were divided into three groups: control, tadalafil, and compound 18. Each group was treated with the respective agent at a concentration of 10 μM for 0.5 h, followed by stimulation of particulate guanylate cyclase with atrial natriuretic factor (0.1 μmol/L) at 37 °C for 10 min. The cell culture supernatant from each group was collected and retained. The cells were then harvested by digestion and subjected to four freeze–thaw cycles in liquid nitrogen, followed by centrifugation at 14,000 rpm for 20 min, and the supernatant was collected. cGMP concentrations in both the cell culture supernatant and cell lysates were determined using a rat cGMP ELISA kit (ml003133, Shanghai Enzyme-linked Biotechnology Co., Ltd., Shanghai, China) according to the manufacturer’s instructions.

### 3.7. Molecular Dynamics Simulation System and Methods

#### 3.7.1. System Construction

PDE5 coordinates were obtained from PDB entry 1XOZ. Protein topology was generated with GROMACS pdb2gmx. Molecular dynamics simulations were performed with GROMACS (production run, version 2026.3). The topology was generated using GROMACS pdb2gmx. The AMBER99SB-ILDN force field was used for the protein, and the SPC water model was employed. The ligand 18H was incorporated into the topology as an independent residue/molecular group, including custom atom types and charge parameters. After solvation and ion neutralization, a protein–ligand–water–ion complex was formed, serving as the input for subsequent minimization and dynamics simulations. Index groups included Protein, Backbone, C-alpha, 18H, Protein_18H, Water, Water_and_ions, and System. Compound **18** was docked into the catalytic pocket, and the initial simulation structure was the prepared complex pose that preserved key tadalafil-like contacts (Gln817 polar anchoring, Tyr612 auxiliary polar contact, Phe820 aromatic packing, and hydrophobic lining residues). Formal redocking RMSD validation was not performed and is noted as a limitation. The solvated and neutralized system (17,684 SPC waters and 9 Na^+^ ions) was simulated with periodic boundary conditions (pbc = xyz) for 100 ns under the NPT ensemble at 300 K and 1 bar using GROMACS 2026.3.

#### 3.7.2. Simulation Protocol and Parameters

The simulation protocol followed standard protein–ligand MD procedures: (1) energy minimization to remove initial steric clashes; (2) NVT heating/temperature equilibration; (3) NPT pressure equilibration; and (4) 100 ns unrestrained production dynamics. Key parameters for the production phase were as follows: integration time step dt = 2 fs, total number of steps 50,000,000 (corresponding to 100 ns); bond constraints were applied using LINCS (h-bonds); electrostatic interactions were calculated using the Particle Mesh Ewald (PME) method, with a cutoff of 1.4 nm for both short-range electrostatics and van der Waals interactions; temperature coupling was performed using the V-rescale thermostat, with coupling groups of Protein_18H and Water_and_ions, and a reference temperature of 300 K; pressure coupling was performed using the Parrinello–Rahman isotropic barostat with a reference pressure of 1.0 bar; trajectory and energy output intervals corresponded to 100 ps sampling (trajectories processed with periodic boundary conditions removed and centered on the Protein were used for analysis). Production MD (100 ns) was carried out in the NPT ensemble at 300 K and 1.0 bar. Ligand bonded parameters and partial charges were generated with ACPYPE v2023.10.27 using the GAFF force field and AM1-BCC charges, and then combined with AMBER99SB-ILDN for the protein in GROMACS. Trajectory analyses were performed with GROMACS utilities (gmx trjconv, gmx energy, gmx rms, gmx rmsf, gmx gyrate, gmx hbond, gmx sasa, gmx mindist, gmx covar/anaeig). Binding free energies were estimated by MM/PBSA.

#### 3.7.3. Analysis Metrics

Based on the processed trajectories, the following quantities were calculated: potential energy/total energy, temperature, pressure, density; RMSD of the backbone, Cα, protein, and protein–ligand complex; residue-level Cα/backbone RMSF; radius of gyration (Rg) of the protein and complex; number of intra-protein hydrogen bonds and protein–water hydrogen bonds; solvent-accessible surface area (SASA) of the protein and complex; minimum distance between protein and ligand and number of contacting atom pairs; and eigendecomposition of the backbone covariance matrix with two-dimensional projection onto PC1–PC2. Unless otherwise stated, time-series statistics are reported as mean ± standard deviation over the last 50 ns. 

## 4. Conclusions and Perspectives

In the present study, a focused library of tadalafil analogues was designed and synthesized through two complementary modification strategies: extension of the amide nitrogen with flexible carbon spacers bearing terminal carboxylic acids, and replacement of the 3,4-methylenedioxybenzyl moiety with substituted aromatic aldehydes. The synthesized compounds were systematically profiled with respect to cytotoxicity, calculated lipophilicity and aqueous solubility, NO-releasing capacity, and direct PDE5A enzymatic inhibition. Structure–activity analysis indicated that simply elongating the carbon chain at the amide nitrogen improves predicted docking affinity toward PDE5 but fails to translate into enhanced NO release or enzyme inhibition, implying that substituents at this position do not engage the key functional residues within the catalytic pocket. By contrast, replacing the methylenedioxy ring with a p-methoxyphenyl group proved decisive: compound **18**, which combines this aromatic substitution with an *N*-methyl amide, produced the strongest NO-releasing response among all analogues and suppressed residual PDE5A activity to approximately 39% at 10 μM, while maintaining a favorable logP (1.53) and a low cytotoxicity under the tested in vitro conditions comparable to that of the parent drug.

Molecular dynamics simulations over 100 ns further rationalized the behavior of compound **18** within the PDE5 binding site. The ligand remained stably resident throughout the simulation, as evidenced by plateaued RMSD values, a conserved radius of gyration, and a persistent protein–ligand minimum distance of ~0.23 nm. The binding mode is best described as “hydrogen-bond-directed anchoring combined with aromatic and hydrophobic envelopment”: Gln817 and Tyr612 provide the polar lock, Phe820 clamps the fused bicyclic core through π–π stacking (~3.78 Å), and eight hydrophobic residues line the pocket with nearly 57 carbon–carbon contacts. MM/PBSA decomposition attributed the binding free energy of −9.13 ± 3.30 kcal/mol predominantly to van der Waals and nonpolar solvation terms, confirming that hydrophobic complementarity, rather than polar interactions, is the principal driving force for recognition of this analogue by PDE5A.

Protein and complex SASA remained stable over the final 50 ns (159.48 ± 2.21 and 156.88 ± 2.22 nm^2^, respectively). Because the numerical difference is within the observed fluctuation range, SASA is used here only as a convergence check of the solvation interface, not as evidence of ligand-induced conformational change. Global RMSD and RMSF likewise indicate an intact folded receptor without large-scale remodeling. Ligand residence is instead supported by the persistent protein–ligand minimum distance (0.229 ± 0.008 nm), sustained interfacial contacts, the final binding mode, and MM/PBSA binding free-energy analysis (ΔG_bind = −9.13 ± 3.30 kcal/mol).

Several limitations of the current work should be acknowledged. The biological evaluation was restricted to in vitro enzyme and cellular assays, and the selectivity profile of compound **18** against PDE1, PDE6, and PDE11—central to mitigating the adverse effects of the parent scaffold—remains to be determined. Future efforts will therefore focus on (i) a full isoform selectivity panel to verify whether the p-methoxybenzyl substitution reshapes the selectivity window; (ii) ex vivo cavernosal smooth muscle relaxation and in vivo efficacy studies in ED animal models to confirm the functional relevance of the enhanced NO release; (iii) pharmacokinetic characterization, including solubility-limited absorption and first-pass metabolism, given that the solubility advantage predicted here has yet to be validated experimentally; and (iv) structure-based exploration of the adjacent subpockets revealed by the MD trajectories, which may accommodate additional polar or ionizable groups to balance potency, selectivity, and developability. Taken together, this work refines the SAR landscape of the tadalafil scaffold and delivers compound **18** as a rationally supported lead for the development of next-generation PDE5 inhibitors with improved druggability.

## Figures and Tables

**Table 1:** Lipid–water partition coefficients, solubility, and molecular docking data of tadalafil derivatives.

Compounds	IC_50_ (RASMC Activity, μM) ^a^	Log P	Docking Score	Water Solubility Estimate from Log Kow (WSKOW v1.42), mg/L	Water Solubility Estimate from Fragments: Wat Sol (v1.01 est)
Tadalafil	-	2.36	−9.6493	217.7	9.09
**2**	-	2.21	−9.9722	961.6	166.12
**3**	-	1.70	−11.0793	50.84	10.31
**4**	-	2.21	−11.1087	15.71	3.07
**5**	1.29 ± 0.14	0.69	−10.9139	220.1	93.30
**6**	8.19 ± 0.32	0.46	−10.3934	2.48	0.55
**7**	-	−0.56	−10.3468	578.6	14095
**8**	94.68 ± 3.19	2.87	−7.0863	38.07	2.32
**9**	-	2.24	−7.0808	167	42.04
**10**	-	2.21	−6.8554	8.67	2.56
**11**	15.89 ± 0.47	2.19	−8.3304	270.1	450.64
**12**	-	1.55	−8.0801	58.29	8216.3
**13**	60.63 ± 2.62	1.53	−10.9856	62.47	506.31
**14**	-	2.03	−11.0470	19.29	150.88
**15**	-	2.49	−8.4781	122.4	7.72
**16**	-	1.86	−7.8216	538	140.39
**17**	-	1.36	−9.8182	1132	926.75
**18**	-	2.53	−9.4939	255.5	50.55
**19**	-	1.53	−11.2594	62.47	506.31
**20**	-	2.62	−9.7035	133.6	46.01

^a^ The cytotoxic effects (IC_50_, μM) of the tadalafil derivatives on normal cells using rat aortic smooth muscle cells (RASMCs).

## Data Availability

The original contributions presented in this study are included in the article/Supplementary Material. Further inquiries can be directed to the corresponding author(s).

## Supplementary Materials

The following supporting information can be downloaded at: https://www.mdpi.com/article/10.3390/molecules31203785/s1, Figure S1: ^1^H-NMR spectrum of compound **2** in DMSO-*d*6; Figure S2: ^13^C-NMR spectrum of compound **2** in DMSO-*d*6; Figure S3: LC-MS spectrum of compound **2** in methanol; Figure S4: HPLC purity of compound **2**; Figure S5: ^1^H-NMR spectrum of compound **3** in DMSO-*d*6; Figure S6: ^13^C-NMR spectrum of compound **3** in DMSO-*d*6; Figure S7: LC-MS spectrum of compound **3** in methanol; Figure S8: HPLC purity of compound **3**; Figure S9: ^1^H-NMR spectrum of compound **4** in DMSO-*d*6; Figure S10: ^13^C-NMR spectrum of compound **4** in DMSO-*d*6; Figure S11: LC-MS spectrum of compound **4** in methanol; Figure S12: HPLC purity of compound **4**; Figure S13: ^1^H-NMR spectrum of compound **5** in DMSO-*d*6; Figure S14: ^13^C-NMR spectrum of compound **5** in DMSO-*d*6; Figure S15: LC-MS spectrum of compound **5** in methanol; Figure S16: HPLC purity of compound **5**; Figure S17: ^1^H-NMR spectrum of compound **6** in DMSO-*d*6; Figure S18: ^13^C-NMR spectrum of compound **6** in DMSO-*d*6; Figure S19: LC-MS spectrum of compound **6** in methanol; Figure S20: HPLC purity of compound **6**; Figure S21: ^1^H-NMR spectrum of compound **7** in DMSO-*d*6; Figure S22: ^13^C-NMR spectrum of compound **7** in DMSO-*d*6; Figure S23: LC-MS spectrum of compound **7** in methanol; Figure S24: HPLC purity of compound **7**; Figure S25: ^1^H-NMR spectrum of compound **8** in DMSO-*d*6; Figure S26: ^13^C-NMR spectrum of compound **8** in DMSO-*d*6; Figure S27: LC-MS spectrum of compound **8** in methanol; Figure S28: HPLC purity of compound **8**; Figure S29: ^1^H-NMR spectrum of compound **9** in DMSO-*d*6; Figure S30: ^13^C-NMR spectrum of compound **9** in DMSO-*d*6; Figure S31: LC-MS spectrum of compound **9** in methanol; Figure S32: HPLC purity of compound **9**; Figure S33: ^1^H-NMR spectrum of compound **10** in DMSO-*d*6; Figure S34: ^13^C-NMR spectrum of compound **10** in DMSO-*d*6; Figure S35: LC-MS spectrum of compound **10** in methanol; Figure S36: HPLC purity of compound **10**; Figure S37: ^1^H-NMR spectrum of compound **11** in DMSO-*d*6; Figure S38: ^13^C-NMR spectrum of compound **11** in DMSO-*d*6; Figure S39: LC-MS spectrum of compound **11** in methanol; Figure S40: HPLC purity of compound **11**; Figure S41: ^1^H-NMR spectrum of compound **12** in DMSO-*d*6; Figure S42: ^13^C-NMR spectrum of compound **12** in DMSO-*d*6; Figure S43: LC-MS spectrum of compound **12** in methanol; Figure S44: HPLC purity of compound **12**; Figure S45: ^1^H-NMR spectrum of compound **13** in DMSO-*d*6; Figure S46: ^13^C-NMR spectrum of compound **13** in DMSO-*d*6; Figure S47: LC-MS spectrum of compound **13** in methanol; Figure S48: HPLC purity of compound **13**; Figure S49: ^1^H-NMR spectrum of compound **14** in DMSO-*d*6; Figure S50: ^13^C-NMR spectrum of compound **14** in DMSO-*d*6; Figure S51: LC-MS spectrum of compound **14** in methanol; Figure S52: HPLC purity of compound **14**; Figure S53: ^1^H-NMR spectrum of compound **15** in DMSO-*d*6; Figure S54: ^13^C-NMR spectrum of compound **15** in DMSO-*d*6; Figure S55: LC-MS spectrum of compound **15** in methanol; Figure S56: HPLC purity of compound **15**; Figure S57: ^1^H-NMR spectrum of compound **16** in DMSO-*d*6; Figure S58: ^13^C-NMR spectrum of compound **16** in DMSO-*d*6; Figure S59: LC-MS spectrum of compound **16** in methanol; Figure S60: HPLC purity of compound **16**; Figure S61: ^1^H-NMR spectrum of compound **17** in DMSO-*d*6; Figure S62: ^13^C-NMR spectrum of compound **17** in DMSO-*d*6; Figure S63: LC-MS spectrum of compound **17** in methanol; Figure S64: HPLC purity of compound **17**; Figure S65: ^1^H-NMR spectrum of compound **18** in DMSO-*d*6; Figure S66: ^13^C-NMR spectrum of compound **18** in DMSO-*d*6; Figure S67: LC-MS spectrum of compound **18** in methanol; Figure S68: HPLC purity of compound **18**; Figure S69: ^1^H-NMR spectrum of compound **19** in DMSO-*d*6; Figure S70: ^13^C-NMR spectrum of compound **19** in DMSO-*d*6; Figure S71: LC-MS spectrum of compound **19** in methanol; Figure S72: HPLC purity of compound **19**; Figure S73: ^1^H-NMR spectrum of compound **20** in DMSO-*d*6; Figure S74: ^13^C-NMR spectrum of compound **20** in DMSO-*d*6; Figure S75: LC-MS spectrum of compound **20** in methanol; Figure S76: HPLC purity of compound **20**.
