# Supplementary material for: Design, Synthesis, Evaluation, and Molecular Dynamics Simulation of Tadalafil Derivatives for the Treatment of Erectile Dysfunction
Source: Molecules. 2026 Oct 21;31(20):3785. doi: 10.3390/molecules31203785 (PMC13707866; doi:10.3390/molecules31203785)
Supplement: Supplementary file 1 [file molecules-31-03785-s001.zip › molecules-4575712-supplementary.pdf]

Electronic Supplemental Information

Design, Synthesis, Evaluation, and Molecular Dynamics Simulation of Tadalafil Derivatives for the Treatment of Erectile Dysfunction

Wentao Liu[1,2,†], Bin Zhang[1,2,†], Yuping Jia[1,2], Xiangyu Kong[1,2], Yuxiao Deng[1,2], Xinqiang Cui[1,2], Li Yang[1,2], Wenjie Ren[1,2], Xuan Tang[1,2], Daizhou Zhang[1,2,*], Lingwen Xu[1,2,*]

1. Institute of Chemical Drugs, Shandong Academy of Pharmaceutical Sciences, Jinan 250101, China;

2. Shandong Engineering Research Center of Pharmaceutical Green Intelligent Technology, Shandong Academy of Pharmaceutical Sciences, Jinan 250101, China.

† These authors contributed equally to this work.

* Correspondence: zhangdaizhou@sdaps.cn; lingwenxu2022@163.com

Table of contents:

**Fig. S1**. ^{1}H-NMR spectrum of compound **2** in DMSO-d_6.	5
**Fig. S2**. ^{13}C-NMR spectrum of compound **2** in DMSO-d_6.	5
**Fig. S3**. LC-MS spectrum of compound **2** in methanol.	6
**Fig. S4** HPLC purity of compound **2**.	6
**Fig. S5**. ^{1}H-NMR spectrum of compound **3** in DMSO-d_6.	7
**Fig. S6**. ^{13}C-NMR spectrum of compound **3** in DMSO-d_6.	7
**Fig. S7**. LC-MS spectrum of compound **3** in methanol.	8
**Fig. S8**. HPLC purity of compound **3**.	8
**Fig. S9**. ^{1}H-NMR spectrum of compound **4** in DMSO-d_6.	9
**Fig. S10**. ^{13}C-NMR spectrum of compound **4** in DMSO-d_6.	9

**Fig. S11**. LC-MS spectrum of compound**4** in methanol.	10
**Fig. S12** HPLC purity of compound **4**.	10
**Fig. S13**. ^{1}H-NMR spectrum of compound **5** in DMSO-d_6.	11
**Fig. S14**. ^{13}C-NMR spectrum of compound **5** in DMSO-d_6.	11
**Fig. S15**. LC-MS spectrum of compound **5** in methanol.	12
**Fig. S16**. HPLC purity of compound **5**.	12
**Fig. S17**. ^{1}H-NMR spectrum of compound **6** in DMSO-d_6.	13
**Fig. S18**. ^{13}C-NMR spectrum of compound **6** in DMSO-d_6.	13
**Fig. S19**. LC-MS spectrum of compound **6** in methanol.	14
**Fig. S20**. HPLC purity of compound **6**.	14
**Fig. S21**. ^{1}H-NMR spectrum of compound **7** in DMSO-d_6.	15
**Fig. S22**. ^{13}C-NMR spectrum of compound **7** in DMSO-d_6.	15
**Fig. S23**. LC-MS spectrum of compound **7** in methanol.	16
**Fig. S24**. HPLC purity of compound **7**.	16
**Fig. S25**. ^{1}H-NMR spectrum of compound **8** in DMSO-d_6.	17
**Fig. S26**. ^{13}C-NMR spectrum of compound **8** in DMSO-d_6.	17
**Fig. S27**. LC-MS spectrum of compound **8** in methanol.	18
**Fig. S28**. HPLC purity of compound **8**.	18
**Fig. S29**. ^{1}H-NMR spectrum of compound **9** in DMSO-d_6.	19
**Fig. S30**. ^{13}C-NMR spectrum of compound **9** in DMSO-d_6.	19
**Fig. S31**. LC-MS spectrum of compound **9** in methanol.	20
**Fig. S32**. HPLC purity of compound **9**.	20
**Fig. S33**. ^{1}H-NMR spectrum of compound **10** in DMSO-d_6.	21
**Fig. S34**. ^{13}C-NMR spectrum of compound **10** in DMSO-d_6.	21
**Fig. S35**. LC-MS spectrum of compound **10** in methanol.	22
**Fig. S36**. HPLC purity of compound **10**.	22
**Fig. S37**. ^{1}H-NMR spectrum of compound **11** in DMSO-d_6.	23
**Fig. S38**. ^{13}C-NMR spectrum of compound **11** in DMSO-d_6.	23
**Fig. S39**. LC-MS spectrum of compound **11** in methanol.	24

**Fig. S40** HPLC purity of compound **11**.	24
**Fig. S41**. ^{1}H-NMR spectrum of compound **12** in DMSO-d_6.	25
**Fig. S42**. ^{13}C-NMR spectrum of compound **12** in DMSO-d_6.	25
**Fig. S43**. LC-MS spectrum of compound **12** in methanol.	26
**Fig. S44.** HPLC purity of compound **12**.	26
**Fig. S45**. ^{1}H-NMR spectrum of compound **13** in DMSO-d_6.	27
**Fig. S46**. ^{13}C-NMR spectrum of compound **13** in DMSO-d_6.	27
**Fig. S47**. LC-MS spectrum of compound **13** in methanol.	28
**Fig. S48.** HPLC purity of compound **13**.	28
**Fig. S49**. ^{1}H-NMR spectrum of compound **14** in DMSO-d_6.	29
**Fig. S50**. ^{13}C-NMR spectrum of compound **14** in DMSO-d_6.	29
**Fig. S51**. LC-MS spectrum of compound **14** in methanol.	30
**Fig. S52.** HPLC purity of compound **14**.	30
**Fig. S53**. ^{1}H-NMR spectrum of compound **15** in DMSO-d_6.	31
**Fig. S54**. ^{13}C-NMR spectrum of compound **15** in DMSO-d_6.	31
**Fig. S55**. LC-MS spectrum of compound **15** in methanol.	32
**Fig. S56.** HPLC purity of compound **15**.	32
**Fig. S57**. ^{1}H-NMR spectrum of compound **16** in DMSO-d_6.	33
**Fig. S58**. ^{13}C-NMR spectrum of compound **16** in DMSO-d_6.	33
**Fig. S59**. LC-MS spectrum of compound **16** in methanol.	34
**Fig. S60.** HPLC purity of compound **16**.	34
**Fig. S61**. ^{1}H-NMR spectrum of compound **17** in DMSO-d_6.	35
**Fig. S62**. ^{13}C-NMR spectrum of compound **17** in DMSO-d_6.	35
**Fig. S63**. LC-MS spectrum of compound **17** in methanol.	36
**Fig. S64.** HPLC purity of compound **17**.	36
**Fig. S65**. ^{1}H-NMR spectrum of compound **18** in DMSO-d_6.	37
**Fig. S66**. ^{13}C-NMR spectrum of compound **18** in DMSO-d_6.	37
**Fig. S67**. LC-MS spectrum of compound **18** in methanol.	38
**Fig. S68.** HPLC purity of compound **18**.	38

**Fig. S69**. ^{1}H-NMR spectrum of compound **19** in DMSO-d_6.	39
**Fig. S70**. ^{13}C-NMR spectrum of compound **19** in DMSO-d_6.	39
**Fig. S71**. LC-MS spectrum of compound **19** in methanol.	40
**Fig. S72.** HPLC purity of compound **19**.	40
**Fig. S73**. ^{1}H-NMR spectrum of compound **20** in DMSO-d_6.	41
**Fig. S74**. ^{13}C-NMR spectrum of compound **20** in DMSO-d_6.	41
**Fig. S75**. LC-MS spectrum of compound **20** in methanol.	42
**Fig. S76.** HPLC purity of compound **20**.	42

Supplementary Figures

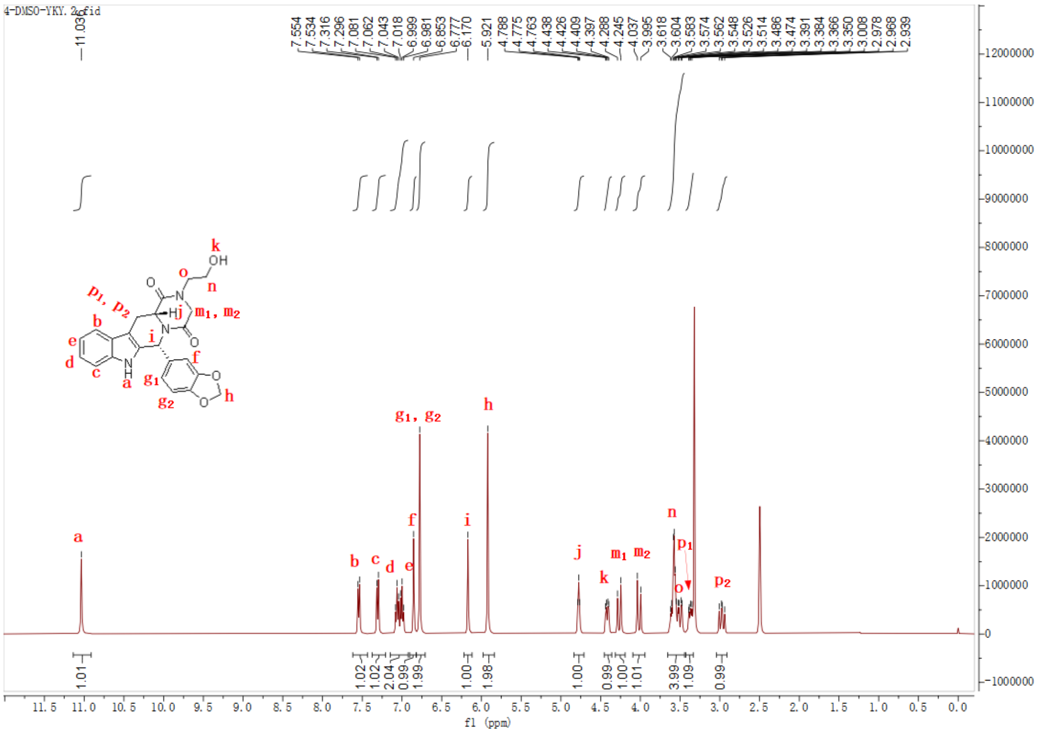

Fig. S1. ^{1}H-NMR spectrum of compound **2**in DMSO-*d*6.

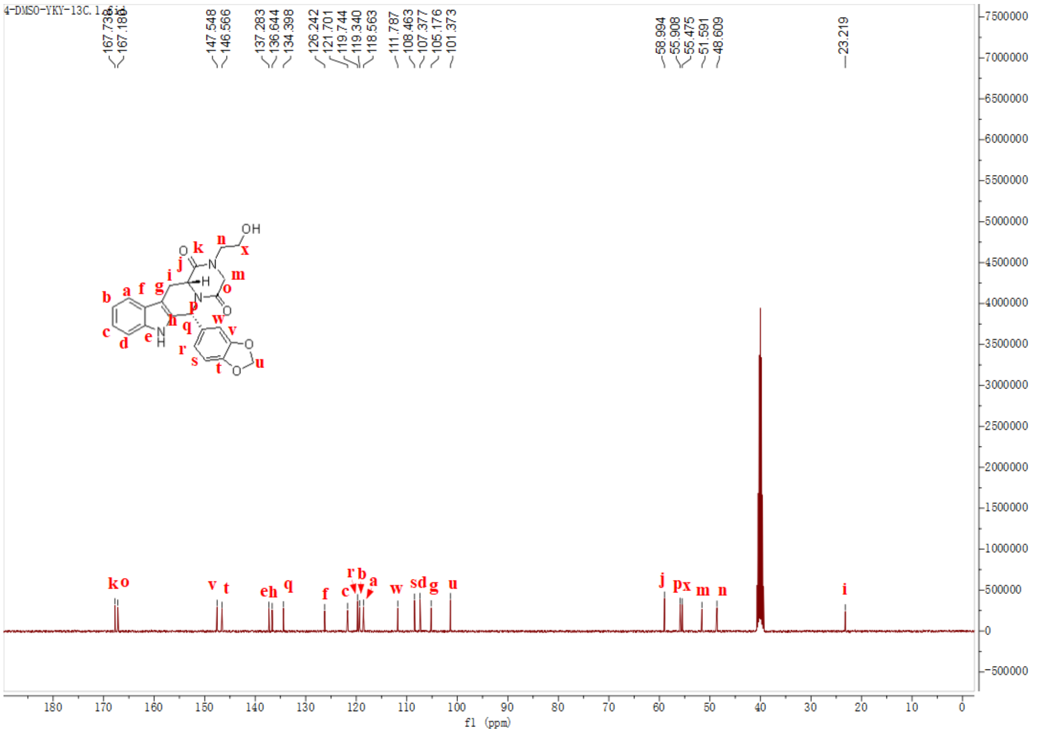

Fig. S2. ^{13}C-NMR spectrum of compound **2** in DMSO-*d*6.

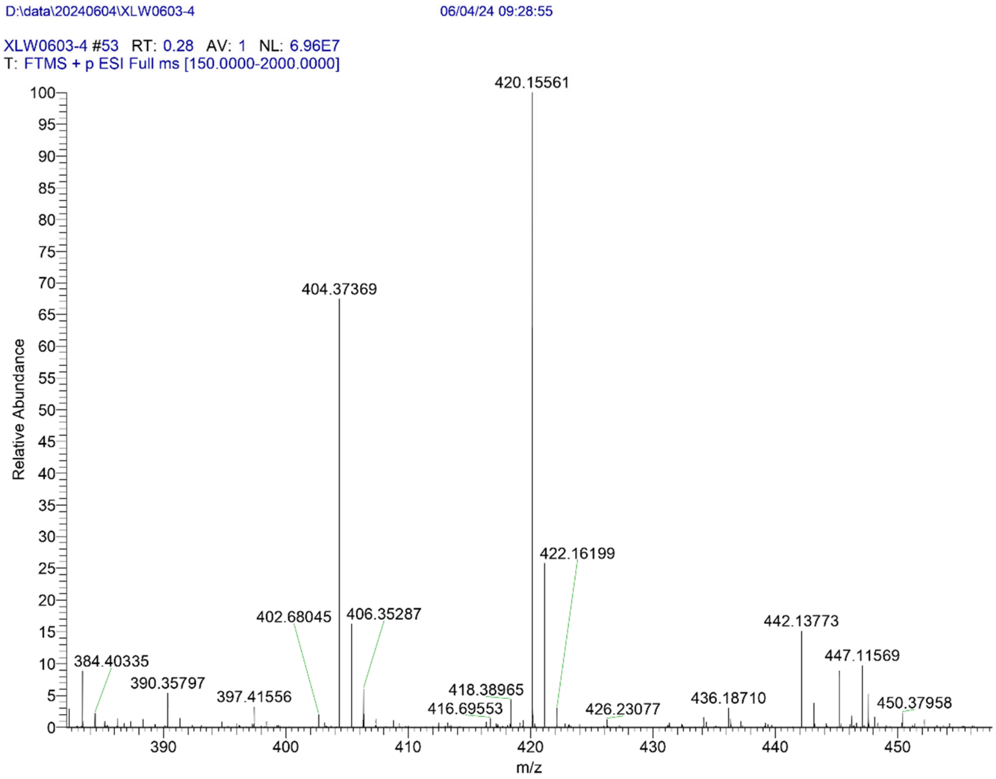

Fig. S3. LC-MS spectrum of compound **2** in methanol.

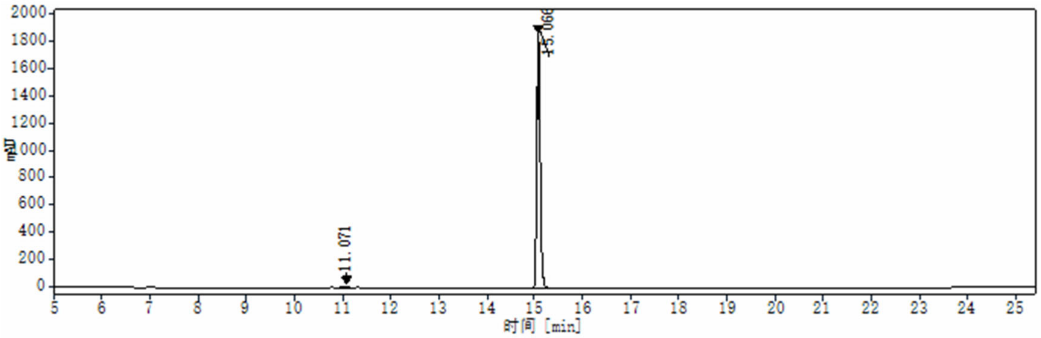

No.	Retention time	Peak area	Concentration (%)
1	11.071	105.65	1.15
2	15.066	9070.55	98.85

Fig. S4 HPLC purity of compound **2**.

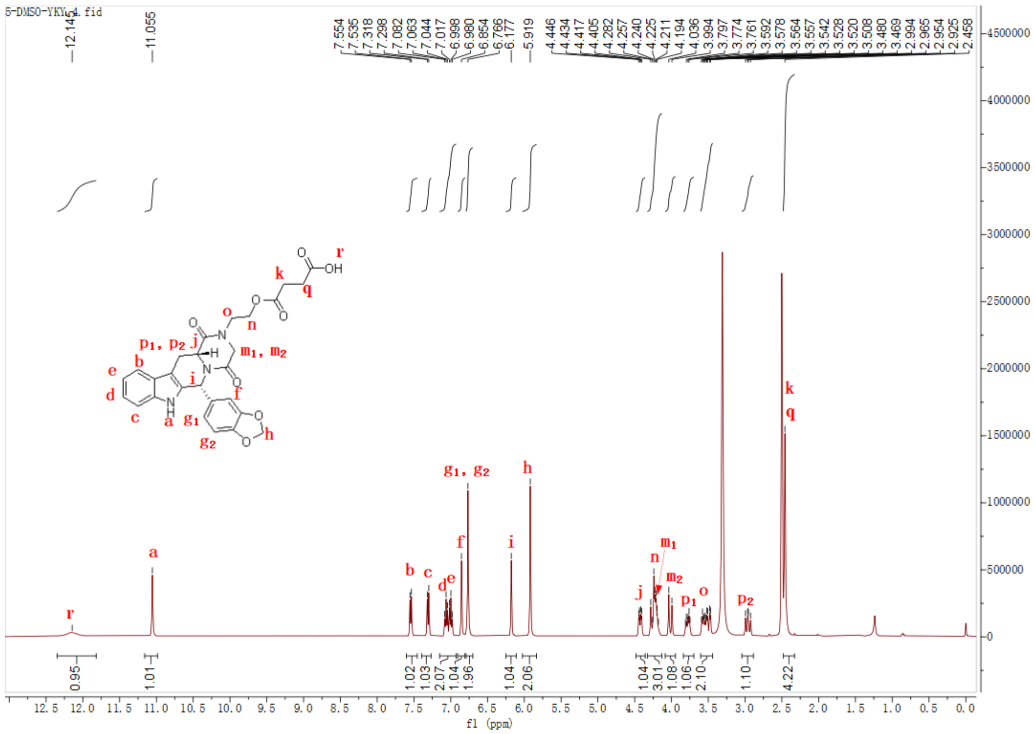

Fig. S5. ^{1}H-NMR spectrum of compound **3** in DMSO-d_6.

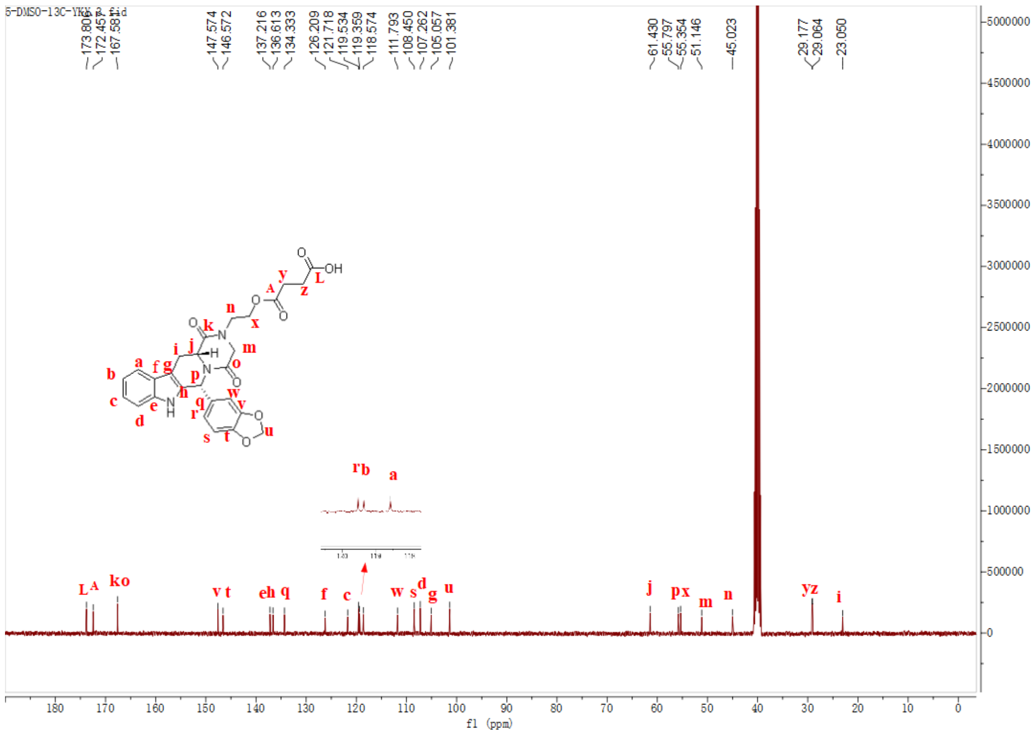

Fig. S6. ^{13}C-NMR spectrum of compound **3** in DMSO-d_6

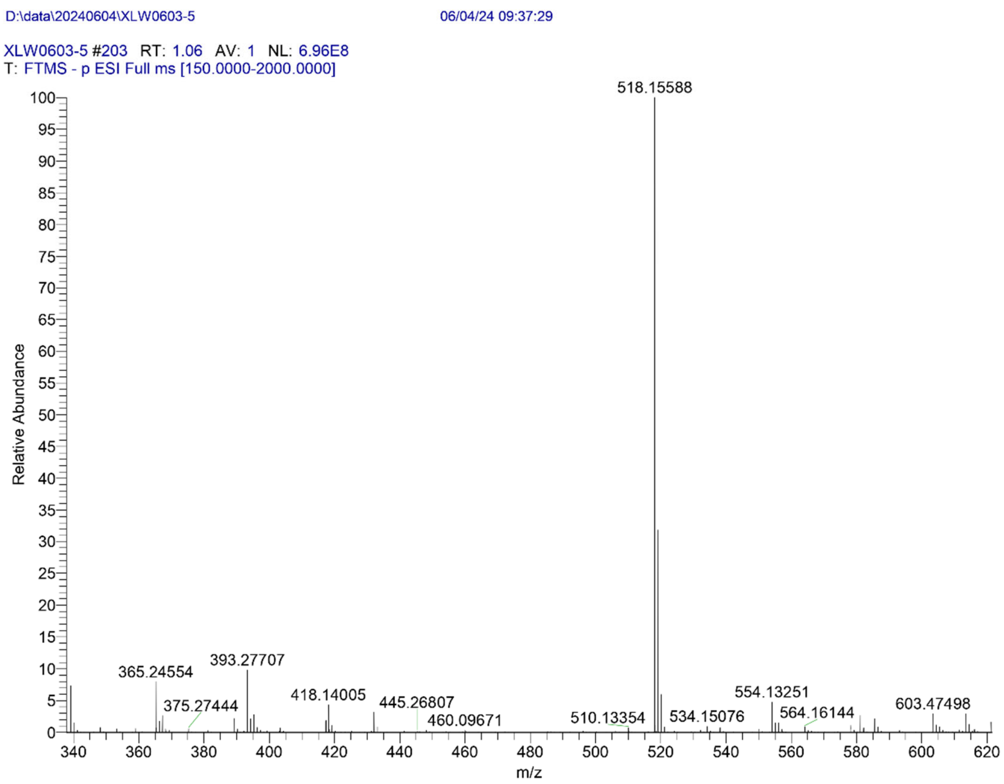

Fig. S7. LC-MS spectrum of compound **3** in methanol.

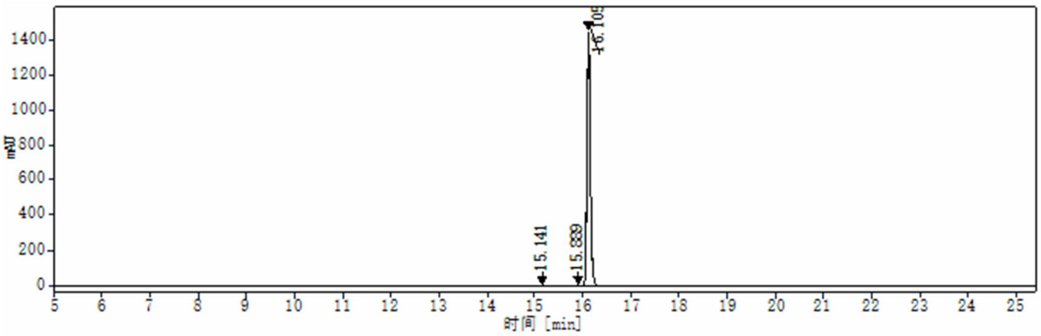

No.	Retention time	Peak area	Concentration (%)
1	15.141	4.99	0.07
2	15.889	8.72	0.12
3	16.105	7047.13	99.81

Fig. S8 HPLC purity of compound **3**.

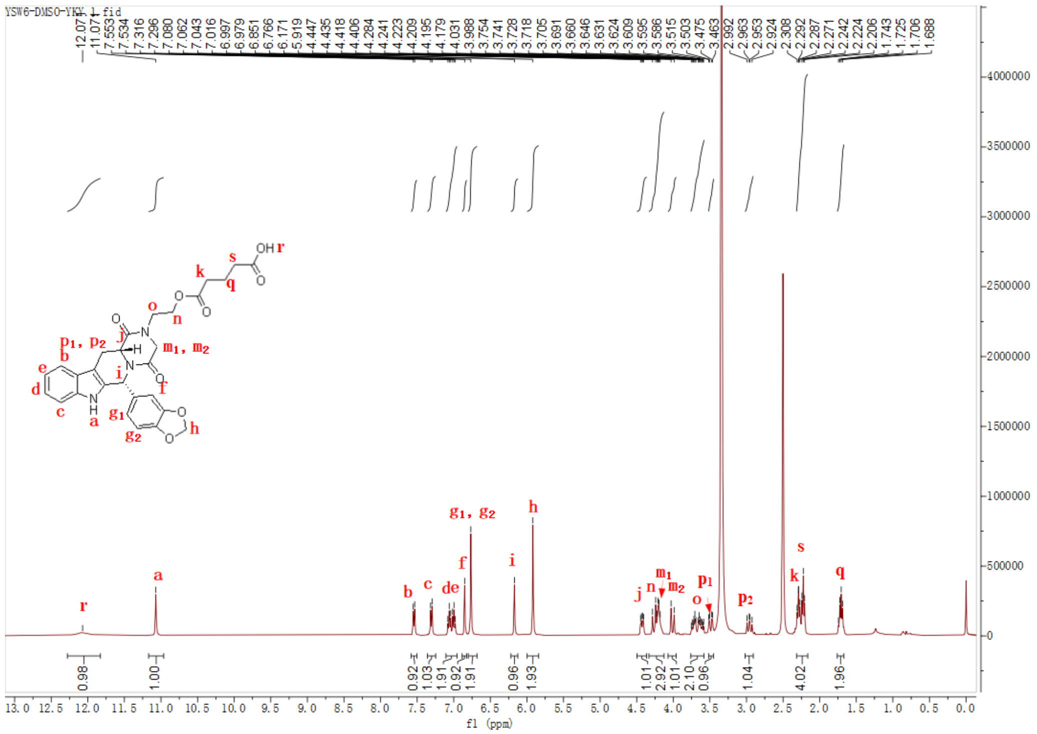

Fig. S9. ^{1}H-NMR spectrum of compound **4** in DMSO-*d*6.

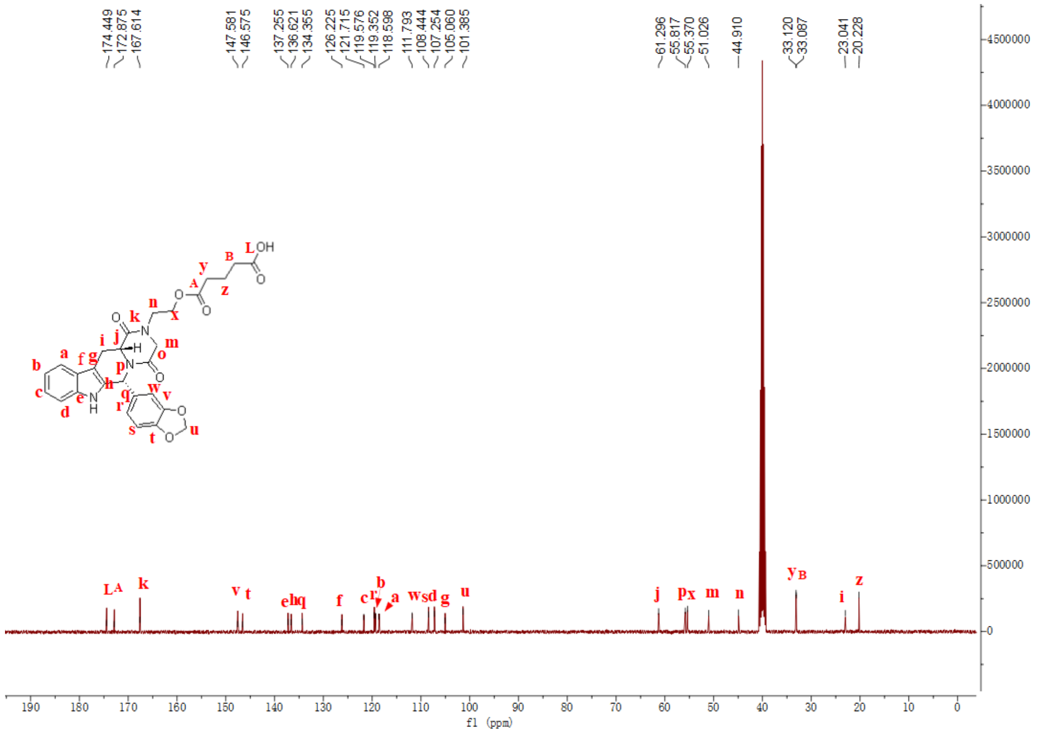

Fig. S10. ^{13}C-NMR spectrum of compound **4** in DMSO-*d*6

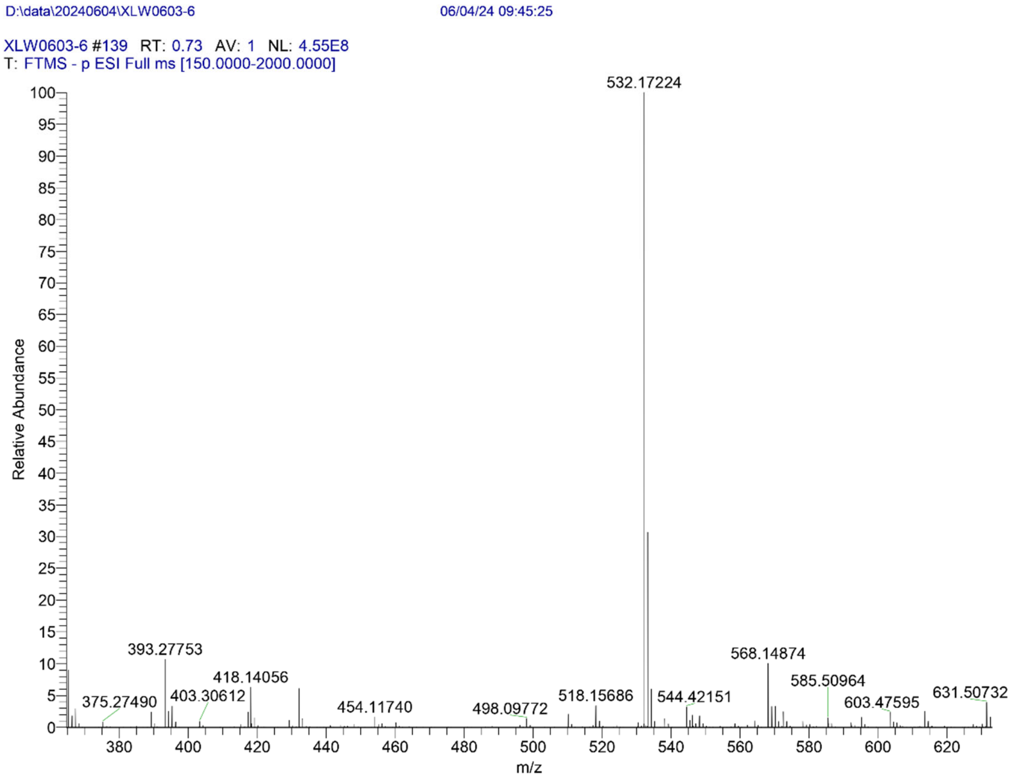

Fig. S11. LC-MS spectrum of compound **4** in methanol.

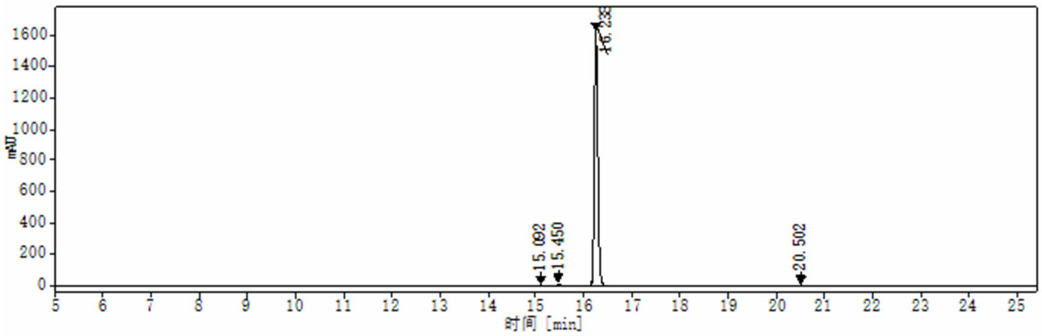

No.	Retention time	Peak area	Concentration (%)
1	15.092	19.41	0.24
2	15.450	82.23	1.02
3	16.238	7962.77	98.47
4	20.502	22.11	0.27

Fig. S12 HPLC purity of compound **4**.

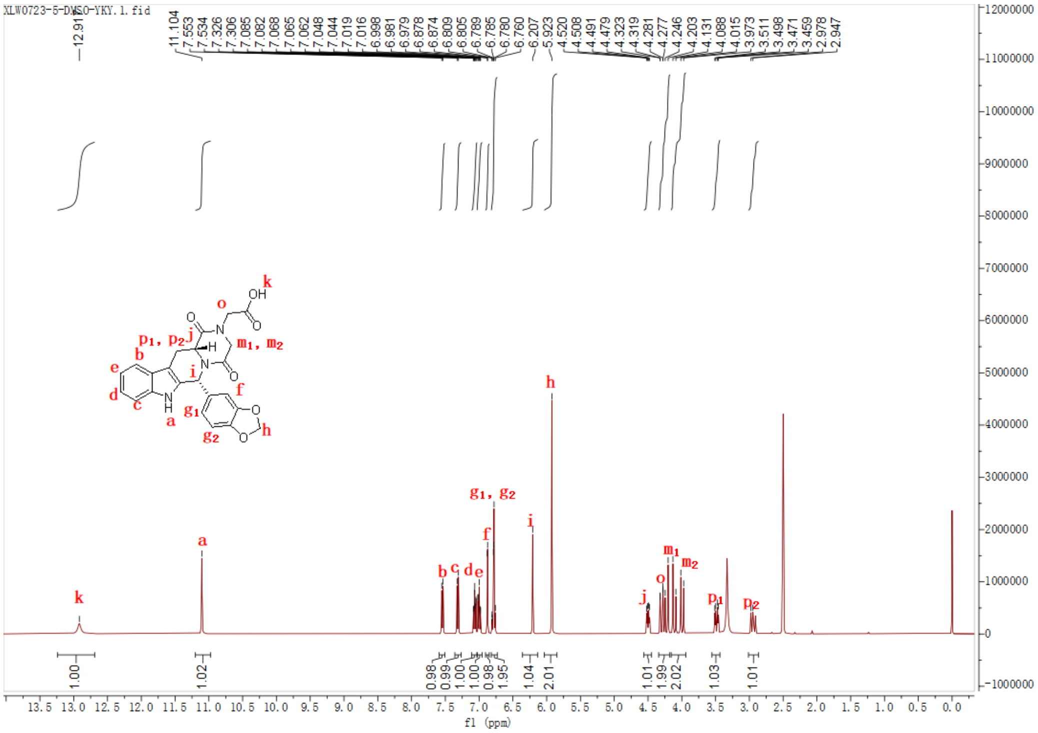

Fig. S13. ^{1}H-NMR spectrum of compound **5** in DMSO-d_6.

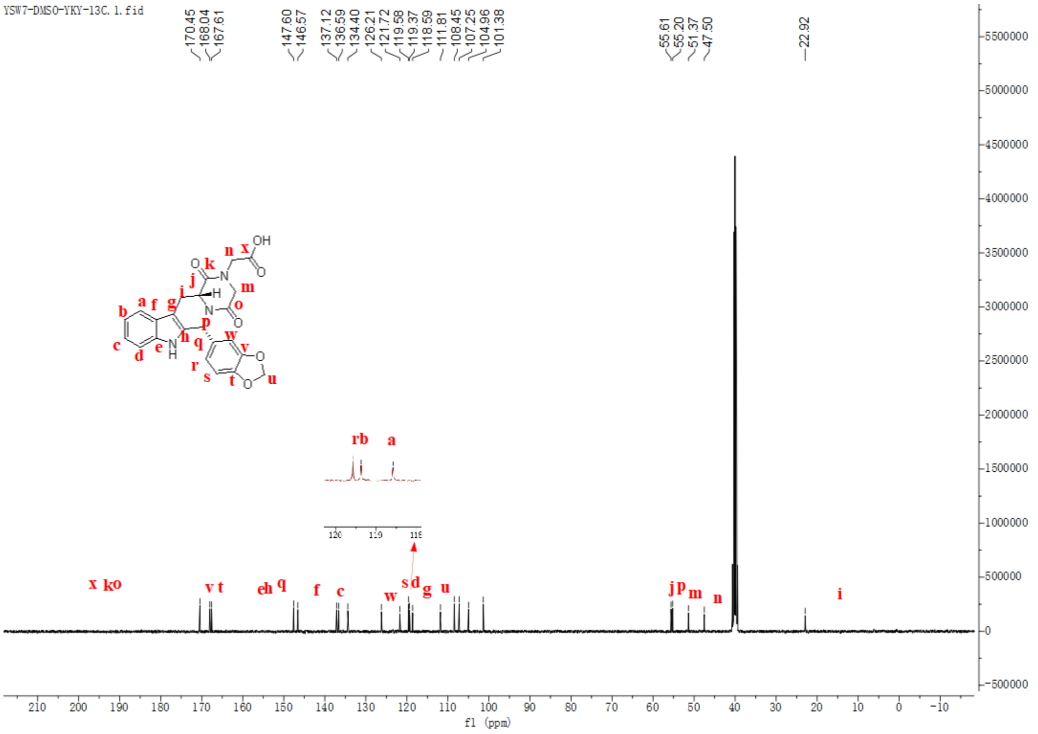

Fig. S14. ^{13}C-NMR spectrum of compound **5** in DMSO-d_6

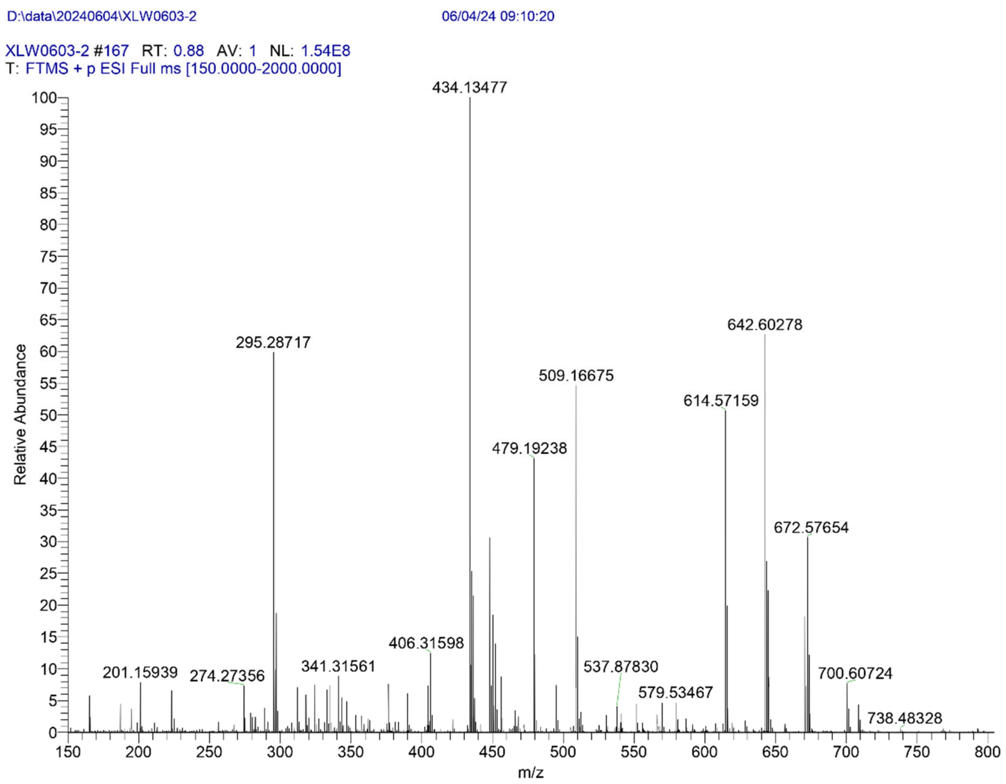

Fig. S15. LC-MS spectrum of compound **5** in methanol.

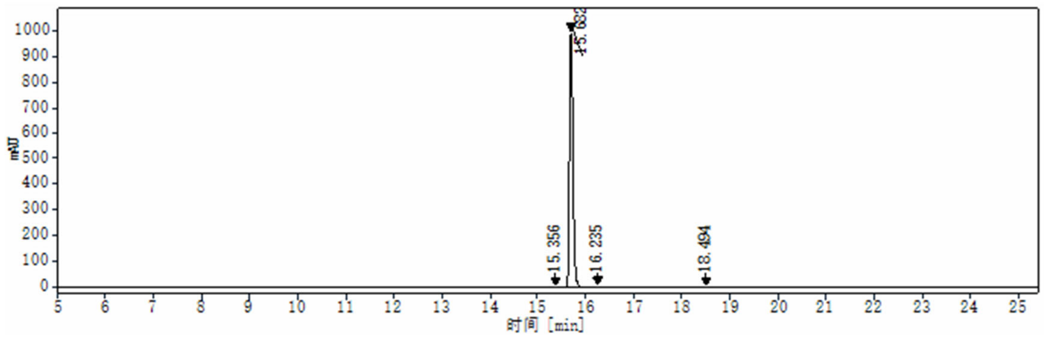

No.	Retention time	Peak area	Concentration (%)
1	15.356	2.05	0.04
2	15.682	4864.81	99.67
3	16.235	11.94	0.24
4	18.494	2.64	0.05

Fig. S16 HPLC purity of compound **5**.

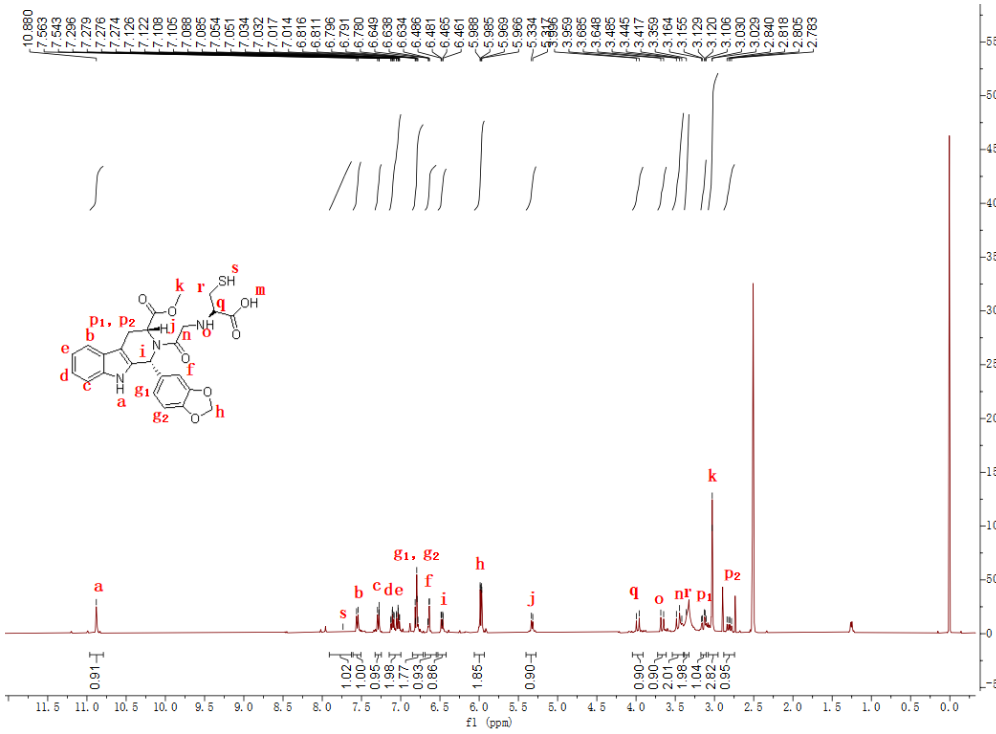

Fig. S17. ^{1}H-NMR spectrum of compound **6** in DMSO-d_6.

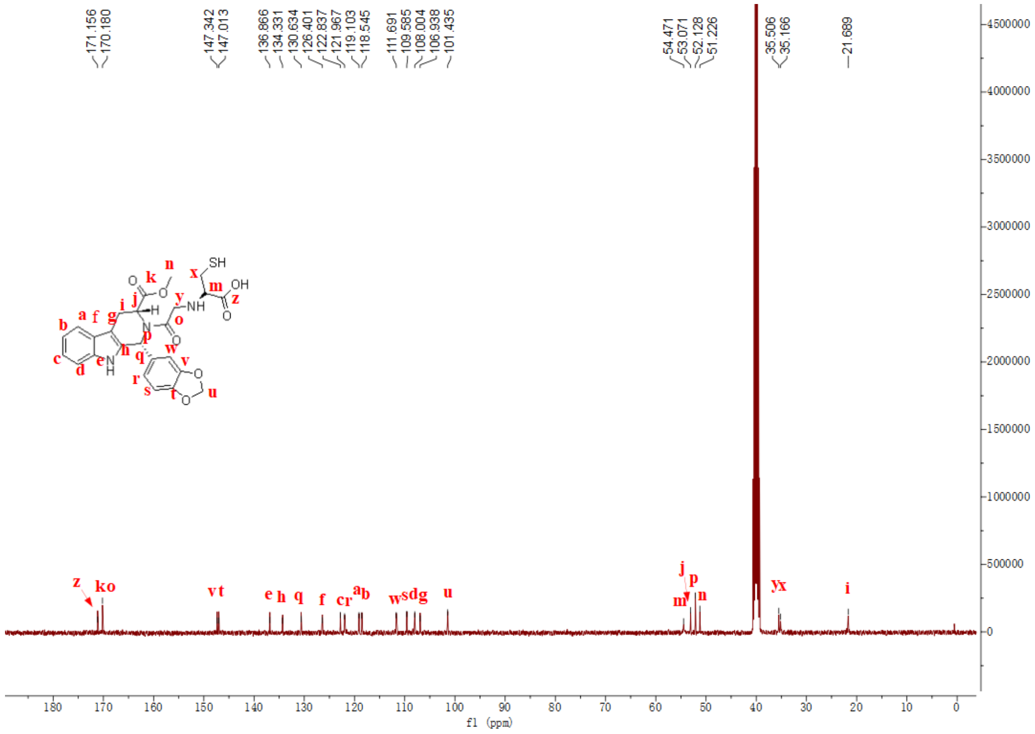

Fig. S18. ^{13}C-NMR spectrum of compound **6** in DMSO-d_6

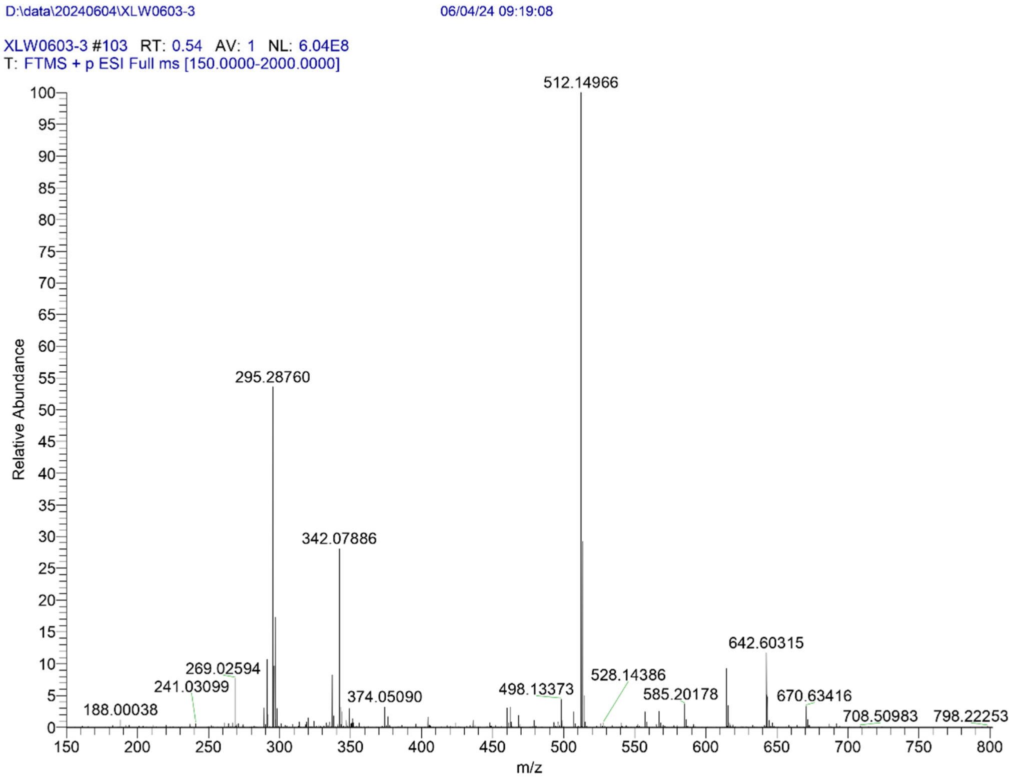

Fig. S19. LC-MS spectrum of compound **6** in methanol.

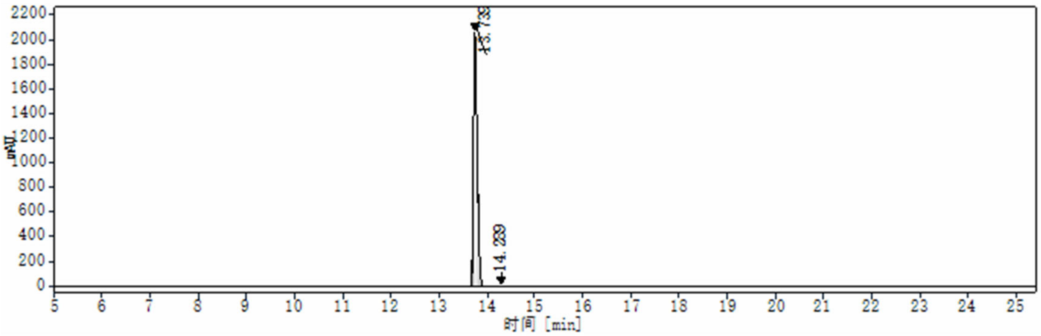

No.	Retention time	Peak area	Concentration (%)
1	13.739	11453.58	99.75
2	14.289	28.77	0.25

Fig. S20 HPLC purity of compound **6**

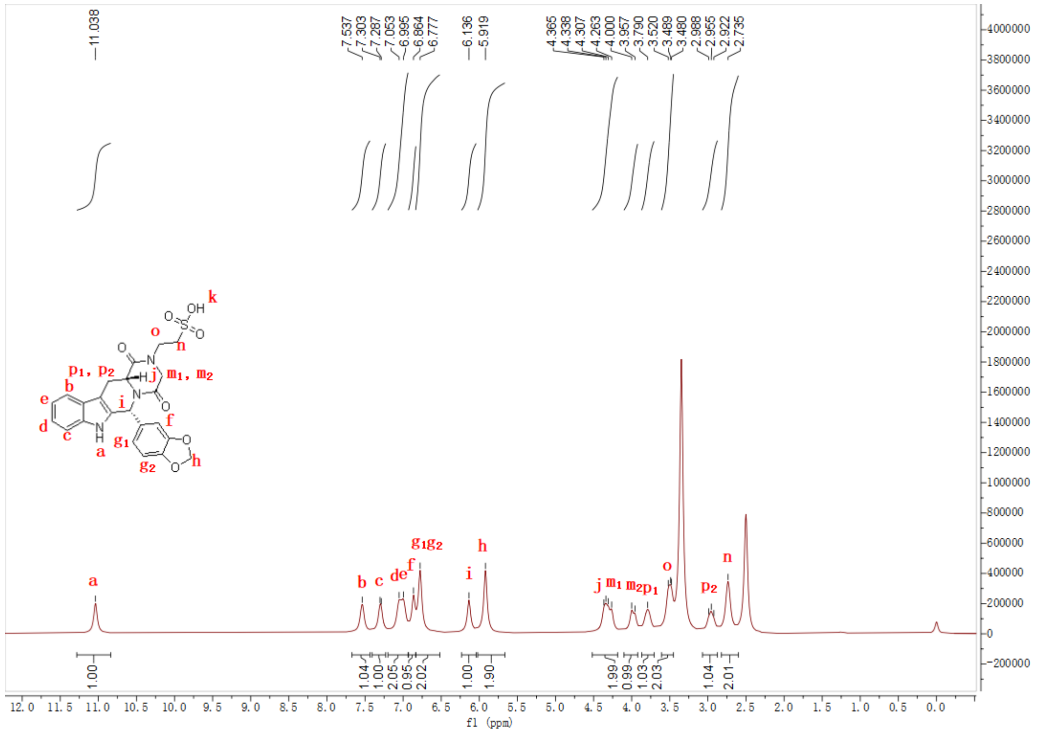

Fig. S21. ^{1}H-NMR spectrum of compound **7** in DMSO-d_6.

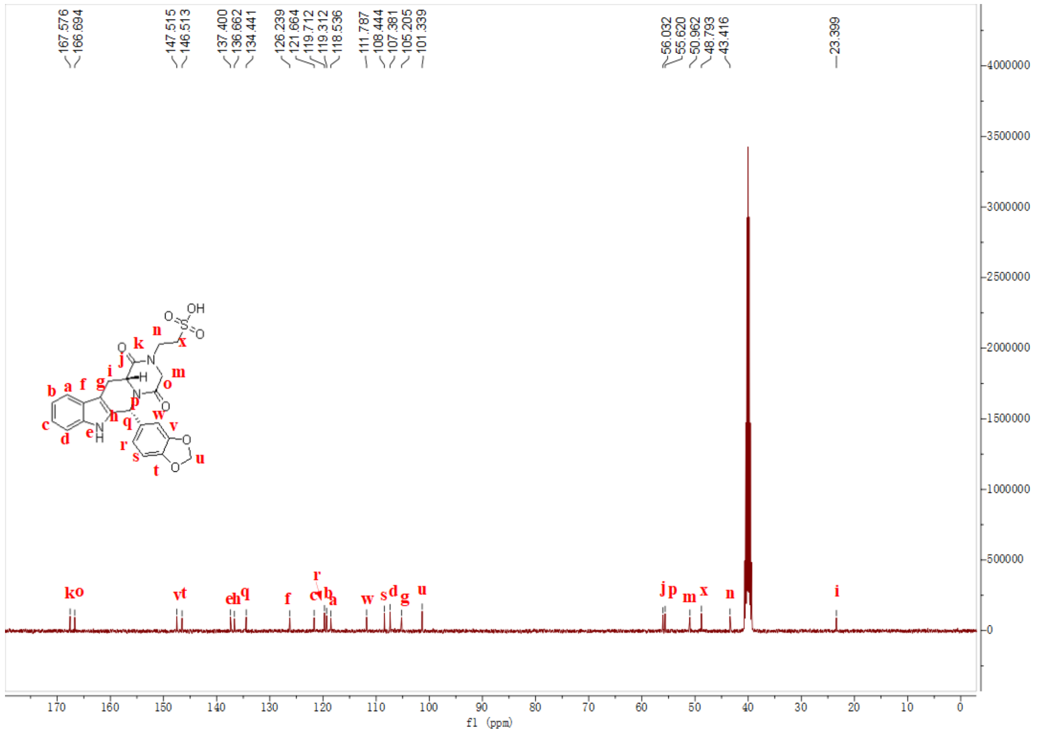

Fig. S22. ^{13}C-NMR spectrum of compound **7** in DMSO-d_6

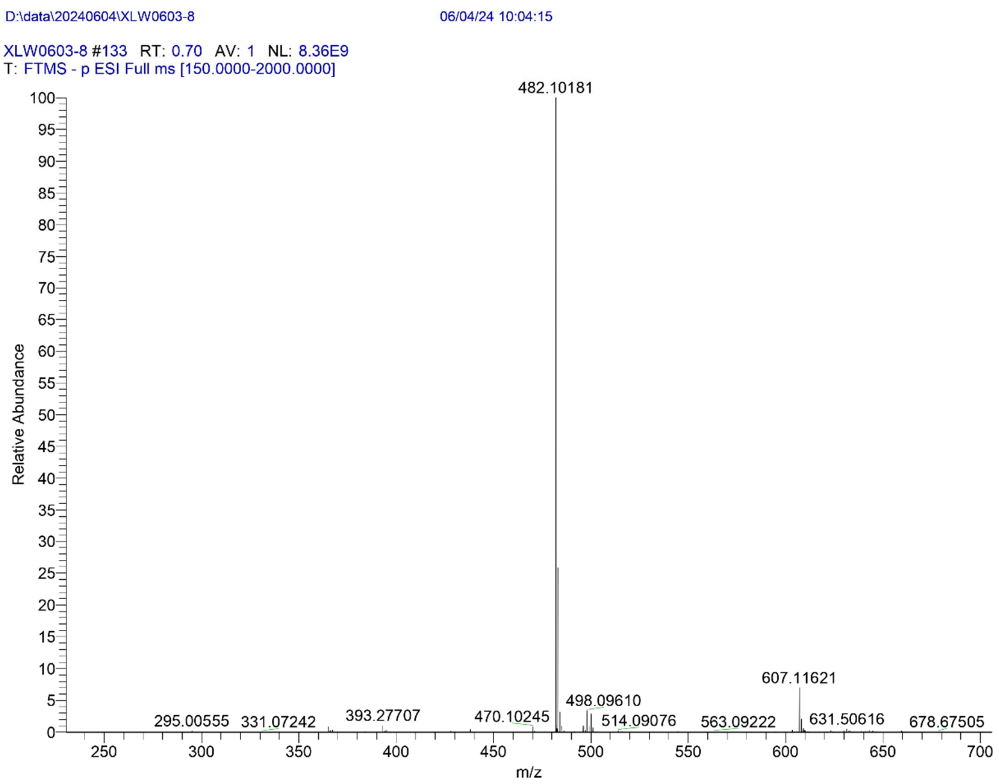

Fig. S23. LC-MS spectrum of compound **7** in methanol.

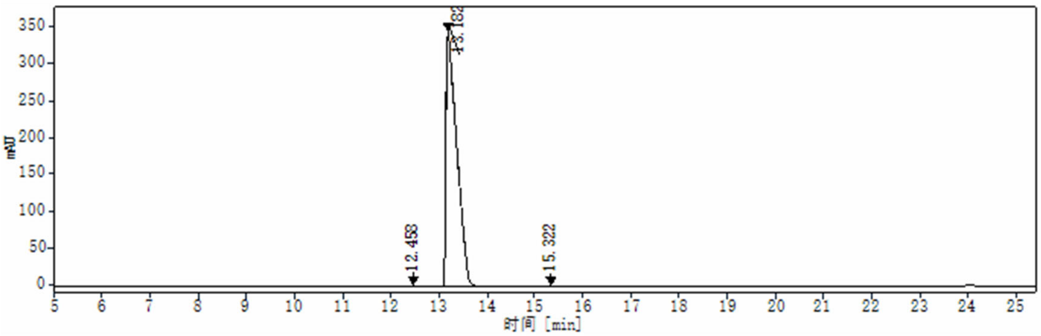

No.	Retention time	Peak area	Concentration (%)
1	12.458	4.26	0.08
2	13.182	5380.31	99.87
3	15.322	2.70	0.05

Fig. S24 HPLC purity of compound **7**

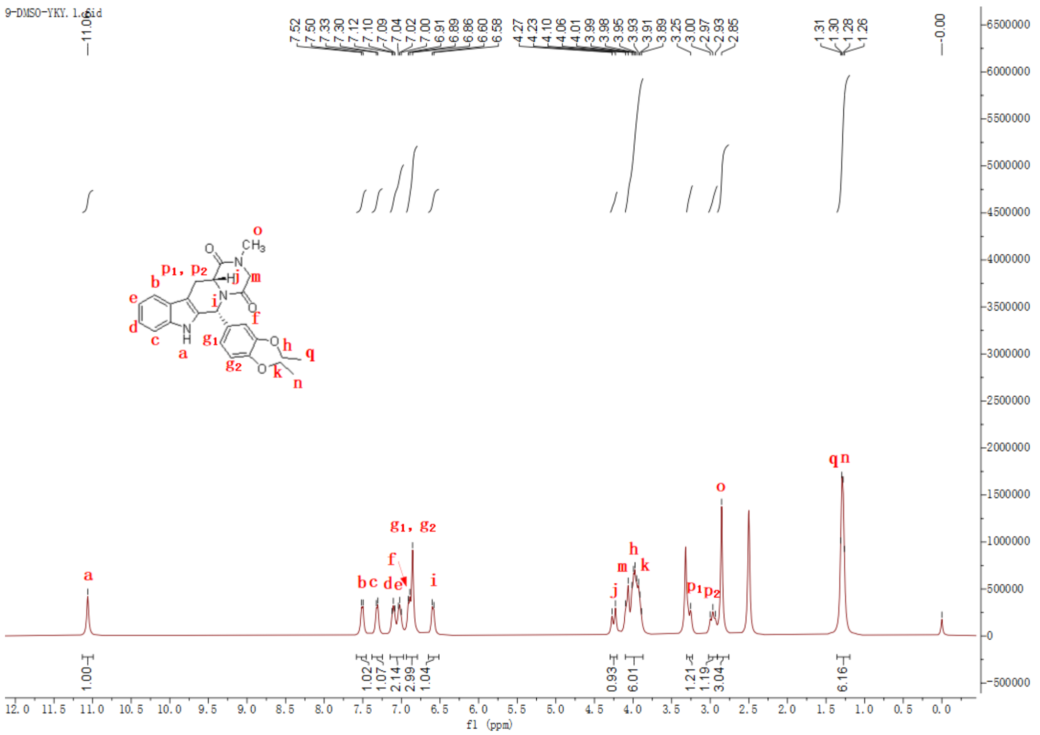

Fig. S25. ^{1}H-NMR spectrum of compound **8** in DMSO-d_6.

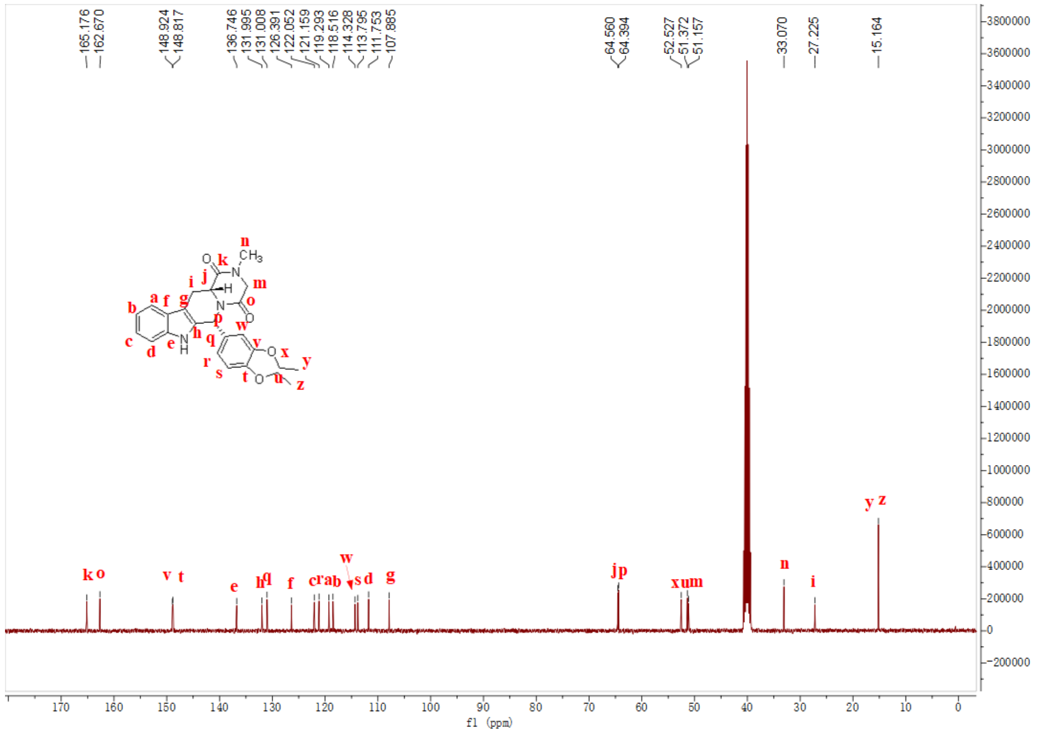

Fig. S26. ^{13}C-NMR spectrum of compound **8** in DMSO-d_6

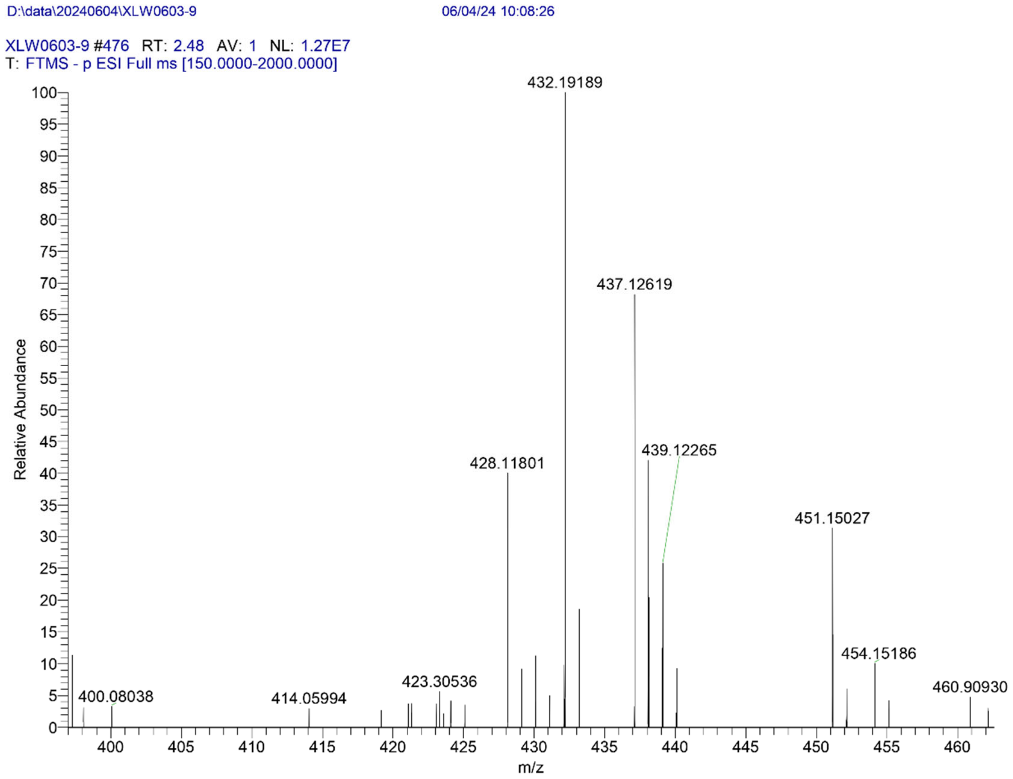

Fig. S27. LC-MS spectrum of compound **8** in methanol.

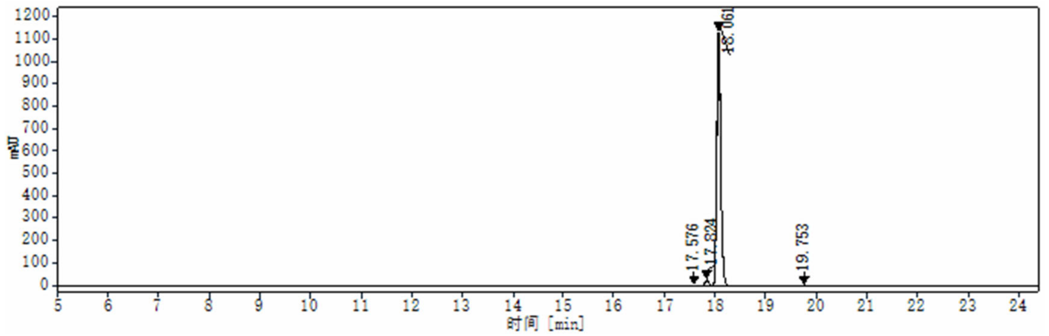

No.	Retention time	Peak area	Concentration (%)
1	17.576	16.25	0.26
2	17.824	135.26	2.17
3	18.061	6062.50	97.41
4	19.753	9.92	0.16

Fig. S28 HPLC purity of compound **8**

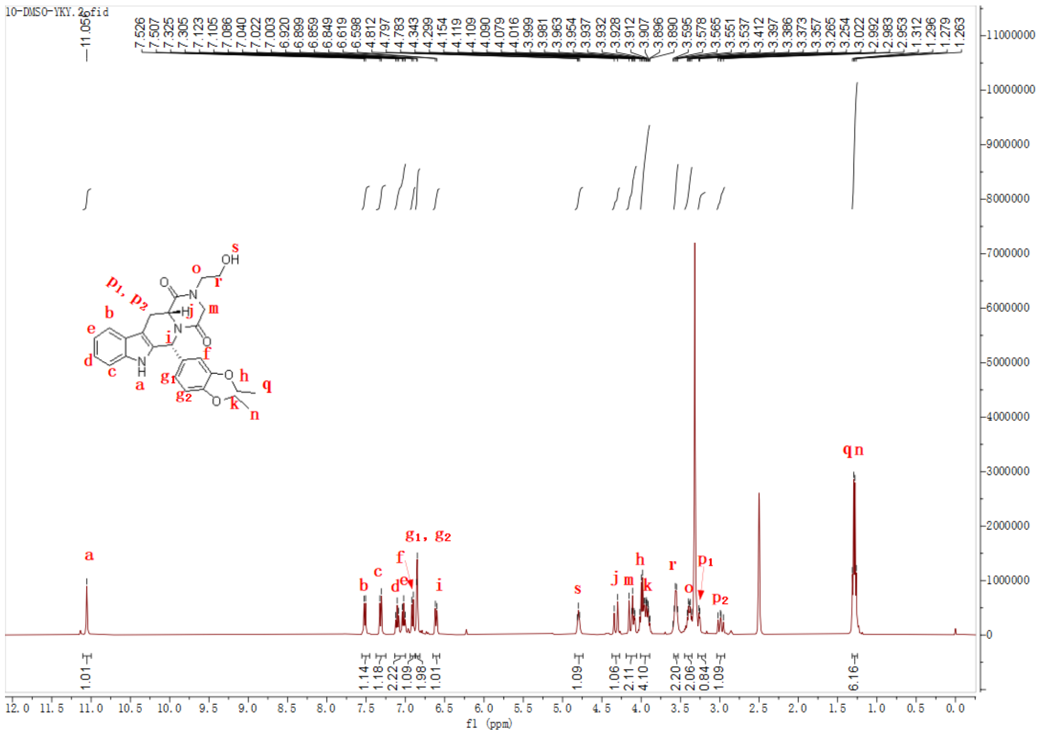

Fig. S29. ^{1}H-NMR spectrum of compound **9** in DMSO-d_6.

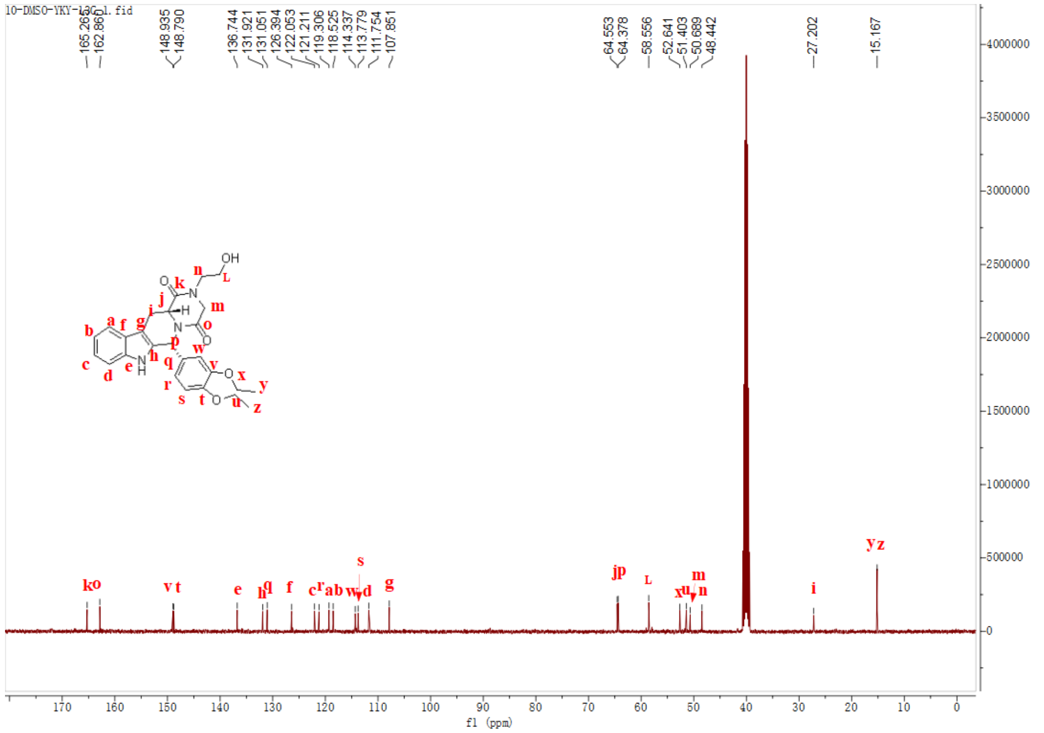

Fig. S30. ^{13}C-NMR spectrum of compound **9** in DMSO-d_6

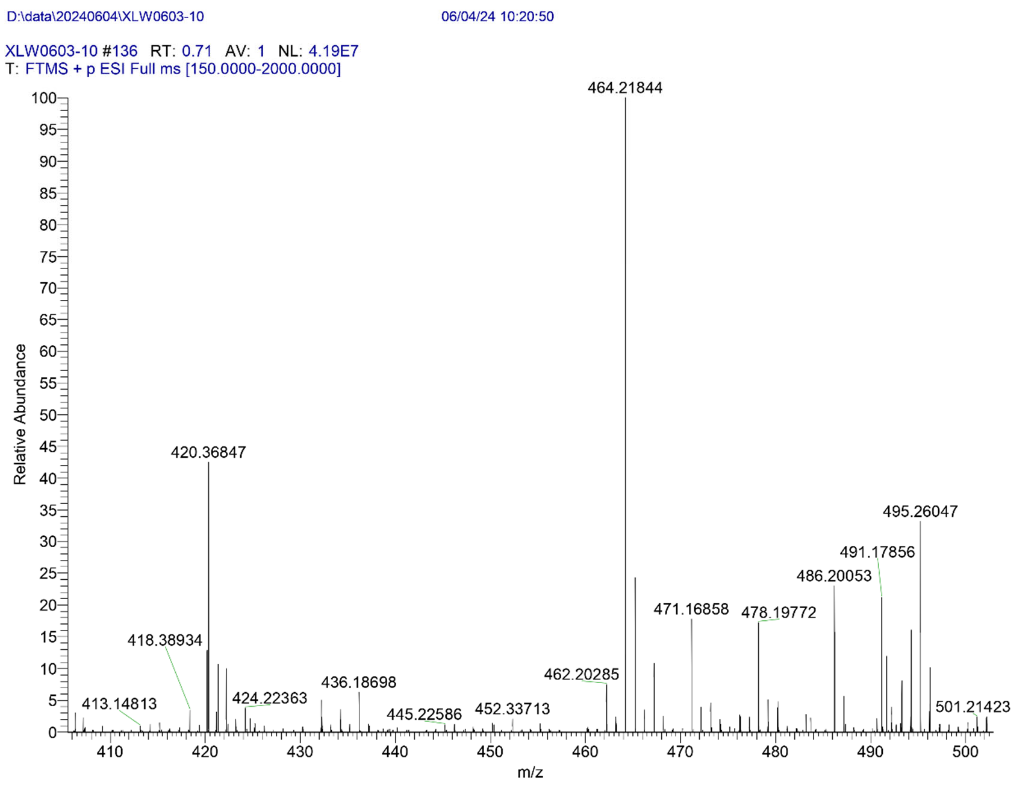

Fig. S31. LC-MS spectrum of compound **9** in methanol.

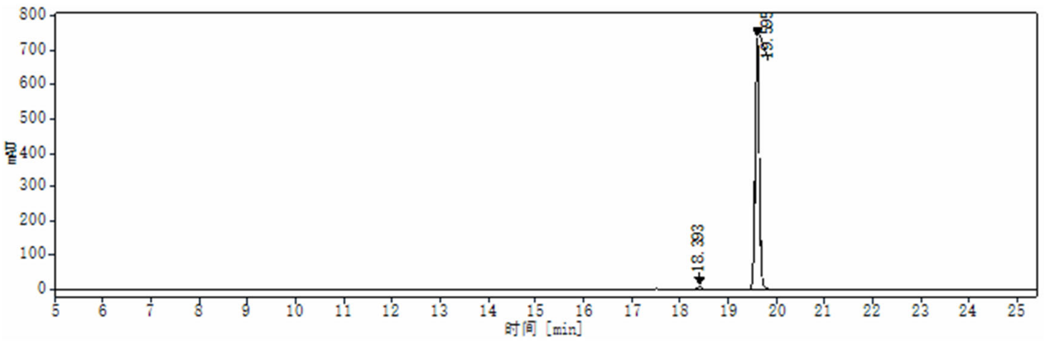

No.	Retention time	Peak area	Concentration (%)
1	18.393	53.45	1.20
2	19.595	4393.60	98.80

Fig. S32. HPLC purity of compound **9**

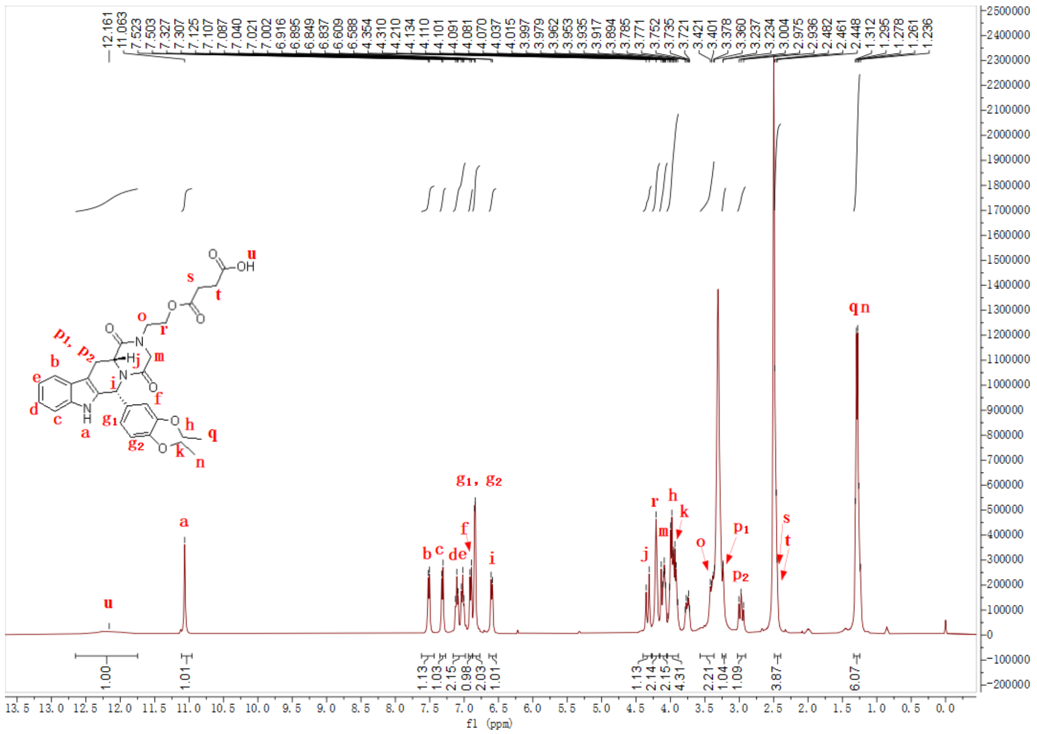

Fig. S33. ^{1}H-NMR spectrum of compound **10** in DMSO-*d*6.

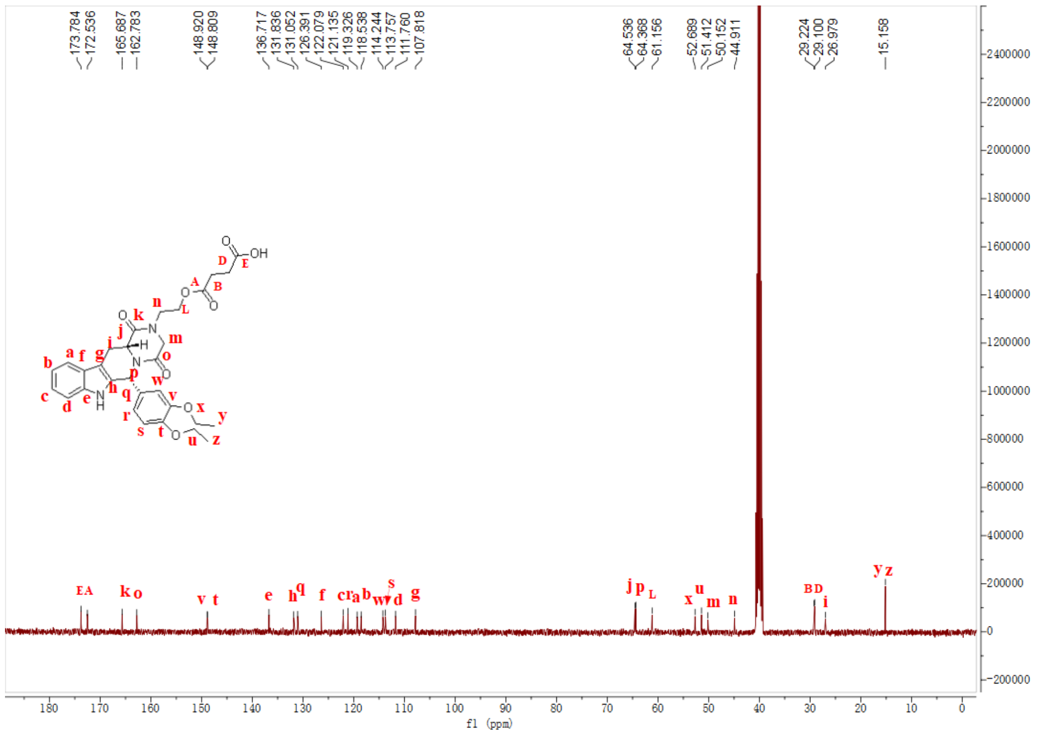

Fig. S34. ^{13}C-NMR spectrum of compound **10** in DMSO-*d*6

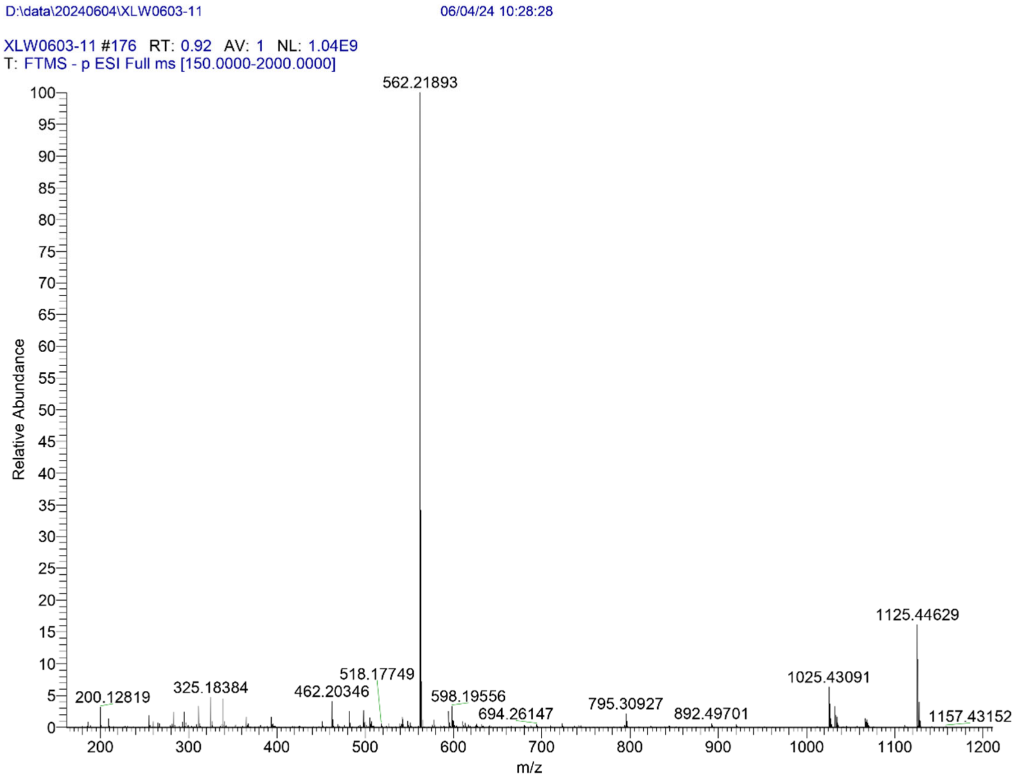

Fig. S35. LC-MS spectrum of compound **10** in methanol.

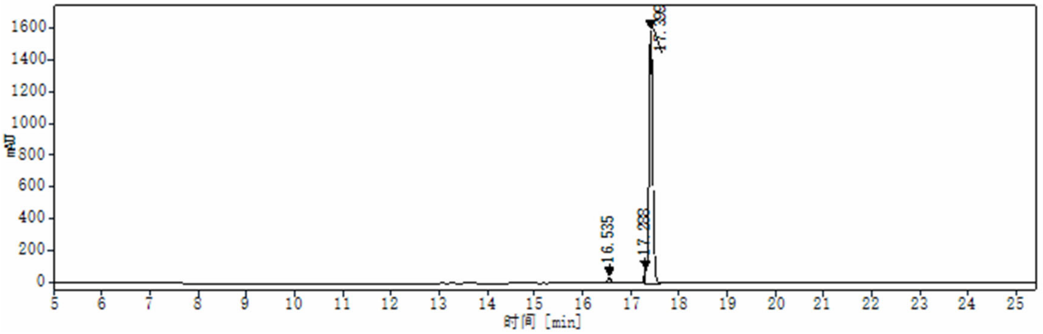

No.	Retention time	Peak area	Concentration (%)
1	16.535	156.73	1.80
2	17.288	171.70	1.97
3	17.399	8389.82	96.23

Fig. S36. HPLC purity of compound **10**

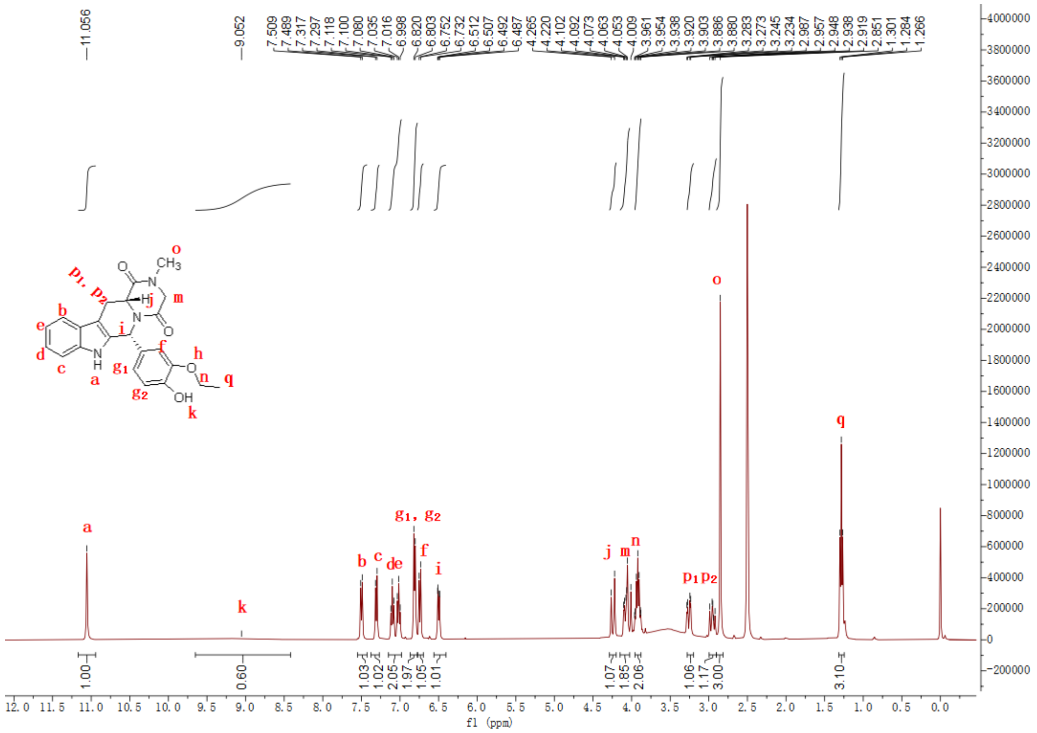

Fig. S37. ^{1}H-NMR spectrum of compound **11** in DMSO-*d*6.

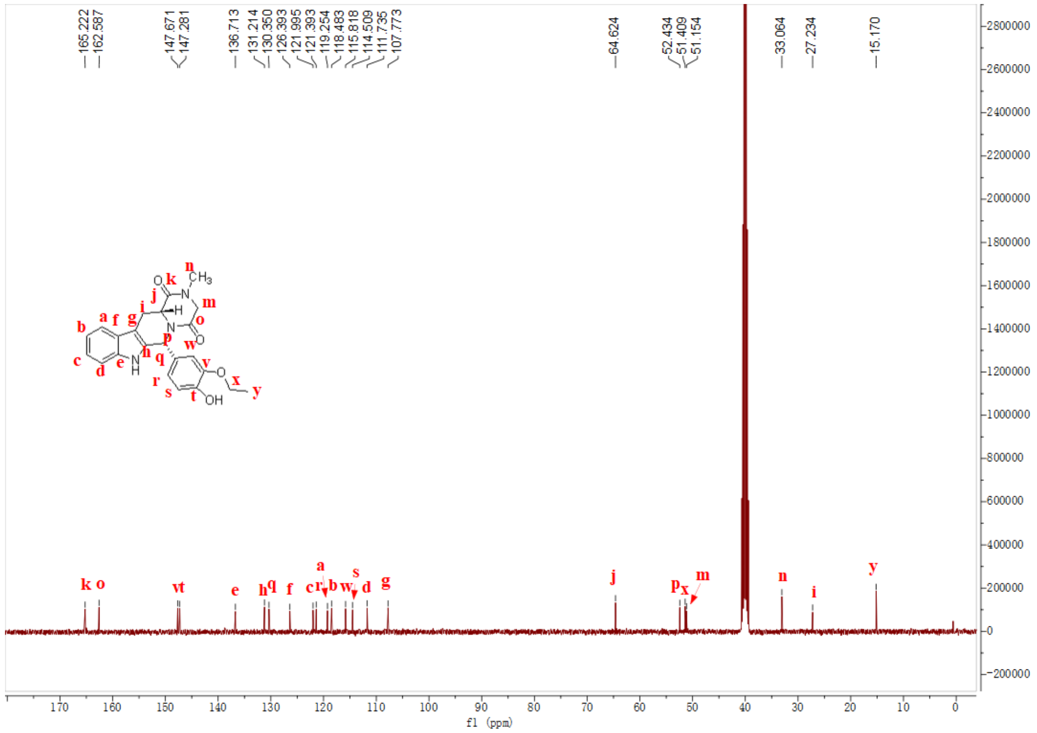

Fig. S38. ^{13}C-NMR spectrum of compound **11** in DMSO-*d*6

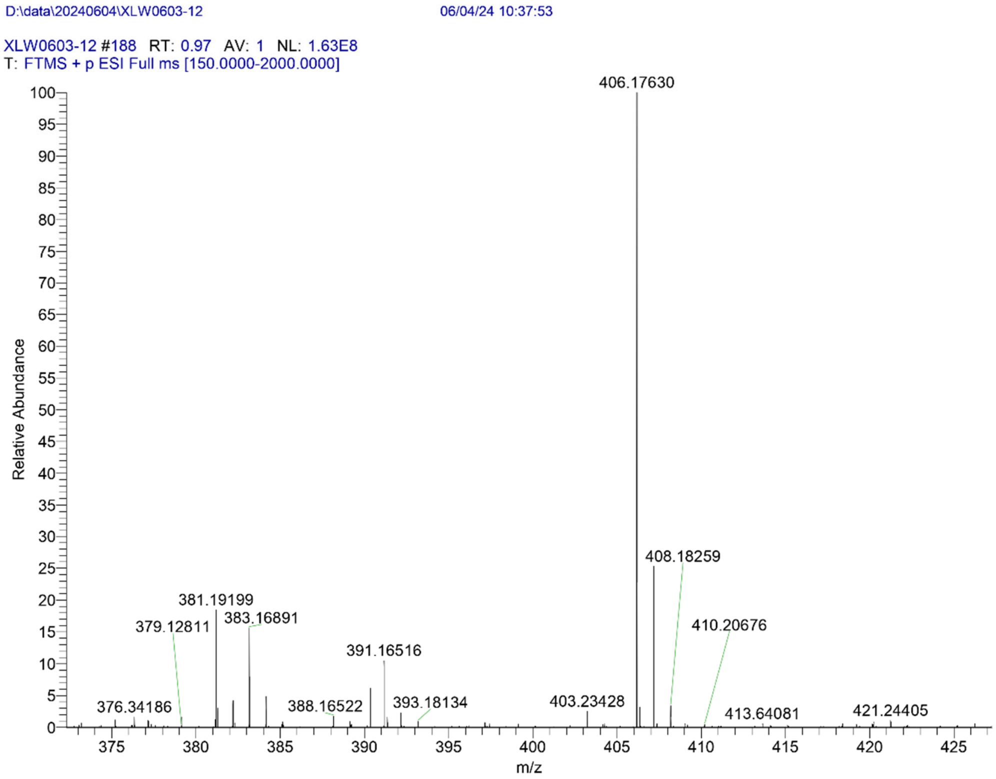

Fig. S39. LC-MS spectrum of compound **11** in methanol.

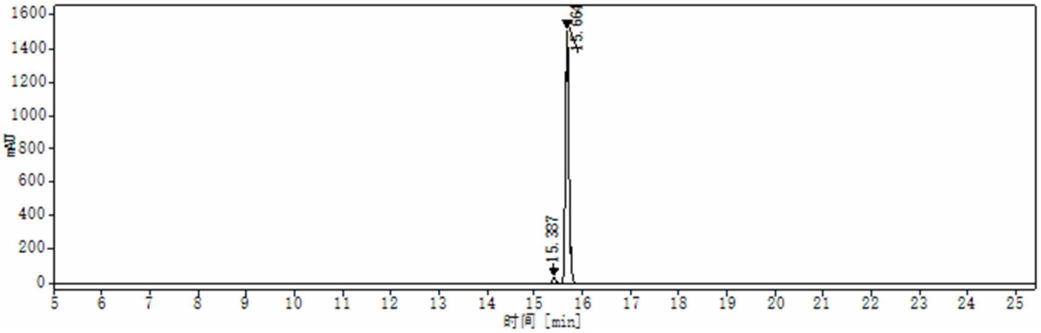

No.	Retention time	Peak area	Concentration (%)
1	15.387	188.75	2.45
2	15.664	7527.50	97.55

Fig. S40. HPLC purity of compound **11**

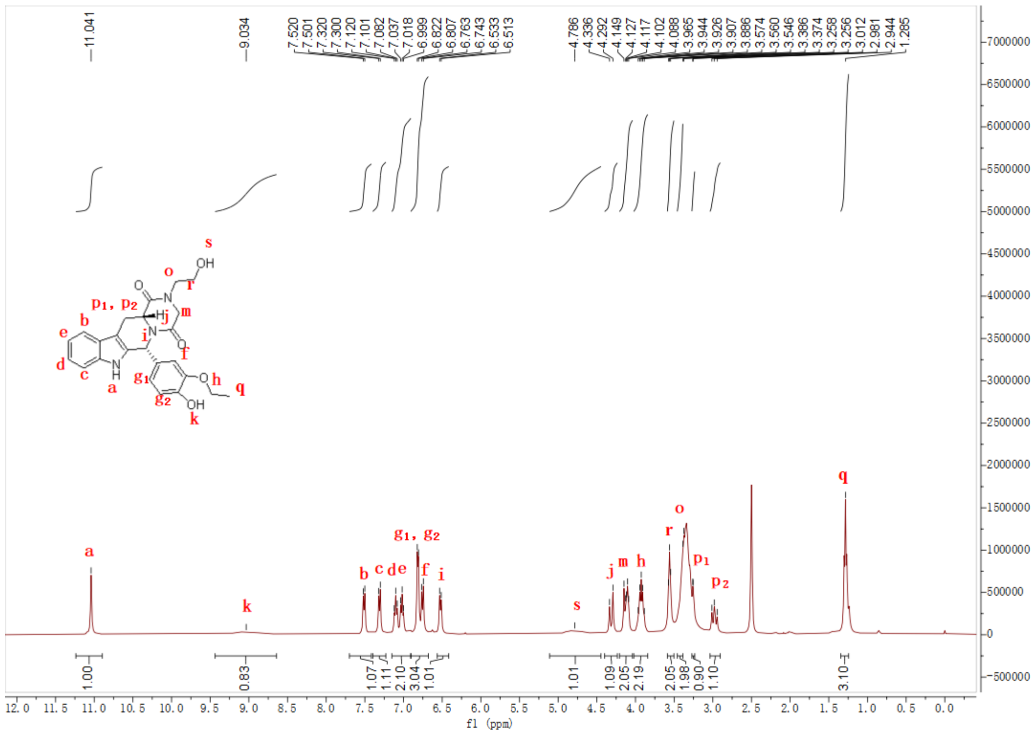

Fig. S41. ^{1}H-NMR spectrum of compound **12** in DMSO-d_6.

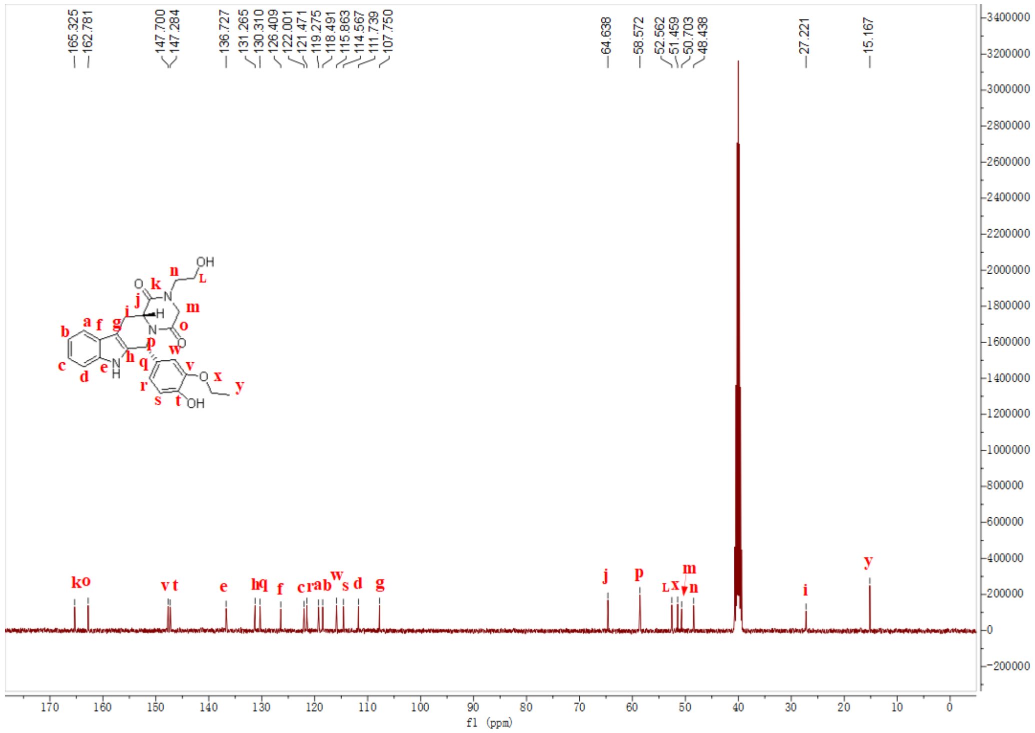

Fig. S42. ^{13}C-NMR spectrum of compound **12** in DMSO-d_6

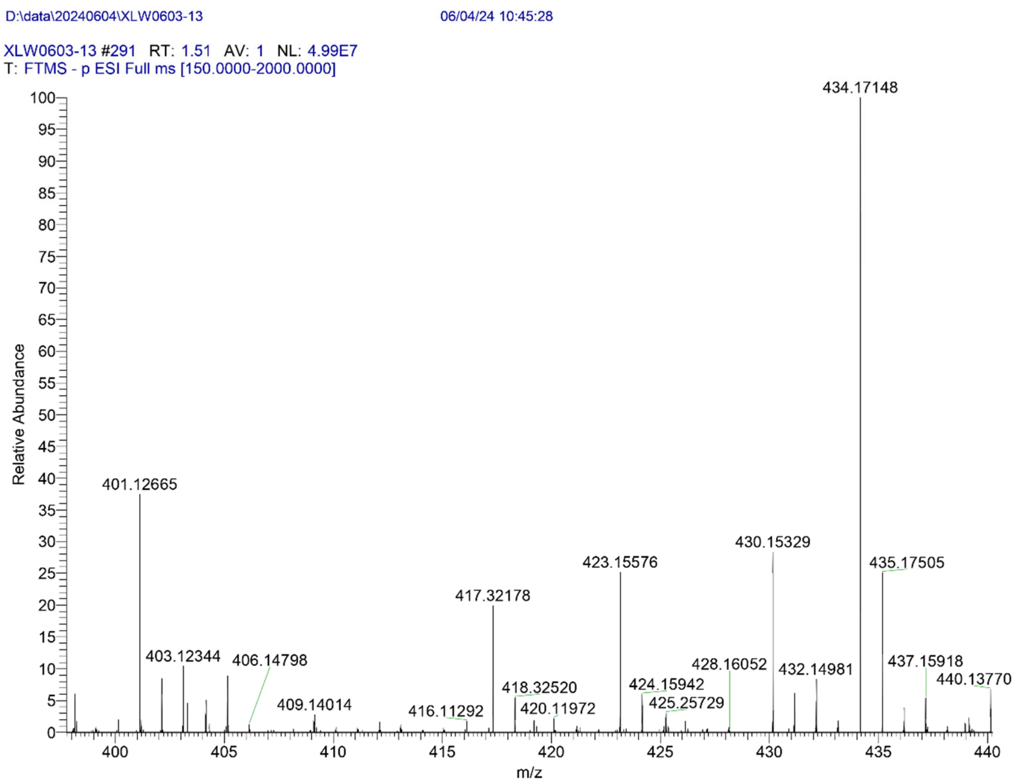

Fig. S43. LC-MS spectrum of compound **12** in methanol.

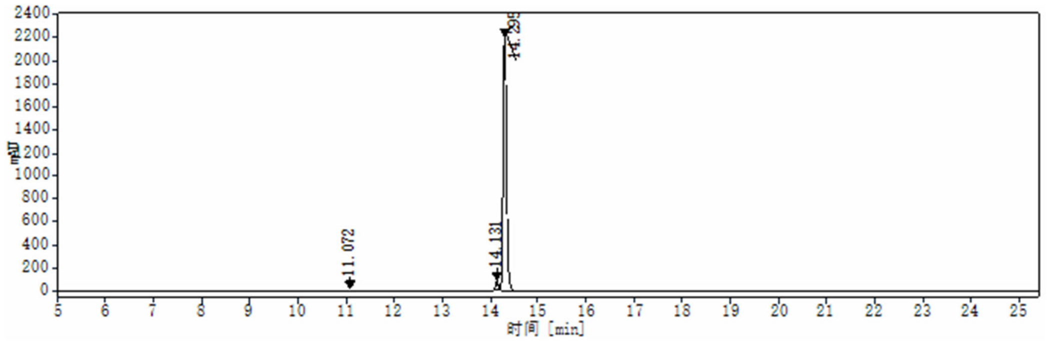

No.	Retention time	Peak area	Concentration (%)
1	11. 072	63. 28	0. 59
2	14. 131	299. 40	2. 79
3	14. 295	10371. 32	96. 62

Fig. S44. HPLC purity of compound **12**

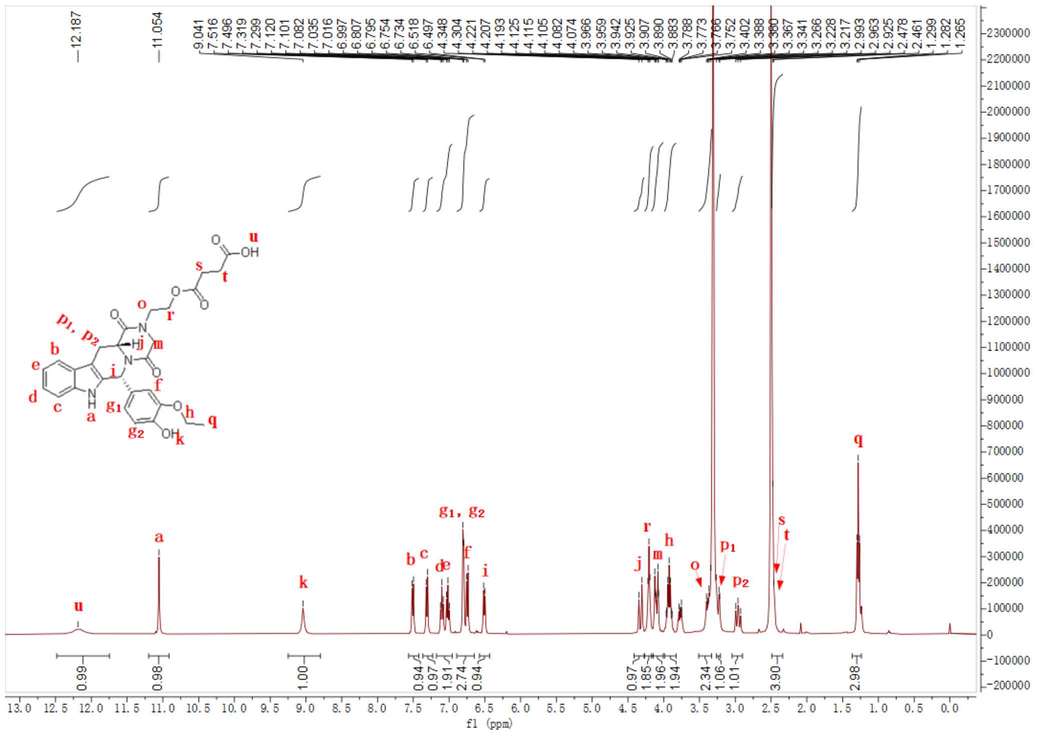

Fig. S45. ^{1}H-NMR spectrum of compound **13** in DMSO-*d*6.

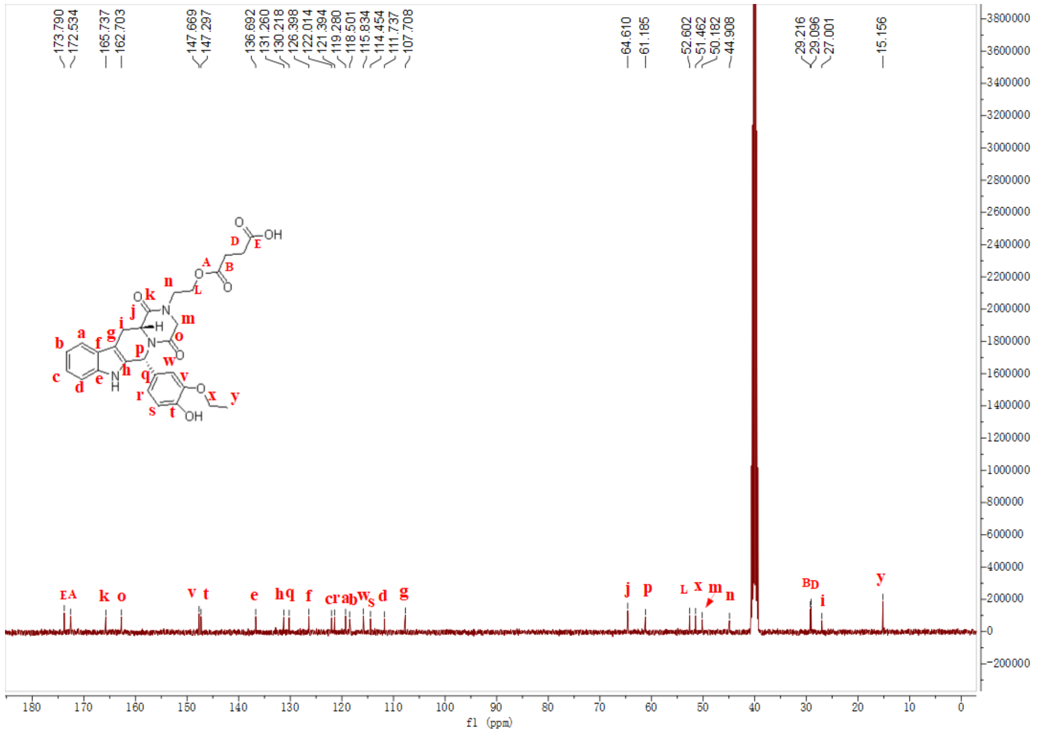

Fig. S46. ^{13}C-NMR spectrum of compound **13** in DMSO-*d*6

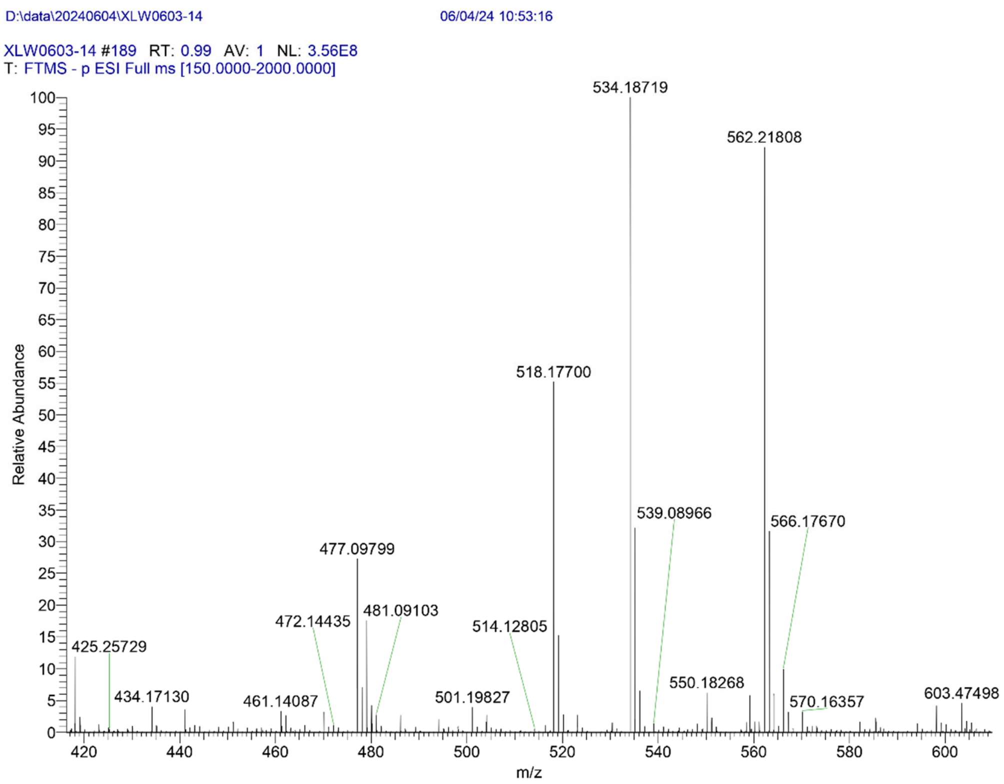

Fig. S47. LC-MS spectrum of compound **13** in methanol.

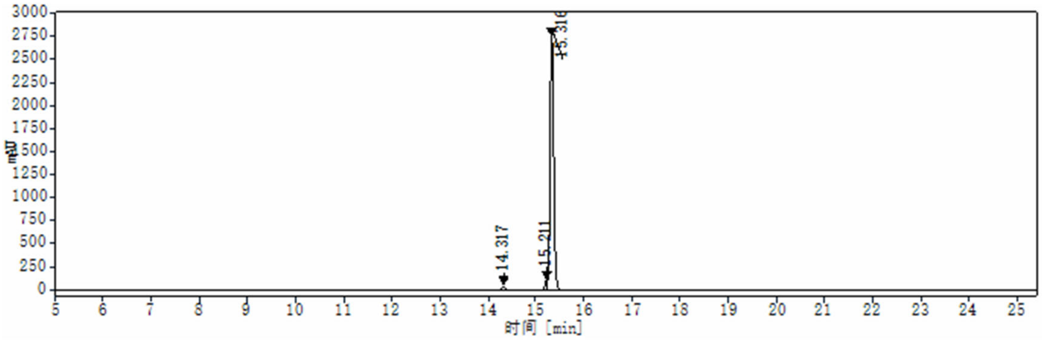

No.	Retention time	Peak area	Concentration (%)
1	14.317	167.86	1.14
2	15.211	262.08	1.78
3	15.316	14254.92	97.08

Fig. S48. HPLC purity of compound **13**

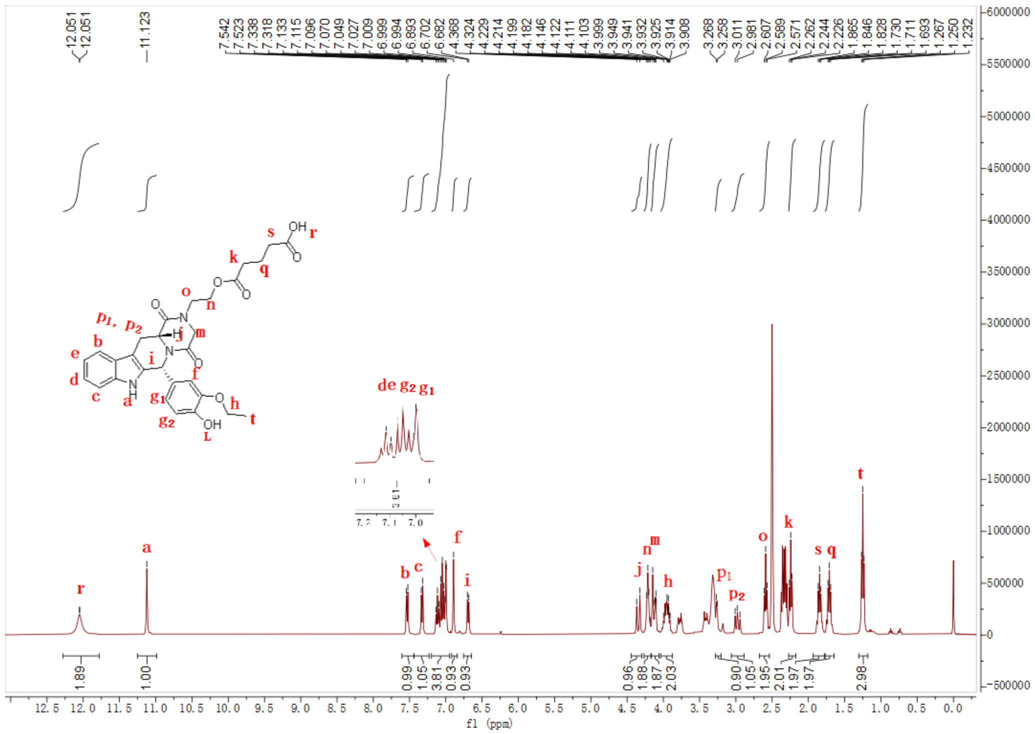

Fig. S49. ^{1}H-NMR spectrum of compound **14** in DMSO-d_6.

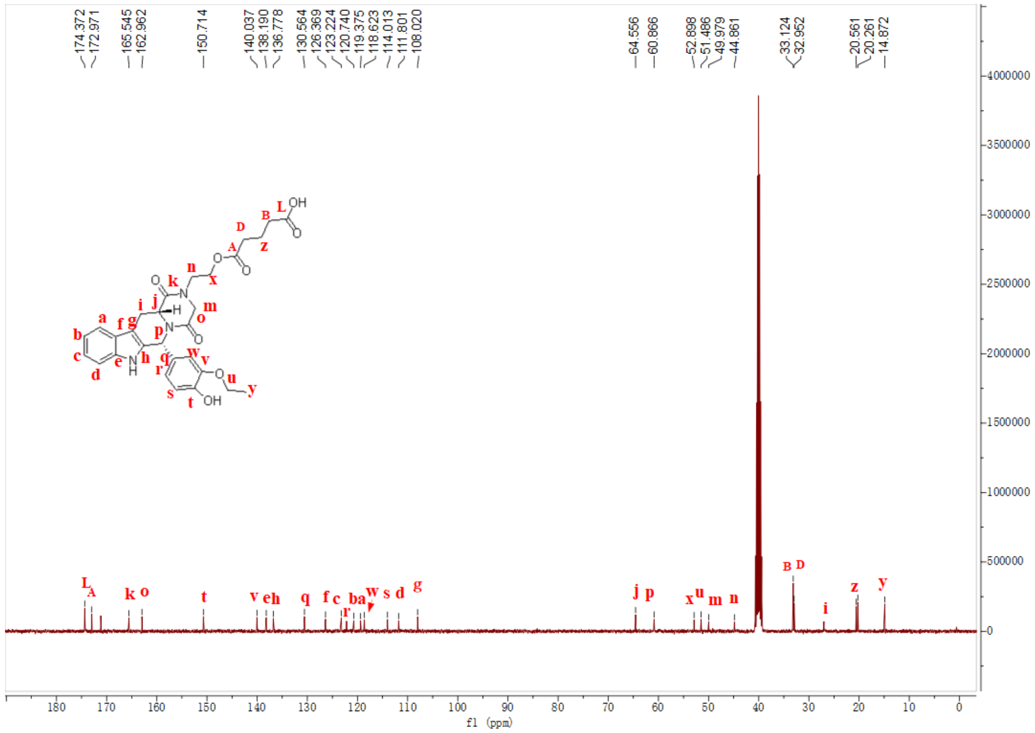

Fig. S50. ^{13}C-NMR spectrum of compound **14** in DMSO-d_6

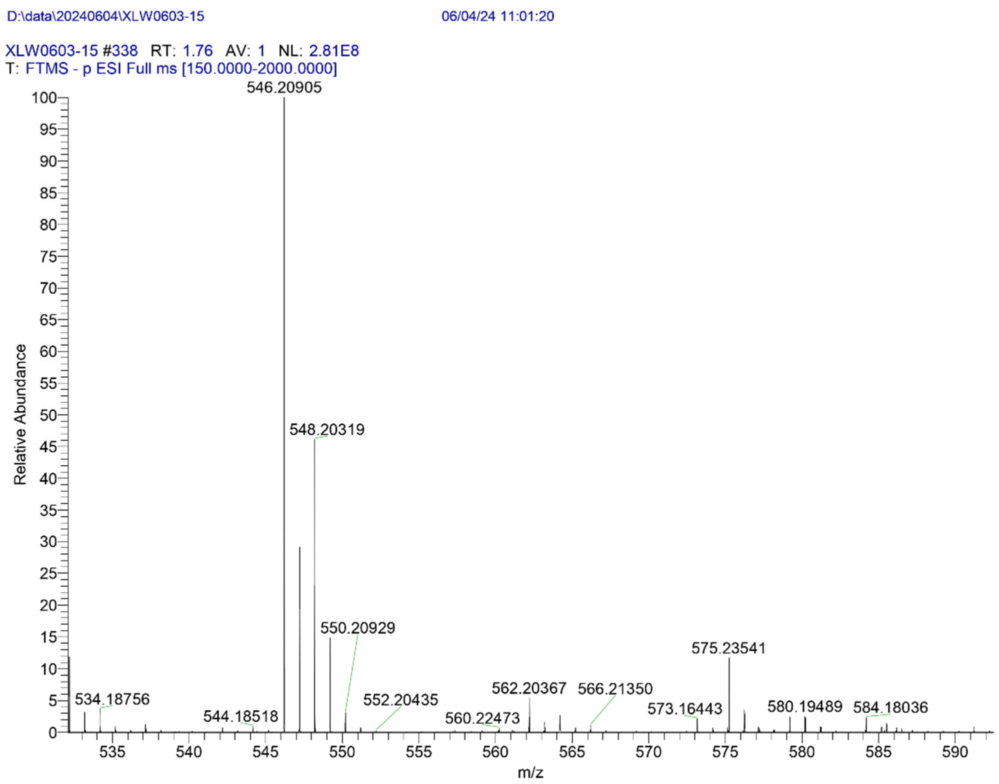

Fig. S51. LC-MS spectrum of compound **14** in methanol.

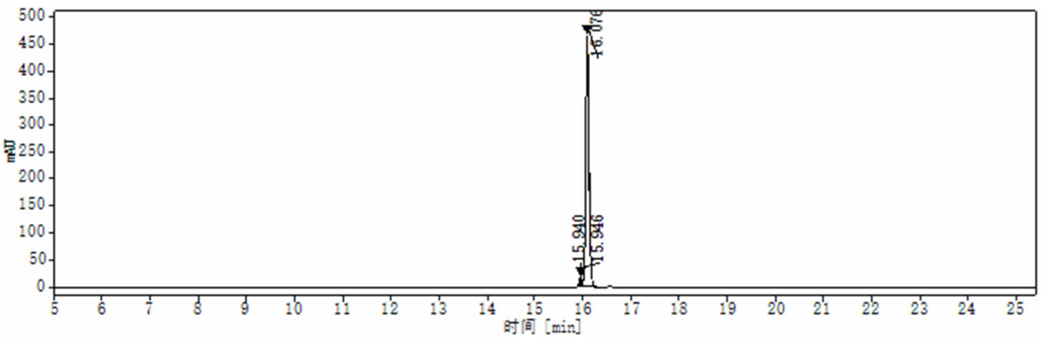

No.	Retention time	Peak area	Concentration (%)
1	15.940	31.87	1.41
2	15.946	35.83	1.59
3	16.076	2191.90	97.00

Fig. S52. HPLC purity of compound **14**

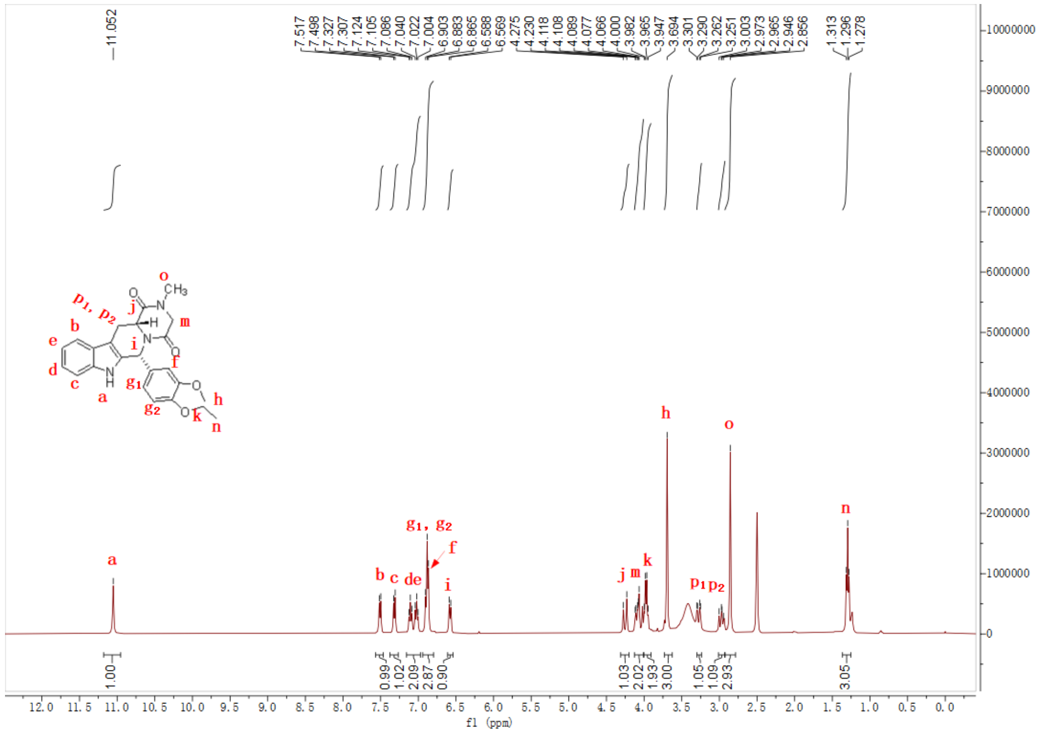

Fig. S53. ^{1}H-NMR spectrum of compound **15** in DMSO-*d*6.

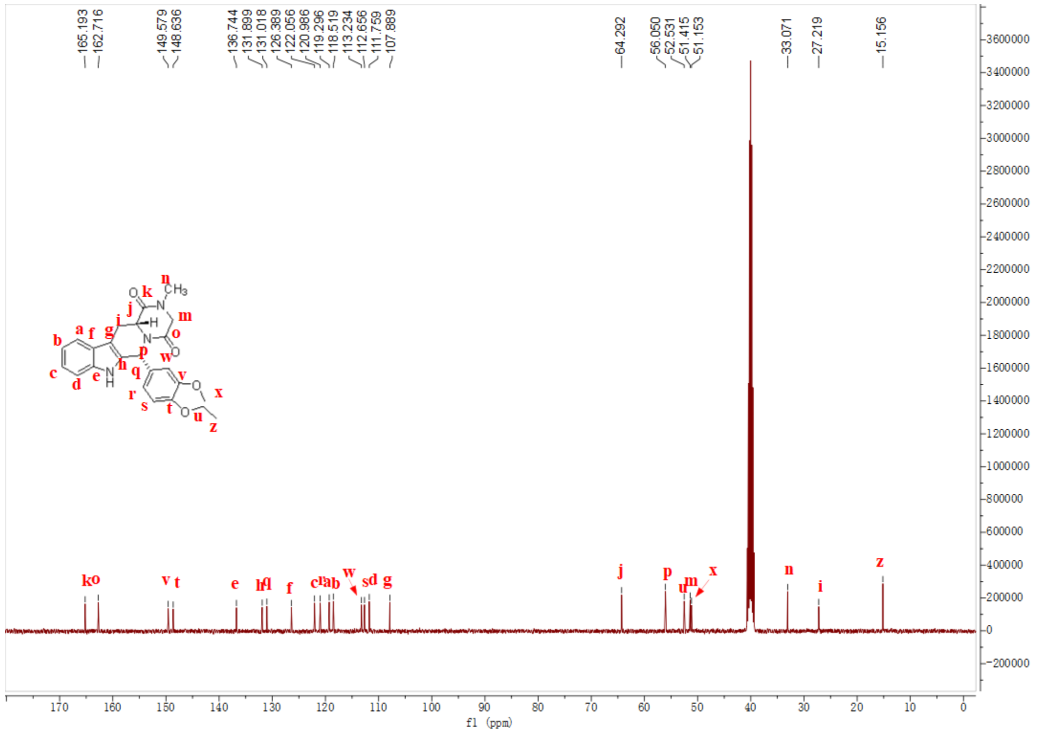

Fig. S54. ^{13}C-NMR spectrum of compound **15** in DMSO-*d*6

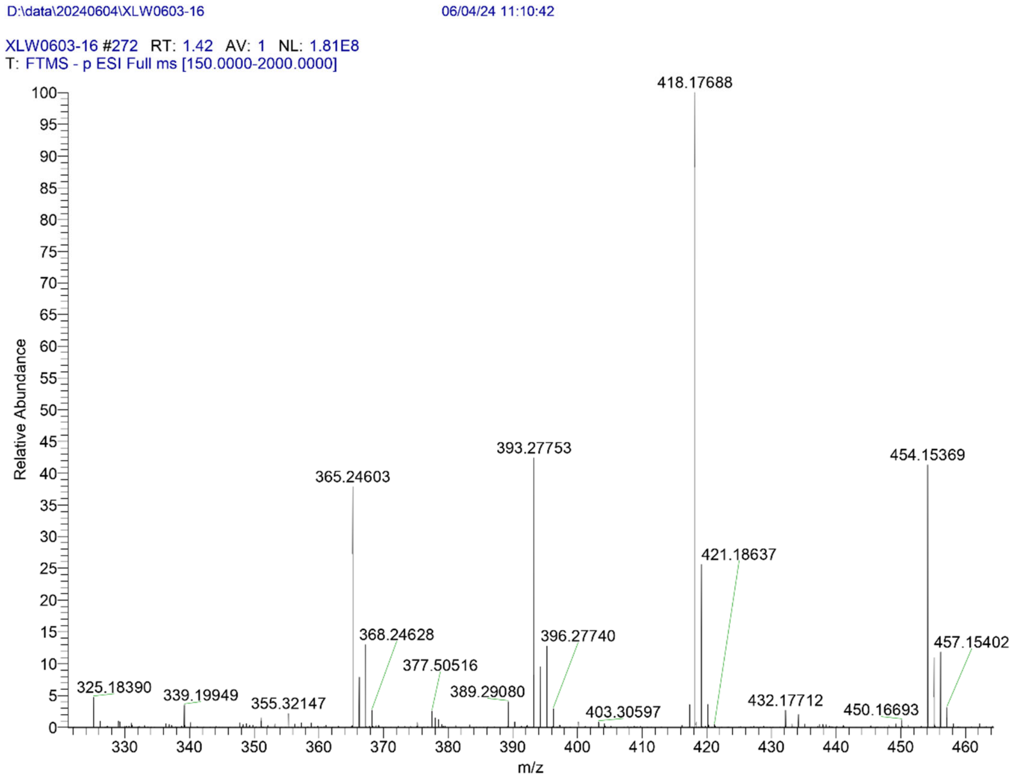

Fig. S55. LC-MS spectrum of compound **15** in methanol.

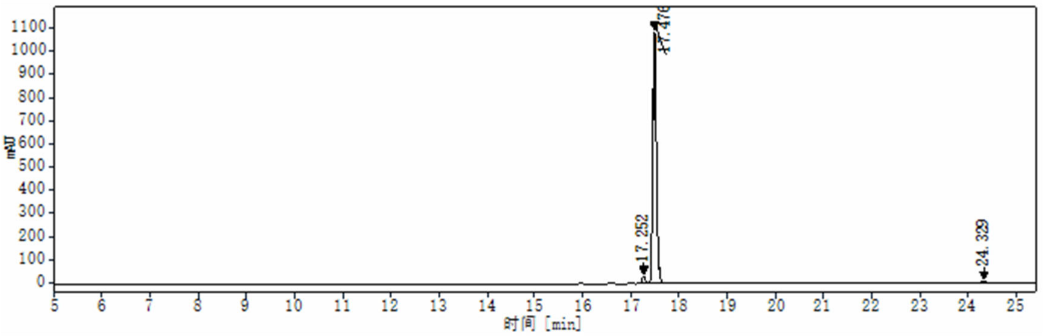

No.	Retention time	Peak area	Concentration (%)
1	17.252	138.51	2.37
2	17.476	5663.89	96.92
3	24.329	41.37	0.71

Fig. S56. HPLC purity of compound **15**

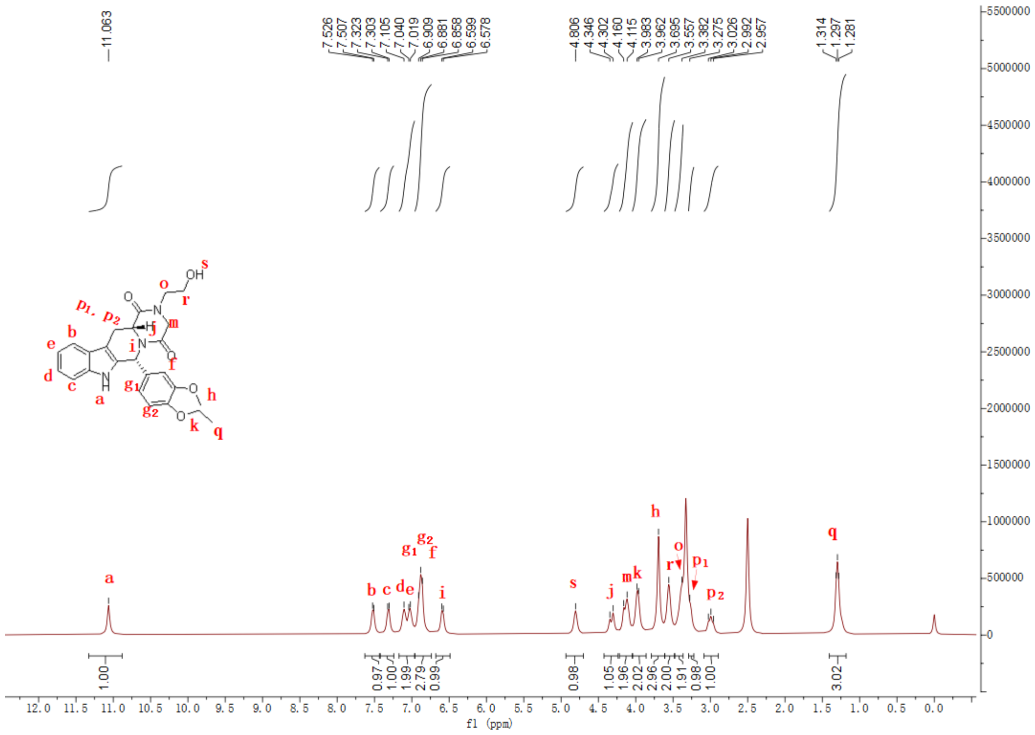

Fig. S57. ^{1}H-NMR spectrum of compound **16** in DMSO-*d*6.

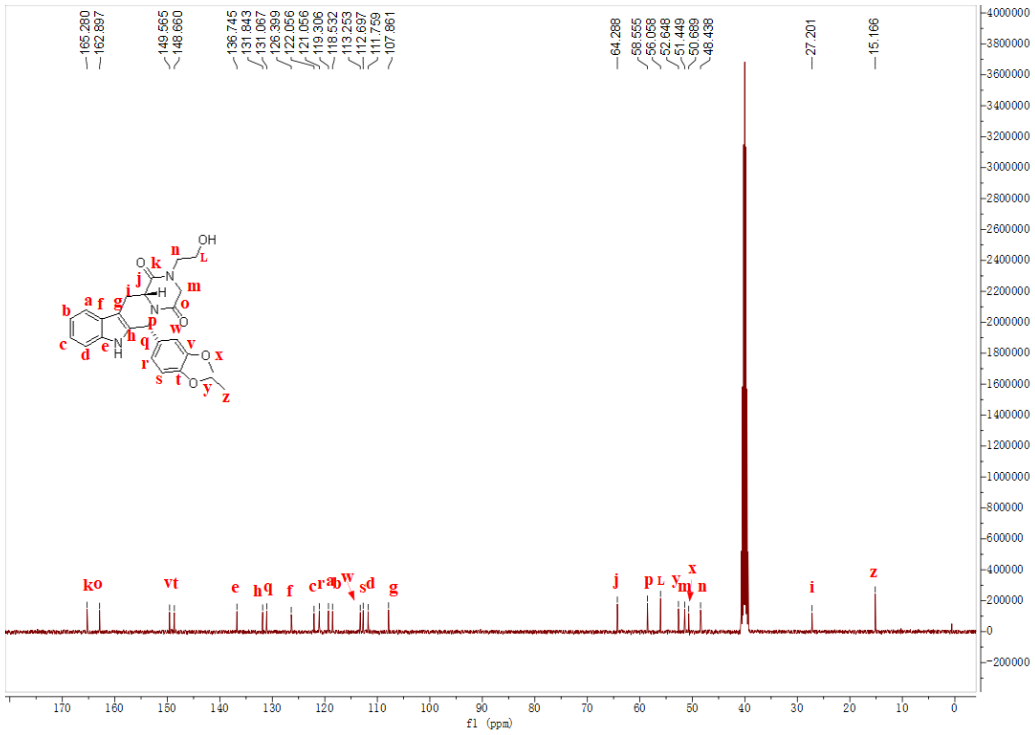

Fig. S58. ^{13}C-NMR spectrum of compound **16** in DMSO-*d*6

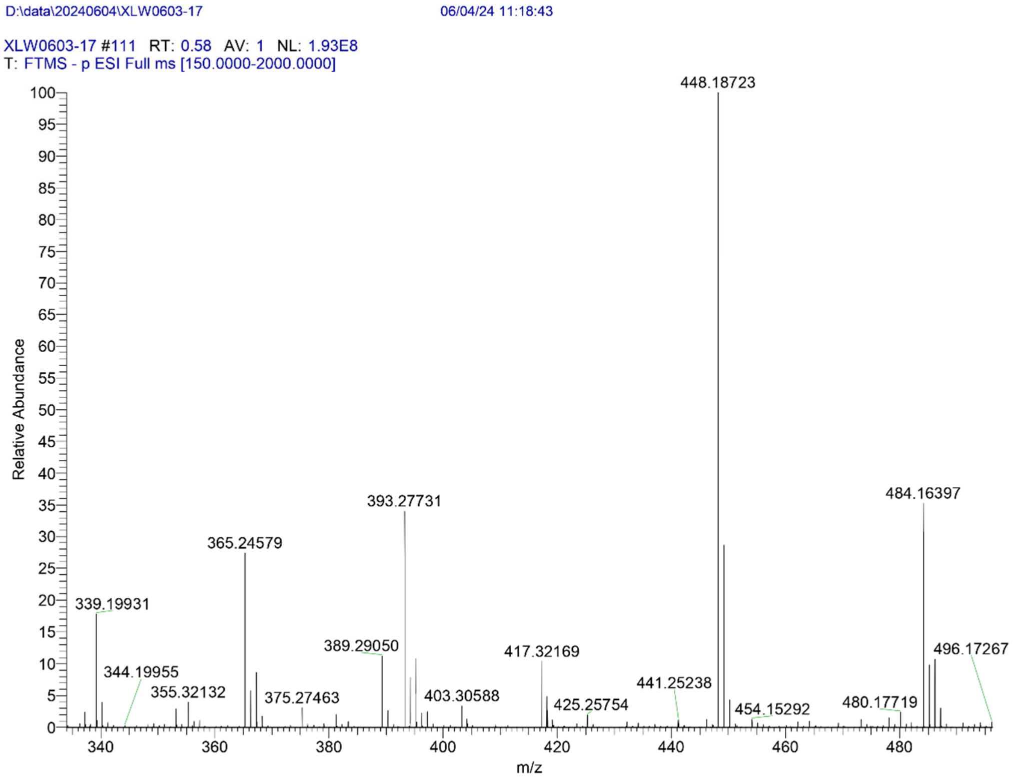

Fig. S59. LC-MS spectrum of compound **16** in methanol.

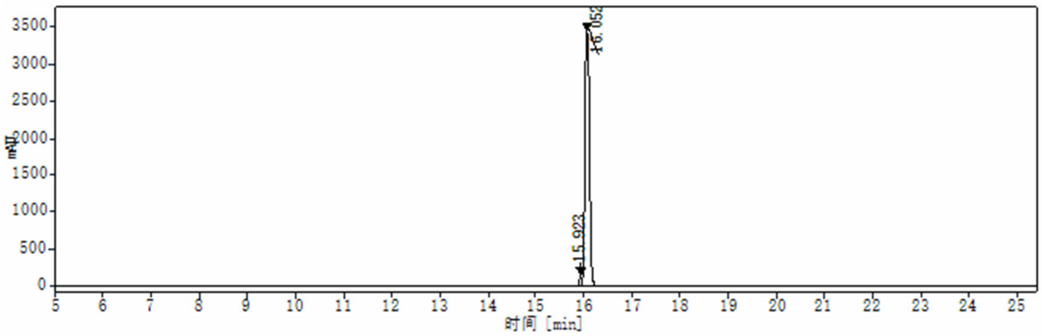

No.	Retention time	Peak area	Concentration (%)
1	15.923	495.17	2.16
2	16.052	22417.29	97.84

Fig. S60. HPLC purity of compound **16**

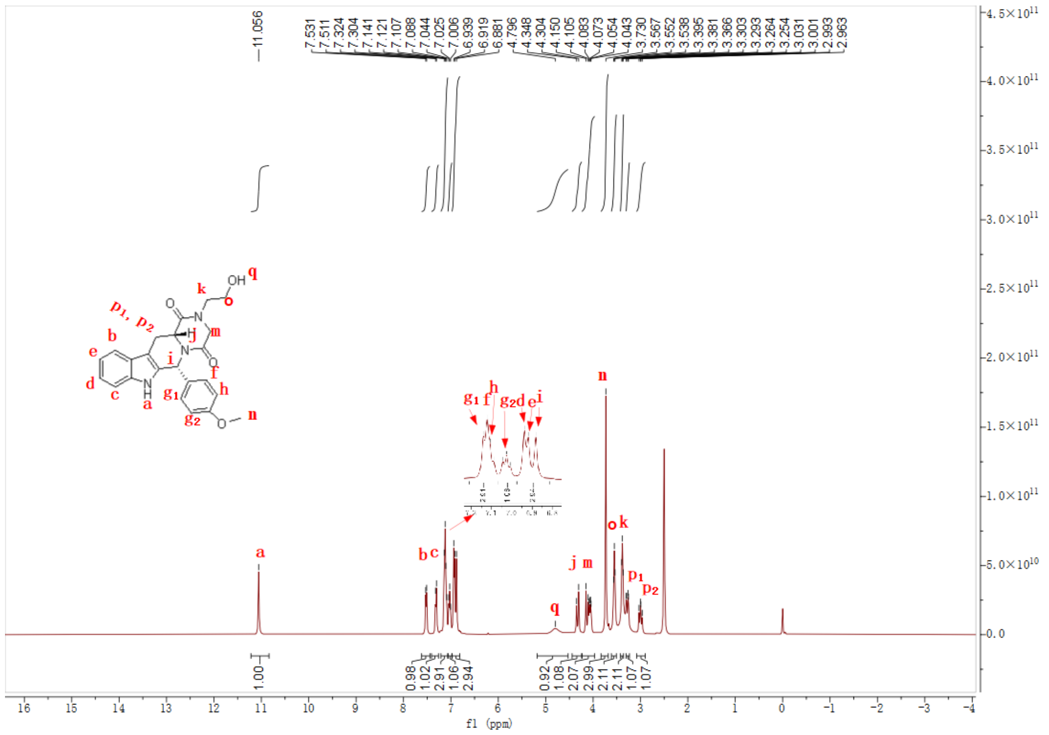

Fig. S61. ^{1}H-NMR spectrum of compound **17** in DMSO-*d*6.

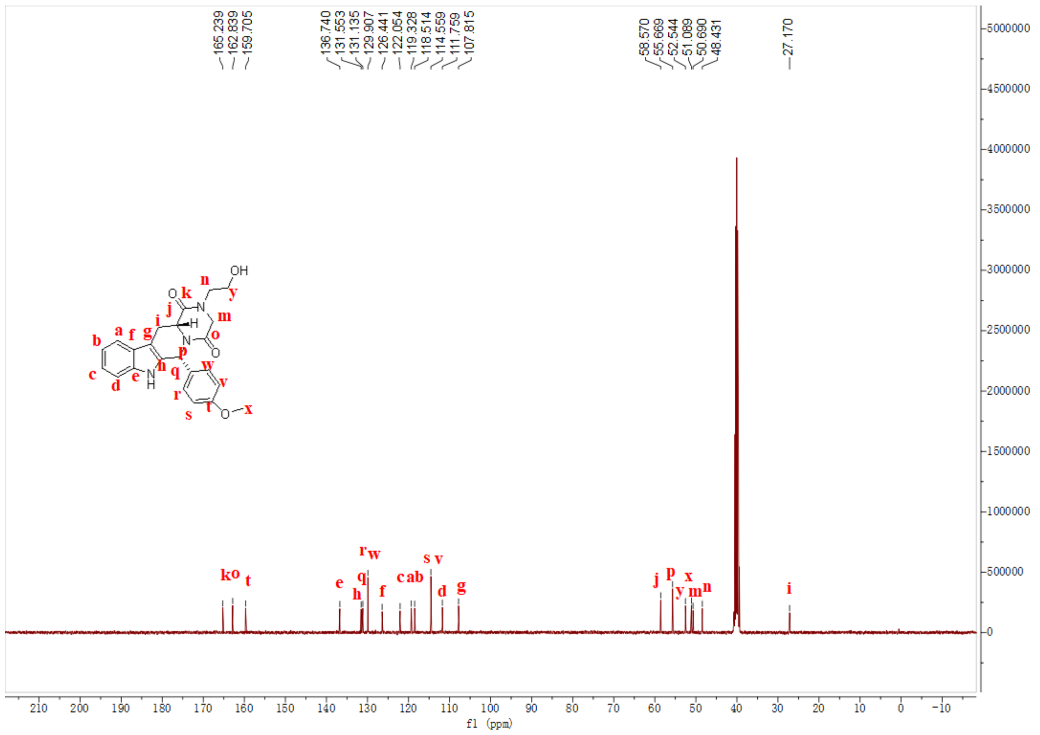

Fig. S62. ^{13}C-NMR spectrum of compound **17** in DMSO-*d*6

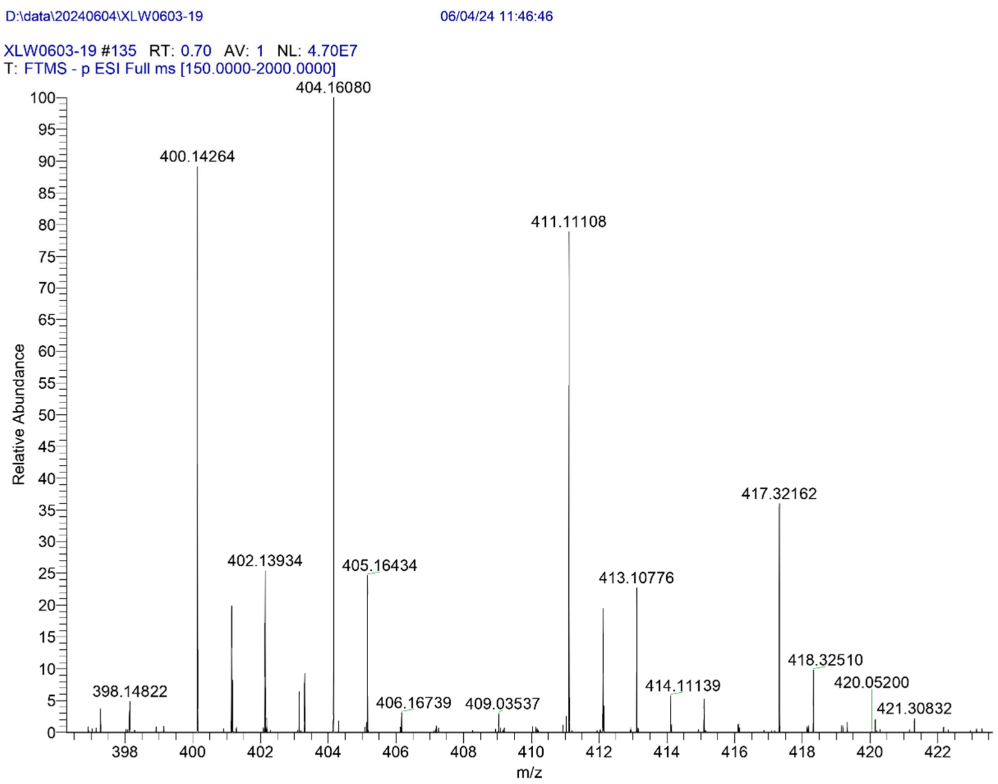

Fig. S63. LC-MS spectrum of compound **17** in methanol.

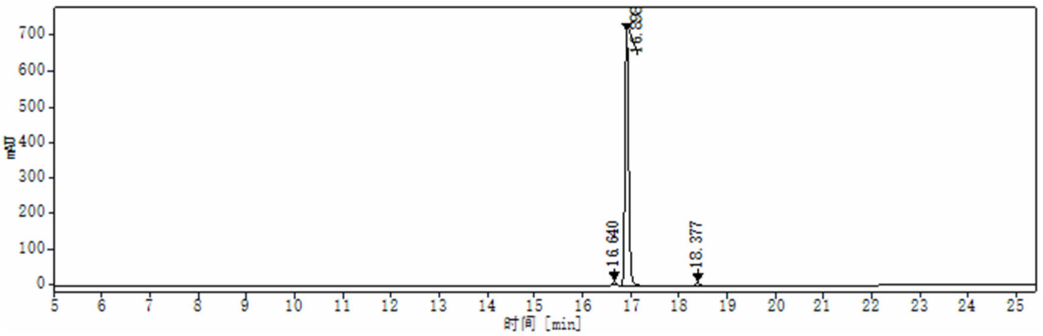

No.	Retention time	Peak area	Concentration (%)
1	16.640	34.36	0.92
2	16.898	3650.23	98.20
3	18.377	32.85	0.88

Fig. S64. HPLC purity of compound **17**

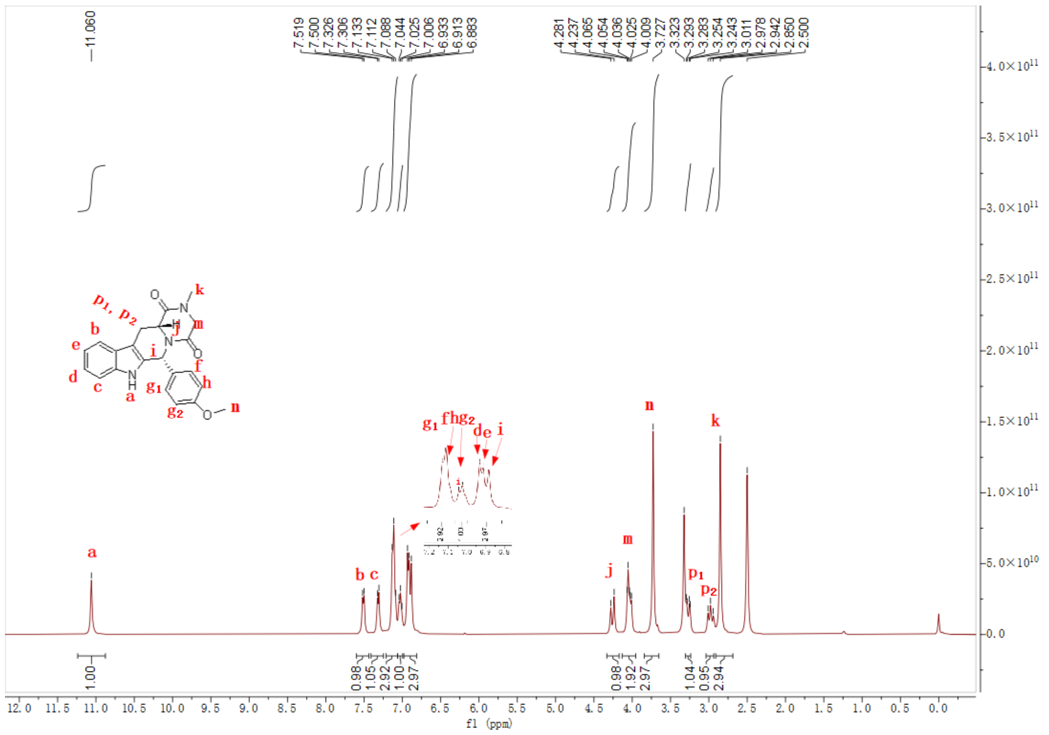

Fig. S65. ^{1}H-NMR spectrum of compound **18** in DMSO-*d*6.

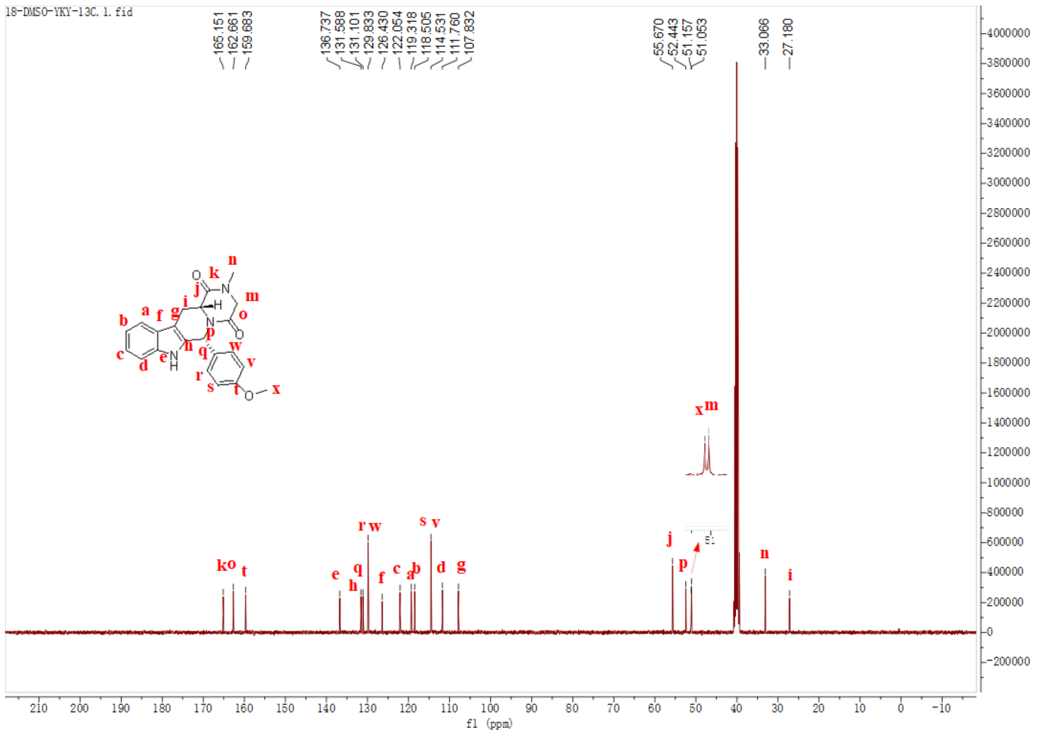

Fig. S66. ^{13}C-NMR spectrum of compound **18** in DMSO-*d*6

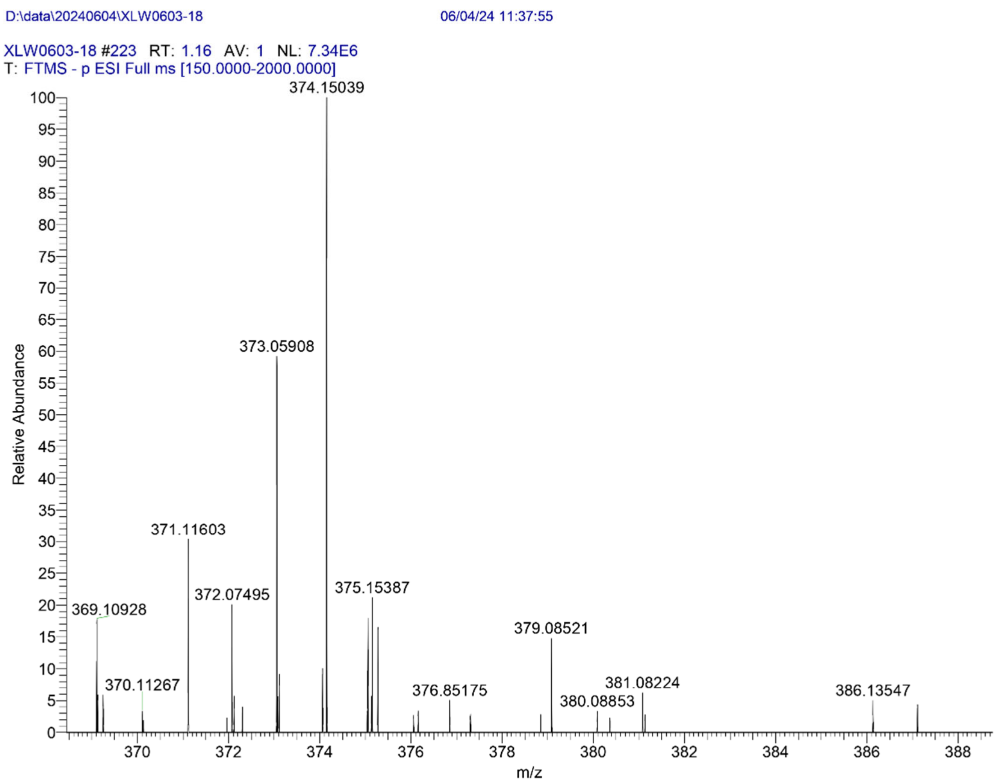

Fig. S67. LC-MS spectrum of compound **18** in methanol.

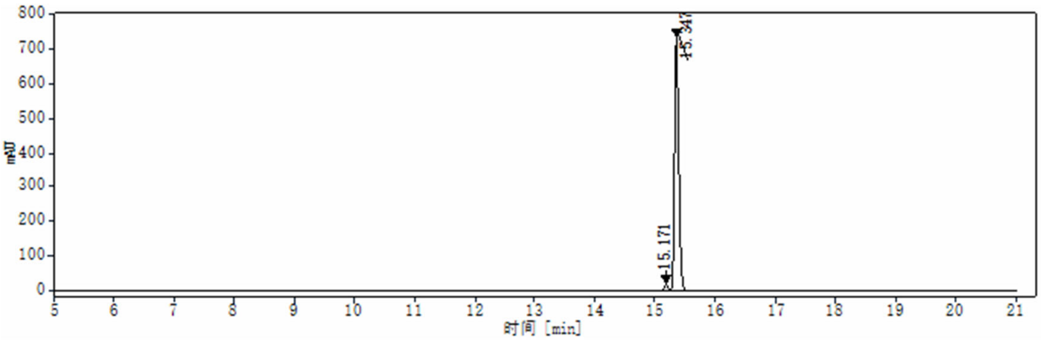

No.	Retention time	Peak area	Concentration (%)
1	15.171	66.02	1.86
2	15.347	3483.29	98.14

Fig. S68. HPLC purity of compound **18**

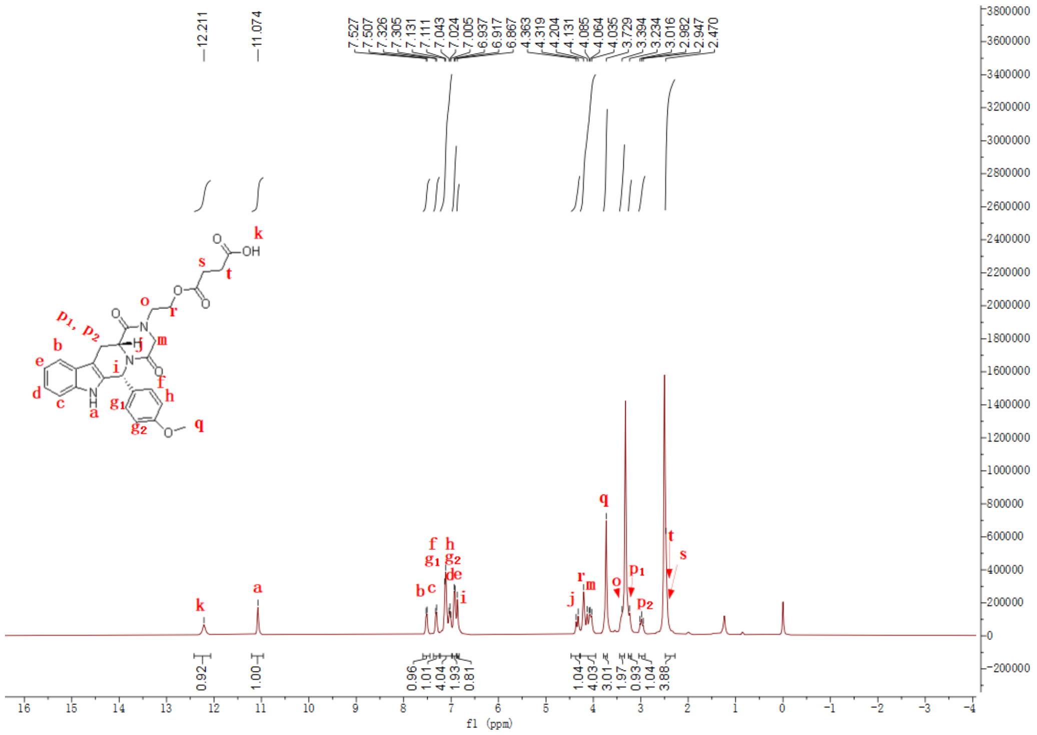

Fig. S69. ^{1}H-NMR spectrum of compound **19** in DMSO-d_6.

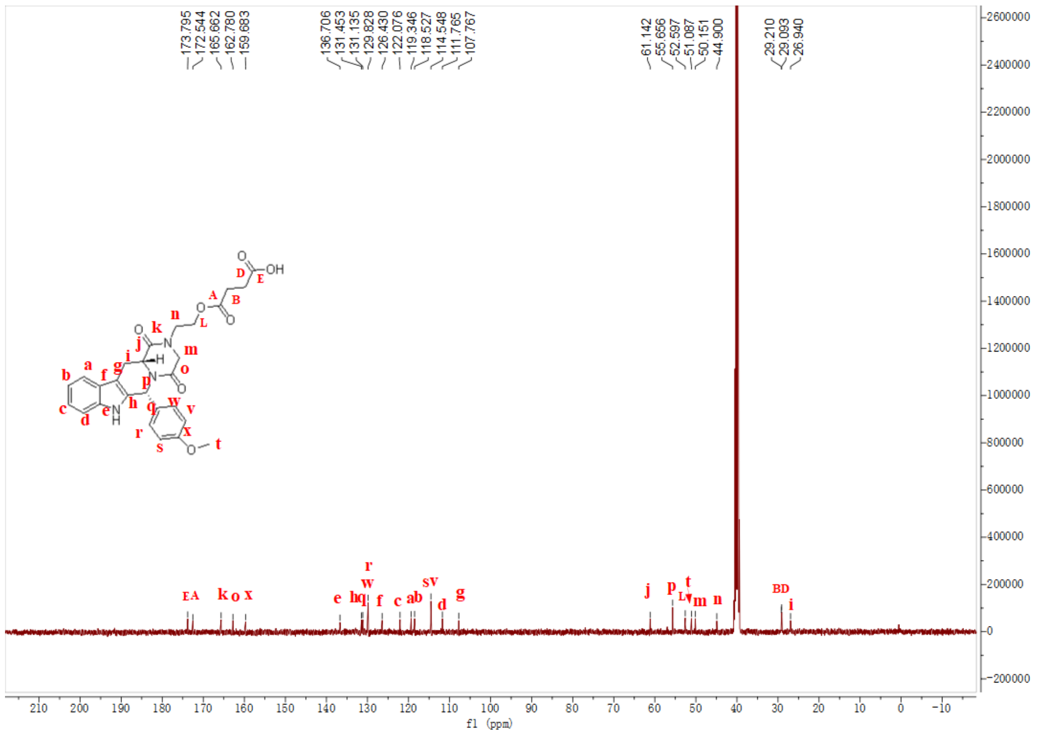

Fig. S70. ^{13}C-NMR spectrum of compound **19** in DMSO-d_6

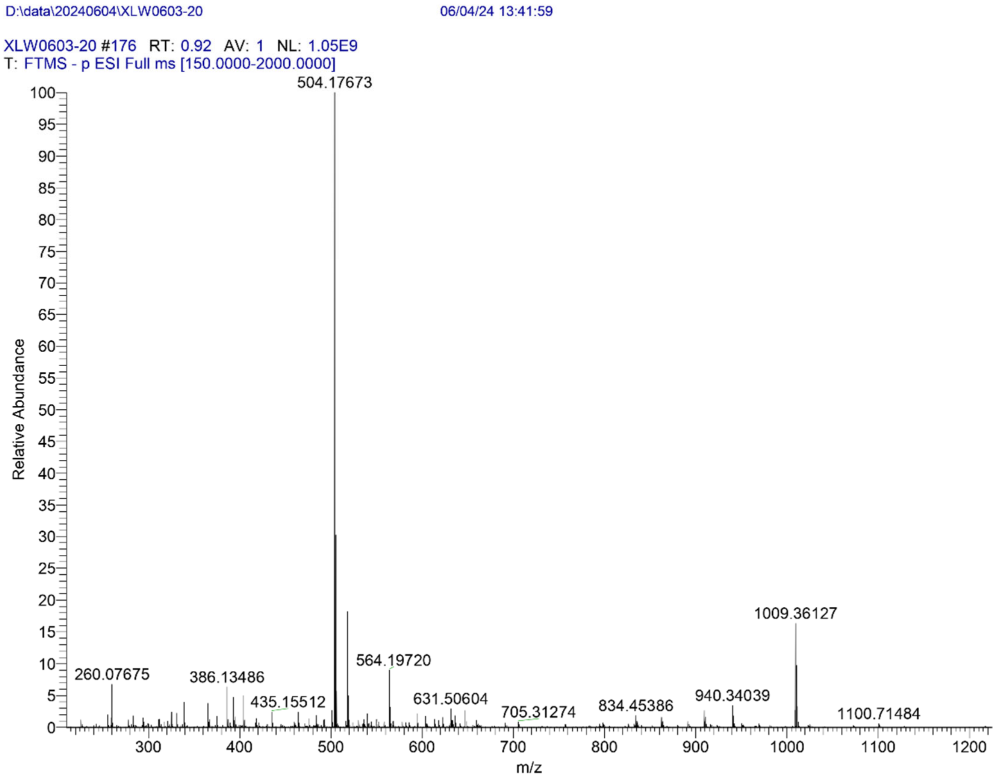

Fig. S71. LC-MS spectrum of compound **19** in methanol.

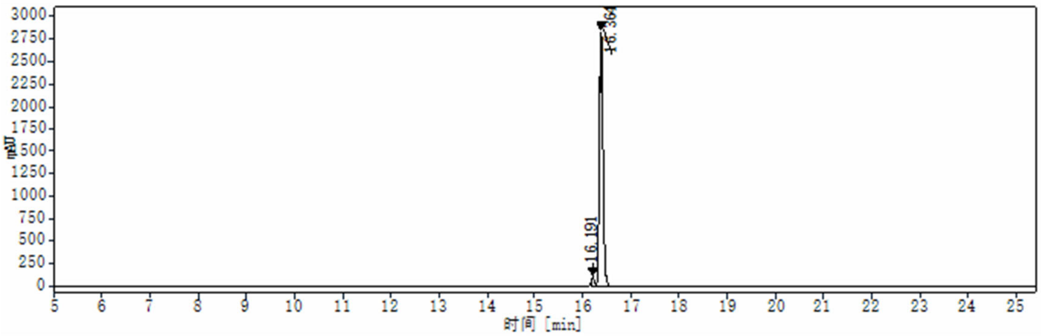

No.	Retention time	Peak area	Concentration (%)
1	16.191	432.84	2.84
2	16.364	14800.51	97.16

Fig. S72. HPLC purity of compound **19**

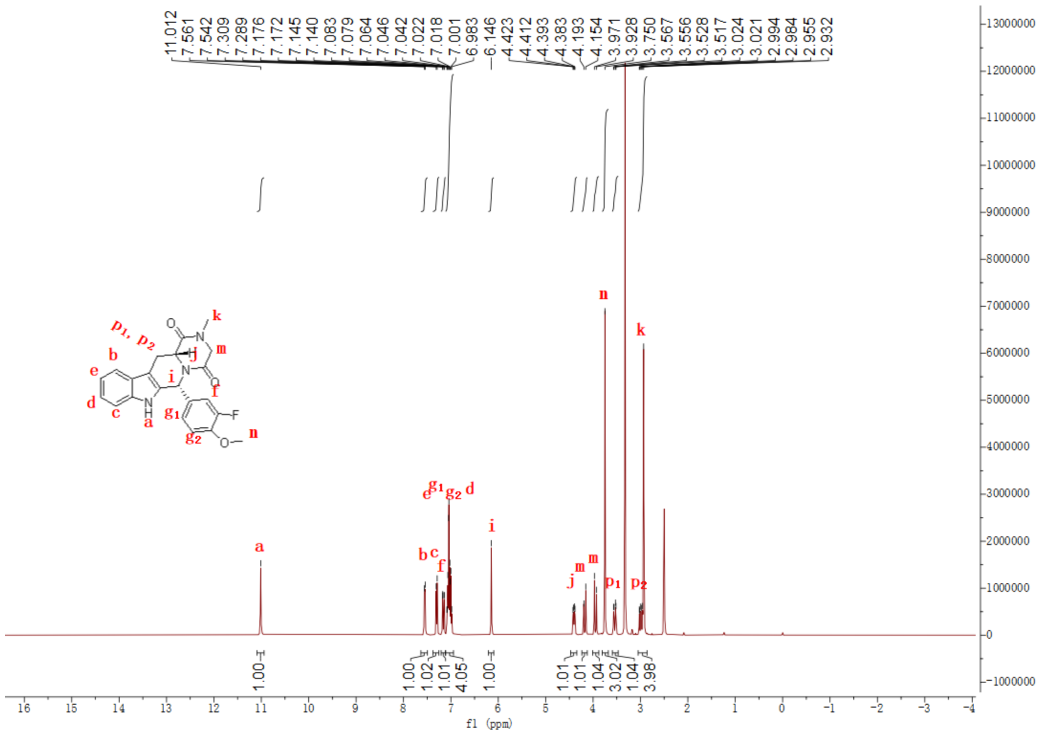

Fig. S73. ^{1}H-NMR spectrum of compound **20** in DMSO-d_6.

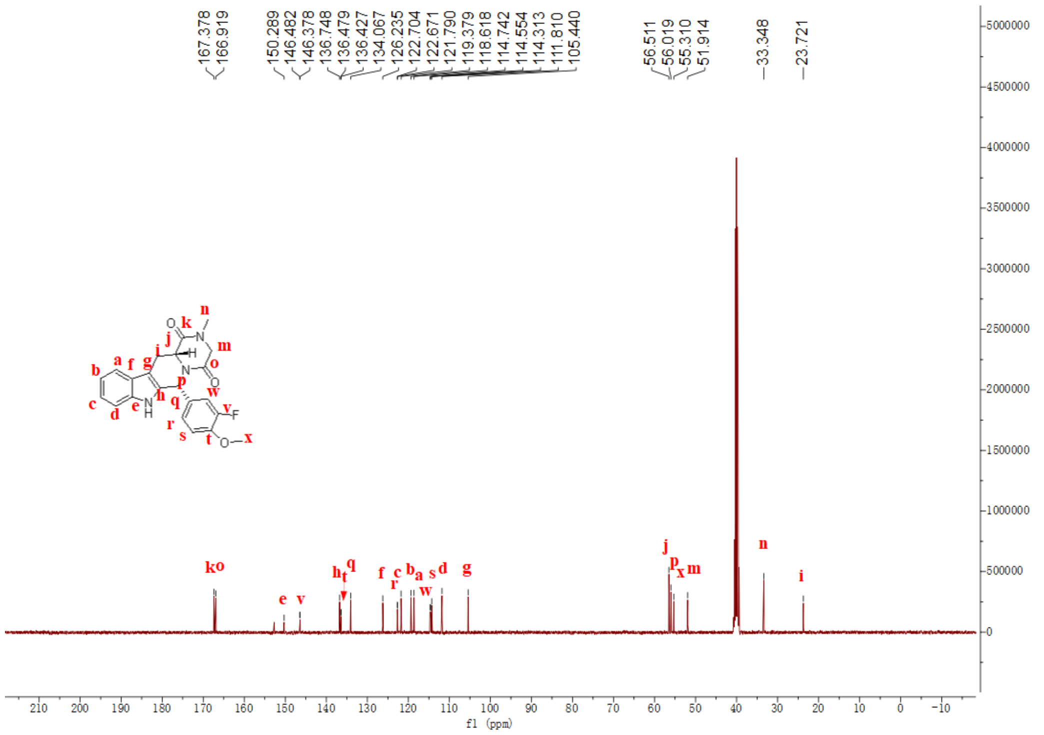

Fig. S74. ^{13}C-NMR spectrum of compound **20** in DMSO-d_6

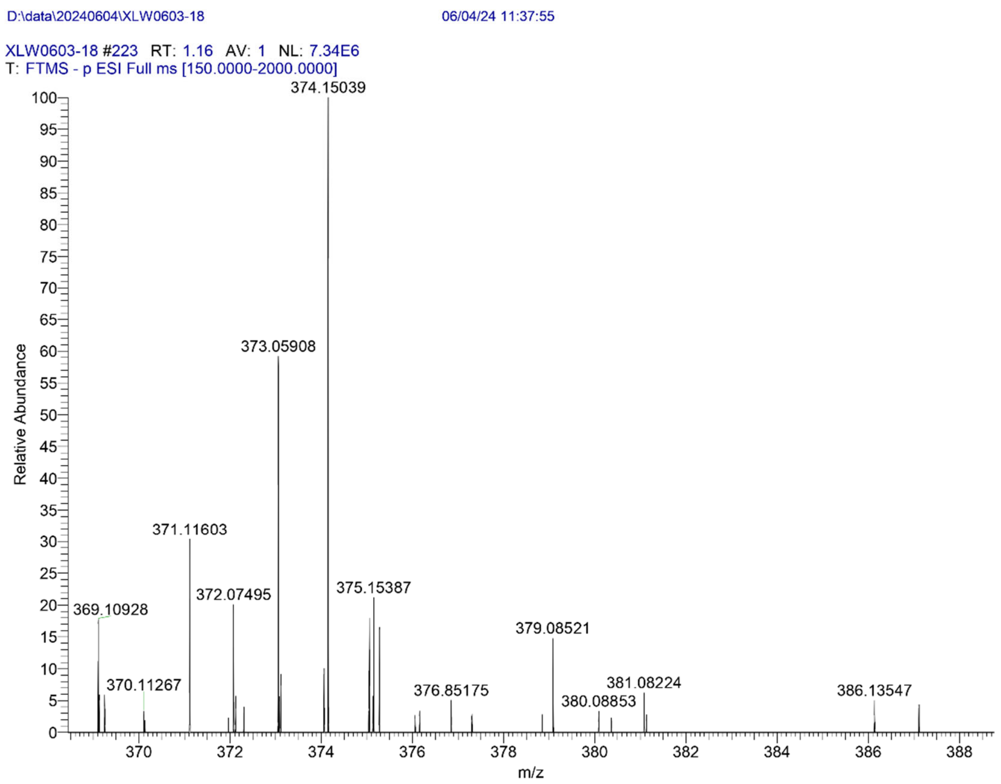

Fig. S75. LC-MS spectrum of compound **20** in methanol.

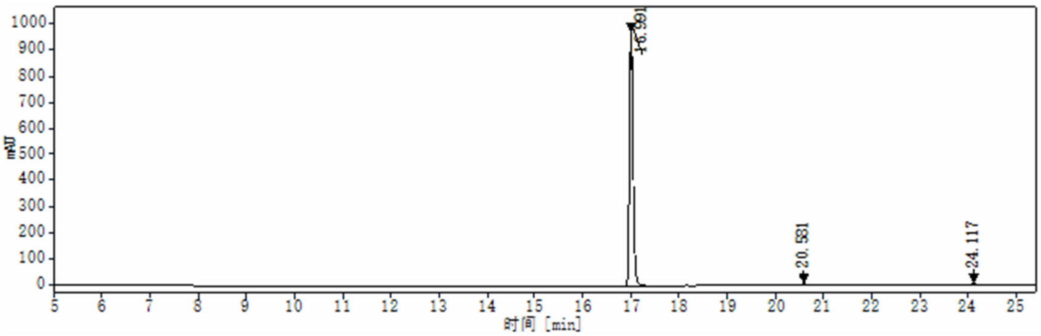

No.	Retention time	Peak area	Concentration (%)
1	16.991	4942.19	98.96
2	20.581	30.22	0.60
3	24.117	22.16	0.44

Fig. S76. HPLC purity of compound **20**
